# Menstrual health interventions, schooling, and mental health problems among Ugandan students (MENISCUS): study protocol for a school-based cluster-randomised trial

**DOI:** 10.1186/s13063-022-06672-4

**Published:** 2022-09-07

**Authors:** Catherine Kansiime, Laura Hytti, Kate Andrews Nelson, Belen Torondel, Suzanna C. Francis, Clare Tanton, Giulia Greco, Sophie Belfield, Shamirah Nakalema, Fred Matovu, Andrew Sentoogo Ssemata, Connie Alezuyo, Stella Neema, John Jerrim, Chris Bonell, Janet Seeley, Helen A. Weiss, MENISCUS Group, Aggrey Tumuhimbise, Christopher Baleke, Denis Ndekezi, Denis Ssenyondwa, Kevin Nakuya, Levicatus Mugenyi, Prossy Namirembe, Ratifah Batuusa, Robert Bakanoma, Stephen Lagony, Titus Kisa Ssesanga

**Affiliations:** 1 MRC/UVRI and LSHTM Uganda Research Unit, P.O. Box 49, Plot 51-59 Nakiwogo Road, Entebbe, Uganda; 2 LSHTM, London, UK; 3 Faculty of Infectious and Tropical Diseases, LSHTM, London, UK; 4 WoMena Uganda, Kampala, Uganda; 5 PADRI, Makerere University, Kampala, Uganda; 6 Education Response Plan Secretariat, Ministry of Education and Sports, Kampala, Uganda; 7 Makerere University, Kampala, Uganda; 8 UCL Institute of Education, University College London, London, UK; 9 Social Science Programme, MRC/UVRI and LSHTM, Entebbe, Uganda

**Keywords:** Menstrual health, Mental health, School-based intervention, Adolescent health, Education

## Abstract

**Background:**

Menstrual health is an increasingly recognised public health issue, defined as complete physical, mental, and social well-being in relation to the menstrual cycle. The MENISCUS trial aims to assess whether a multi-component intervention addressing physical and emotional aspects of menstrual health improves educational attainment, mental health problems, menstrual management, self-efficacy, and quality of life among girls in secondary school in Uganda.

**Methods:**

The study is a parallel-arm cluster-randomised controlled trial with 60 schools (clusters) in Wakiso and Kalungu districts, with a mixed-methods process evaluation to assess intervention fidelity and acceptability and economic and policy analyses. The schools will be randomised 1:1 to immediate intervention or to optimised usual care with delayed intervention delivery. The intervention includes creation of a Menstrual Health Action Group at schools and NGO-led training of trainers on puberty education, development of a drama skit, delivery of a menstrual health kit including reusable pads and menstrual cups, access to pain management strategies including analgesics, and basic improvements to school water, sanitation, and hygiene facilities. Baseline data will be collected from secondary 2 students in 2022 (median age ~15.5 years), with endline after 1 year of intervention delivery (~3600 females and a random sample of ~900 males).

The primary outcomes assessed in girls are (i) examination performance based on the Mathematics, English, and Biology curriculum taught during the intervention delivery (independently assessed by the Uganda National Examinations Board) and (ii) mental health problems using the Total Difficulties Scale of the Strengths and Difficulties 25-item questionnaire. Secondary outcomes are menstrual knowledge and attitudes in girls and boys and, in girls only, menstrual practices, self-efficacy in managing menstruation, quality of life and happiness, prevalence of urogenital infections, school and class attendance using a self-completed menstrual daily diary, and confidence in maths and science.

**Discussion:**

The trial is innovative in evaluating a multi-component school-based menstrual health intervention addressing both physical and emotional aspects of menstrual health and using a “training of trainers” model designed to be sustainable within schools. If found to be cost-effective and acceptable, the intervention will have the potential for national and regional scale-up.

**Trial registration:**

ISRCTN 45461276. Registered on 16 September 2021

**Supplementary Information:**

The online version contains supplementary material available at 10.1186/s13063-022-06672-4.

## Administrative information

Note: the numbers in curly brackets in this protocol refer to SPIRIT checklist item numbers. The order of the items has been modified to group similar items (see http://www.equator-network.org/reporting-guidelines/spirit-2013-statement-defining-standard-protocol-items-for-clinical-trials/).

**Table:** 

Title {1}	Menstrual health interventions, schooling and mental health problems among Ugandan students (MENISCUS): Study protocol for a school-based cluster-randomised trial.
Trial registration {2a and 2b}.	International Standard Randomised Controlled Trial Number (ISRCTN): 45461276, registered on 16^th^ September 2021.
Protocol version {3}	Version 3.0: January 2022
Funding {4}	Department of Health and Social Care (DHSC) through the National Institute for Health Research (NIHR), Foreign, Commonwealth and Development Office (FCDO), the Medical Research Council (MRC) and the Wellcome Trust through the Joint Global Health Trials scheme.
Author details {5a}	1. Dr Catherine Kansiime, MRC/UVRI and LSHTM Uganda Research Unit, Uganda Research Unit 2. Ms Laura Hytti, Independent Researcher, LSHTM, UK. 3. Ms Kate Andrews Nelson, MRC International Statistics and Epidemiology Group, London School of Hygiene & Tropical Medicine (LSHTM), UK. 4. Dr Belen Torondel, Faculty of Infectious and Tropical Diseases, LSHTM, UK. 5. Dr Suzanna C Francis, MRC International Statistics and Epidemiology Group, LSHTM, UK 6. Dr Clare Tanton, Faculty of Public Health and Policy, LSHTM, UK. 7. Dr Giulia Greco, Faculty of Public Health and Policy, LSHTM, UK. 8. Ms Sophie Belfield Research and Innovations Project Manager, WoMena Uganda, Email: sophie.belfield@womena.dk 9. Ms Shamirah Nakalema, Head of Programs, WoMena Uganda 10. Dr Fred Matovu, Makerere University, Uganda 11. Dr Andrew Sentoogo Ssemata, MRC/UVRI and LSHTM Uganda Research Unit, Plot 51-59 Nakiwogo Road Entebbe, Uganda, 12. Ms Connie Alezuyo, Coordinator, Education Response Plan Secretariat, Ministry of Education and Sports, Uganda 13. Dr Stella Neema, Makerere University, Uganda 14. Prof John Jerrim, UCL Institute of Education, University College London, UK 15. Prof Chris Bonell, Faculty of Public Health and Policy, LSHTM, UK. 16. Prof Janet Seeley, Faculty of Public Health and Policy, LSHTM, UK and MRC/UVRI and LSHTM Uganda Research Unit 17. Prof Helen A Weiss, MRC International Statistics and Epidemiology Group, LSHTM, UK On behalf of the MENISCUS Trial Group
Name and contact information for the trial sponsor {5b}	Research Governance and Integrity Office London School of Hygiene & Tropical Medicine Keppel Street London WC1E 7HT Tel: +44 207 927 2626 Email: rgio@lshtm.ac.uk
Role of sponsor {5c}	The sponsor and funder have no role nor authority in the study process or dissemination of the project

## Introduction

### Background and rationale {6a}

Menstrual health (MH) is defined as complete physical, mental, and social well-being in relation to the menstrual cycle [1]. Poor MH is an increasingly recognised public health issue [2, 3], and improving MH is essential to meeting the Sustainable Development Goals for gender equality, good health, quality education, sustainable water and sanitation, and related human rights [4]. Challenges to achieving MH among girls include inadequate puberty education and knowledge, lack of social support from teachers and peers, and insufficient access to appropriate products and water, sanitation, and hygiene (WASH) infrastructure [5]. These psychosocial and physical challenges to menstrual health impact on girls’ ability to succeed and thrive mentally and physically within the school environment and beyond [6, 7]. Menstrual health may also impact on educational outcomes but few intervention studies have evaluated this [8]. Improving secondary education for girls can potentially lead to sustained, long-term benefits to education [8], health (mental, reproductive, sexual) [9], productivity [2], and the environment [10] through multiple pathways (e.g. earnings, standard of living, child marriage and early childbearing, health, nutrition, well-being, agency and decision-making, and social capital).

In Uganda, it is estimated that only 35% of women aged 15–49 have the physical and social environment needed to manage their menstruation [11]. The Ugandan Government has the political will to improve MH [12], for example by forming a National Menstrual Hygiene Management (MHM) Steering Committee, holding the first international MHM conference in 2014 and celebrating International MHM Day each year. In 2015, the Ugandan Ministry of Education and Sports (MoES) published a Circular on Provision of Menstrual Hygiene Management (MHM) facilities to all primary and secondary schools ((Circular No. 1/2015).1/2015). This Circular recommends provision of clean, private, toilet facilities; regular supply of water and soap; emergency supplies of pads and painkillers; training of teachers, health assistants, and inspectors; and involvement of parents in supporting and providing MH information and materials. However, a recent review found that although most schools (*n* = 98; 94%) received this Circular, only 23 (30%) acted upon it [13].

A recent systematic review [5] showed that MH research has focused largely on describing the extent of poor MH with relatively few randomised controlled trials (RCTs) evaluating interventions which address common antecedents of menstrual experience, including knowledge, social support, restrictive behavioural expectations and the physical environment. A mid-term review of the UNICEF/Columbia University’s “MHM in Ten” initiative to advance the MH agenda in schools by 2024 recently highlighted a need to understand how school-based MH interventions are delivered and scaled [14].

This paper describes the protocol for the MENISCUS trial, a school-based cluster-randomised trial evaluating the impact of a school-based MH intervention on the education, health, and well-being of secondary school girls in Uganda. The MENISCUS trial will address evidence gaps on MH intervention effectiveness [5], process evaluation [4], and policy guidance. The intervention, grounded in social cognitive theory, was designed and piloted following formative research in Wakiso District in 2016 which showed that poor MH is a key factor associated with girls missing secondary school and affects their well-being [15]. The formative research found (i) an unmet need for effective interventions to enable girls to better manage both the psychosocial (anxiety, stigma, and distress) and physical aspects (pain management, use of appropriate materials to prevent leakage of menstrual blood, WASH facilities) of menstruation; (ii) the importance of including boys and teachers in a school-based MH intervention; and (iii) that self-collection of vaginal swabs for testing for bacterial vaginosis and vaginal yeast was acceptable and feasible in this setting [16]. The intervention was piloted in two schools in Wakiso District in 2017–2018 and found to be feasible to deliver and highly acceptable to stakeholders. The findings further suggested that the MENISCUS intervention may improve girls’ education and health in the Wakiso District school context [17, 18]. The aim of the current trial is to rigorously evaluate the impact of the intervention on education, health, and well-being outcomes among girls in 60 secondary schools in Uganda.

A completed SPIRIT checklist is available as a supplement (Additional file 1).

### Objectives {7}

We hypothesise that improved educational attainment, mental health outcomes, and related health- and well-being outcomes will be achieved by improving self-efficacy for MH in Ugandan secondary schools, through improving the MH social and physical environment, and supporting girl’s behavioural capability and observational learning.

The trial objectives are:

1. To evaluate whether the MENISCUS intervention improves educational attainment based on the Mathematics, English, and Biology curriculum taught during the intervention delivery and reduces mental health problems (primary outcomes) among secondary school girls in Uganda
2. To evaluate whether the MENISCUS intervention improves the following secondary outcomes:

i) Knowledge of puberty and menstruation; attitudes towards menstruation (girls and boys)
ii) Menstrual practices at the last menstrual period (LMP)
iii) Knowledge and practice of pain management during LMP
iv) Self-efficacy in addressing menstrual needs at LMP
v) Quality of life and subjective well-being (happiness and life satisfaction)
vi) Prevalence of urogenital infections (bacterial vaginosis, vaginal yeast, and urinary tract infections (UTI))
vii) School and class absence during menses (in a random subsample of girls)
viii) School and class absence overall (in a random subsample of girls)
ix) Self-confidence in mathematics and in science abilities, respectively

3. To conduct a process evaluation to assess whether the intervention was implemented with fidelity, and to understand the contextual factors affecting the implementation, the acceptability to participants and the intervention mechanisms
4. To evaluate the costs of setting up and running the intervention package, the unit cost per female student reached and the incremental cost-effectiveness of the intervention per unit increase in selected policy-relevant outcomes, relative to optimised usual care
5. To assess the policy environment around menstrual health in Uganda, focusing on how implementing the intervention contributes to, and aligns with, the attainment of the Government policy objectives on MH management in schools

### Trial design {8}

This is a parallel-arm, cluster-randomised control trial in 60 schools (clusters) with a 1:1 allocation ratio of clusters randomised to immediate delivery of the intervention or optimised usual care, with a mixed-methods process evaluation, and economic and policy analyses. Schools randomised to the optimised usual care arm will be offered the intervention at endline (approximately 1 year after randomisation).

## Methods: participants, interventions, and outcomes

We used the SPIRIT reporting guidelines in writing this protocol paper [19].

### Study setting {9}

The trial will be conducted in secondary schools in Wakiso and Kalungu districts in the Central region of Uganda. Wakiso District has a total area of 2704 km^2^ and includes both urban and rural areas. It partly encircles Kampala (Uganda’s capital city). According to the census in 2014, the population was estimated at 2 million, of whom 339,137 (17.3%) were aged 10–17 years and 48.9% of the 13–18-year-olds are estimated to attend secondary school [20]. Kalungu District is largely rural with an estimated population of 183,232 in 2014, of whom an estimated 40,960 (23%) were aged 10–17 years, with 36% of 13–18-year-olds attending secondary school [21].

### Eligibility criteria {10}

#### Schools

##### Inclusion criteria for schools

Mixed-sex secondary schools with S1–S4 classes; day or mixed day/boarding schools; at least minimal WASH facilities (including an improved water source and sex-specific sanitation facilities that are functional, usable, and accessible to female students at the time of the rapid assessment eligibility survey) [22], estimated enrolment of approximately 50–125 female S1 students as of January 2020 (before COVID-19 lockdown) in Wakiso and 40–125 female students in S1 in Kalungu, based on the 2019 report on the Master List of Education Institutions in Uganda [23].

##### Exclusion criteria for schools

Schools that are currently participating in a menstrual health-related programme; boarding schools with no day students; single-sex schools and schools exclusively for students with disabilities.

### Trial participants

#### Inclusion criteria for trial participants

(i) All female students in S2 in 2022 who are present at the time of the baseline survey, whose parent/guardian provided consent (if aged <18 years) and who have assented/consented to the trial and intervention procedures, and (ii) a simple random sample of 15 male students (in S2 in 2022) per school, present at the time of the baseline survey who have consented to the trial procedures. Furthermore, all female students present at the time of the endline survey in 2023 with consent/assent for the research will be eligible for the outcome assessment, as will the male students with baseline data.

#### Inclusion criteria for the nested sub-study

A simple random sample of 25 female students in each school will be randomly selected from those enrolled in the trial, to participate in a daily diary sub-study.

### Who will take informed consent? {26a}

#### Informed consent procedures

Trained research team members will seek (i) written informed school-level consent from head teachers or directors to cover school-level research and intervention activities and (ii) written informed parent/guardian consent for female students obtained in person where possible, through information meetings held at each school. Parents/guardians who were unable to attend information meetings at school were reached through home follow-up visits or through phone calls to seek documented verbal informed consent. The consent forms include procedures for the self-collection of two vaginal swabs, one of which will be stored and will be shipped overseas, and for all anonymised data collected to be shared with other researchers. A separate consent form was administered for the menstrual cup provision, so parents/girls could opt to participate in all trial activities except for receiving a cup. The ethics committees agreed a waiver for parent/guardian consent for boys, as their participation in the (knowledge-focused) boys’ survey was deemed to carry minimal risk.

For student assent, information videos explaining trial procedures will be available for girls whose parent/guardian consent to the trial either including or excluding the menstrual cup, respectively. Male participants will be shown a video describing relevant procedures for boys. All three videos will be available in English and Luganda. Students will be asked by a trained research assistant to watch the relevant video on a tablet with headphones, they will also answer a few questions at the end of the video to assess their understanding of the trial and if they agree, they will provide secure electronic (e-) assent by signing on the tablet. Participants are given a paper copy of the assent form to retain.

Participants selected for qualitative interviews will be asked for parent/guardian consent and student assent prior to taking part in interviews (except for female students who already consented to the main trial that includes consent for qualitative interviews). Additional parent/guardian consent (for girls) and student assent (for all students) will occur prior to the endline survey for students who were not present at baseline.

### Additional consent provisions for collection and use of participant data and biological specimens {26b}

Consent and assent were collected for collection and use of participant data and biological specimens (vaginal swabs and urine samples) as part of the main trial consent procedures.

### Interventions

#### ***Explanation for the choice of comparators*** {6b}

The choice of comparator was chosen to align with Government MH guidelines and is optimised usual care. Optimised usual care will include (i) a copy of the Government Circular on Provision of MHM for primary and secondary schools ((Circular No. 1/2015).1/2015) [13], (ii) the 2018 National Sexuality Education Framework for each school [24] and (iii) copies of the Government Menstruation Management Reader to distribute to each girl and boy in S2. The Reader was written for girls and boys in primary schools to help them understand what menstruation is, why it happens, how to manage it and who to talk to about it. Schools randomised to the comparator arm will be able to receive the MENISCUS intervention after the endline survey.

#### Intervention description {11a}

The schools randomised to the intervention arm will receive the optimised usual care plus the MENISCUS intervention which includes puberty education, a drama skit, provision of a menstrual health kit and training, pain management, WASH improvements, and creation of a Menstrual Health Action Group (AG). Details of the MENISCUS intervention are given in Table 1, reported according to the TIDieR framework.

**Table 1:** MENISCUS intervention reported according to the TIDieR framework

**MENISCUS intervention components**	
1. Menstrual Health (MH) Action Group	
2. Puberty education	
3. Drama skit	
4. Menstrual health kit and training	
5. Pain management	
6. Water, sanitation, and hygiene (WASH) improvements	
**Why: rationale for each component**	
1. To facilitate school-ownership of the MENISCUS intervention and hence that it is implemented well in the school	
2. To strengthen schools’ capacity to deliver knowledge of puberty and menstruation	
3. To enable a supportive school environment and reduce stigma and teasing	
4. To improve menstrual management and provide product choice	
5. To improve ability to manage menstrual pain	
6. To improve menstrual hygiene management	
**What: materials and procedures**	
1. The WoMena staff training the Action Group will receive a manual and training to deliver the training. The MH Action Group members will receive in-person training in puberty and menstruation and on running an MH Action Group. The MH Action Group members will receive a T-shirt with the MENISCUS logo, the budget for running the Group, an MH kit and an MH Action Group Charter, guide and action plan to complete. The MH Action Group will receive support by WoMena Uganda after the initial training in follow-up sessions.	
2. The schools’ staff delivering puberty education will receive in-person training on how to deliver the Ministry of Education and Sports’ (MoES) training on menstrual health management and a copy of the MoES Training Manual for teachers and other stakeholders on Menstrual Health Management.	
3. The drama group members (facilitators and students) will receive two facilitation sessions on menstruation and the drama skit. They will receive an outline of a MH-related drama to be developed into a drama skit and performed at an existing school meeting. A small budget, managed by the MH Action Group, will be available to buy props and supporting materials for the performance and the students taking part. The students taking part will receive a T-shirt with a MENISCUS logo.	
4. i) Trained school members (“MENISCUS trainers”) including student leaders and prefects to provide peer support to girls will receive in-person training on how to deliver menstruation education sessions (a joint session of menstruation for boys and girls and separate girls’ and boys’ sessions), alongside receiving specially developed materials to deliver these sessions, flipcharts and a training manual. The trainers will receive a certificate, a T-shirt, and an MH kit. ii) S2 students will receive in-person education sessions delivered by the MENISCUS trainers. The students will receive a MENISCUS booklet developed by WoMena to accompany the education sessions. The girls’ session includes puberty, how to use the MH kit components, and tracking and managing their periods, including managing menstrual pain. Students will receive a MH kit consisting of the AFRIpads “schoolgirl kit” of 5 reusable pads, a towel, soap, two pairs of underwear, and MH booklet with a menstrual tracker, plus a menstrual cup and container for girls who consented to the cup. The boys’ session will include male puberty, genital hygiene, male circumcision, and attitudes towards girls who are menstruating. iii) Female caregivers will receive in-person education around menstruation and the use and care of reusable menstrual products (menstrual cup and reusable pads). Caregivers will receive a MH kit of the AFRIpads “standard kit” of 6 reusable pads, a towel, soap, and underwear plus a menstrual cup and container to those who consent and are willing to receive the cup.	
5. S2 female students will receive (i) an information sheet about safe use of paracetamol and ibuprofen, plus information on pain management methods more broadly in the MENISCUS MH booklet, and (ii) vouchers to be redeemed for a maximum of 6 tablets per month from the school nurse or designated senior teacher who will be trained in administering these by the MENISCUS clinical officer.	
6. Basic improvements to school WASH facilities (installation of locks, repair of broken doors, provision of bins and toilet paper holders fixed to the wall, liquid hand washing soap, and water drums).	
**Who provided**	
1. The MH Action Group will consist of 6–8 people selected by school management and will be responsible for implementing and maintaining the intervention. They will be MH champions within the school. The MH Action Group members will include a minimum of at least one representative of school management, a senior woman teacher, a student, and a parent. The group will receive: • 1-day training on menstruation and being a member of the MH Action Group by WoMena Uganda • Follow-up and support from WoMena Uganda facilitators at 3 MH Action Group meetings	
2. Up to 5 male and female teachers per school who usually deliver puberty education to secondary students will attend a 2-day training, followed by 1-day training by WoMena Uganda.	
3. The drama skit component will involve the drama group in the school, including the drama teacher and students who attend. If there is no active drama club or group in the school, the MH Action Group will be encouraged to support the creation of a group. Drama groups, including a drama teacher/facilitator and around 30 students in the school, will be invited to 2 facilitation sessions which contain training on menstruation and puberty and introductions to the concept of the drama skit and the script. The group will also have rehearsals observed and supported by WoMena.	
4. Approximately 7 school staff and students will be selected as MENISCUS trainers, responsible for training S2 students in MH and using the MH kit. Selected prefects and student leaders will support the trained staff in training activities and act as peer support in their schools. Training participants will be selected by school management according to specified selection criteria (for example, motivation and agreement to attend trainings and train young people of puberty, menstruation and using reusable menstrual products, as well as having the trust and respect from students). Approximately 8 female caregivers associated with the schools will be selected by school management according to a specified criterion (willing to try products, respected in the community, has a child who attends the school).	
5. The school nurse or other designated senior teacher will be trained on safe use and management of paracetamol and ibuprofen by the MENISCUS clinical officer, including how the voucher scheme works.	
6. WASH improvements will be made by a contractor employed by MRC/UVRI and LSHTM Uganda Research Unit. The MH Action Group members will be responsible for maintaining the improved WASH facilities.	
**How — modes of delivery**	
1. WoMena Uganda will provide face-to-face group training around the MH Action Group and will have in-person follow-up visits for each school to observe and support.	
2. WoMena Uganda will provide face-to-face group training in puberty education to teachers.	
3. WoMena Uganda will introduce the drama skit and provide a brief training on menstruation in person and will attend a rehearsal to give feedback and support in each school.	
4. WoMena Uganda will provide face-to-face group training of MENISCUS Trainers, with follow-up in each school to provide support. WoMena Uganda will provide face-to-face group trainings of female caregivers.	
5. The MENISCUS clinical officer will provide training of school nurses or other designated staff on appropriate analgesic use, and WoMena will provide training on alternative pain management strategies to MENISCUS Trainers.	
6. WASH improvements will be made at each school by the contractor.	
**Where**	
All intervention elements will take place at the school or at an event space in the community for the trainings.	
**When and how much**	
All elements will be delivered over the course of a year, following randomisation.	
1. The training of the MH Action Groups will take place once, in clusters of about 5 intervention schools based on their location. The training will be for 1 day per cluster of schools. The MH Action Groups following this will run for 8–11 months (depending on when they receive their training). They will have 3 meetings, with one attended by the WoMena Uganda team over this period.	
2. The training of the puberty educators will be two sessions in each district. The first training will take place approximately 4 weeks ahead of the second training. The first training will last 2 days and the second training will last 1 day. Those trained will then deliver the puberty education to students they teach over the following 8 months.	
3. Two drama skit facilitation sessions will take place in each school followed by an attendance at one rehearsal. The drama skit facilitation sessions, rehearsals, and performance will take place over a period of 3 months.	
4. The training of MENISCUS trainers will take place once with two separate sessions firstly, a 2-day session and a 1-day follow-up session, approximately 1 month after the initial training session. The MENISCUS trainers will then deliver this training to students at the school over the following 3 months. The training of female caregivers will take place once in a 1-day session.	
**Tailoring**	
There are plans for adaptations and tailoring based on attendance at the initial trainings and the delivery of the training by the MENISCUS trainers to the S2 students. If there is no attendance at the initial trainings, schools will be offered these trainings at their schools individually unless there are enough schools who did not attend and then another training will be held in a central location. If schools do not deliver the training to S2 students, they will be supported to do so by WoMena Uganda by either delivering the sessions or being present when the school delivers all the sessions.	
**How well**	
This will be evaluated in the process evaluation (please see details in Table 4)	

The MENISCUS intervention is grounded in social cognitive theory which suggests that individuals learn by observing others [25]. The theory suggests that the following factors reinforce behaviour change: increased social support for and instilling expectations of behaviour change, increased self-efficacy, and using observational learning. During our formative work, we developed a logic model of change with stakeholders. Aligning this logic model with the core constructs of social cognitive theory, we developed a theoretical framework for the intervention which asserts that the intervention (i) increases girls’ self-efficacy to manage their menstruation (e.g. through provision of an MH kit and pain management options), (ii) positively reinforces girls’ learning (and also that of boys, teachers, and parents) to create a more supportive MH environment (e.g. through drama) and (iii) reinforces behaviour change through positive reinforcement and expectations (e.g. through improving WASH facilities and reduced teasing) [18].

#### Criteria for discontinuing or modifying allocated interventions {11b}

Adverse events (including serious adverse events) will be logged and reviewed by the trial clinical officer and the independent Data Monitoring and Ethics Committee if necessary, as described in the “Adverse event reporting and harms {22}” section. If these are deemed to be related to aspects of the intervention (most likely to analgesic use or the menstrual cup), the relevant intervention component (painkillers or cup) will be withdrawn from the participant.

#### Strategies to improve adherence to interventions protocols, and any procedures for monitoring adherence {11c}

Based on our pilot study, we anticipate high levels of uptake of most of the individual and behavioural components (drama skit, MH kit, and pain management); however, we saw lower adherence to delivery of puberty training by the teachers and the WASH component [17]. To improve overall school-level adherence (the WASH component, delivery of puberty education and drama skit), Menstrual Health Action Groups (MH AG) at each school will be tasked with ensuring that each element of the intervention is delivered, maintained, and sustained. The MH AGs will also engage schools to implement the training of trainers’ model which allows schools to deliver the trainings in a way that suits their school schedules. We will evaluate school-level adherence and individual-level exposure data as part of the process evaluation and in the endline survey. The implementing partner will record attendance at trainings and schools will also be asked to track and report their activities. This information will be triangulated with observations conducted as part of the process evaluation.

#### Interventions: concomitant care {11d}

The MENISCUS intervention will not require alteration to usual care pathways (including the use of any medication).

### Provisions for post-trial care {30}

The trial sponsor carries Clinical Trial/Non-Negligent Harm Insurance and Medical Malpractice Insurance.

We do not expect serious adverse events due to our intervention, but in case of referrals due to SAE/SAR, we will make clinical care payments or reimburse the participants on the bills paid as stipulated in the private health facility signed Memorandum of Understanding. For suspected cases of toxic shock syndrome (TSS) due to the use of the menstrual cup, immediate referral for admission will be made to the designated district or national referral health facilities. We will pay for transfer to the hospital as needed for trial participants.

### Outcomes {12}

The primary outcomes are educational attainment and mental health problems among girls present at endline, which is approximately 12 months after randomisation. Secondary outcomes are shown in Table 2.

**Table 2:** Primary and secondary impact outcome measures

Outcome	Measure	Tool	Study population
1) Educational attainment in girls (primary outcome)	Mean total score on examination of curricula material in English, Mathematics, and Biology taught during the intervention year (adjusted for baseline score)	Examination set by UNEB	All girls in the endline survey
2) Mental health problems (primary outcome)	Mean Total Difficulties score from the Strengths and Difficulties questionnaire (adjusted for baseline SDQ score)	Strengths and Difficulties 25-item questionnaire	
i) Knowledge of puberty and menstruation; attitudes towards menstruation	Proportion answering all knowledge questions correctly Proportion answering all questions on myths correctly Proportion with “good” responses on attitudes	Endline survey	All girls and a random sample of boys in the endline survey
ii) Menstrual practices at last menstrual period (LMP)	Proportion using manufactured methods only at LMP Proportion correctly washing and drying reusable pads and/or menstrual cups at LMP Proportion with ‘adequate MHM at school’ at LMP^a^ Mean score on the Menstrual Practice Needs Scale	Endline survey	All girls in the endline survey
iii) Knowledge and practices of pain management at LMP	Proportion knowing 4 or more effective pain management methods^b^ Proportion of those with pain at LMP who used an effective pain management method		
iv) Self-efficacy of MH	Mean score on Self-efficacy in Addressing Menstrual Needs Scale [26]		
v) Quality of life and happiness	Mean CHU9D score (9-dimension questionnaire) Self-reported measure of happiness		
vi) Prevalence of urogenital infections	Prevalence of bacterial vaginosis (Nugent score >7) Prevalence of vaginal yeast (Gram stain) Prevalence of UTI (symptoms ± leucocyte esterase and/or nitrates with urine dipstick)	2 vaginal swabs^c^ Urine Multistix 8 dipstick if symptomatic for UTI	Post-menarchal girls only (~95%) at endline
vii) School and class absence during menses	Proportion of full school days missed by girls during their period (adjusted for absence on non-period days) Proportion of school days with classes missed by girls during their period (adjusted for absence on non-period days)	Daily diary on school attendance and menstrual cycle, administered for 12 weeks at endline	Random subsample of ~1500 post-menarchal girls at endline
viii) School and class attendance overall	Proportion of full school days missed Proportion of school days with classes missed		
ix) Self-confidence in Mathematics and science abilities	Mean score on the Students Confident in Mathematics scale^d^ Mean score on the Students Confident in Science scale^d^	Endline survey	All girls in the endline survey

^a^Self-reported use of clean materials to absorb/collect blood, changed privately, safely, hygienically, and as often as needed

^b^Use of painkiller, drinking water, using water bottle, exercise, stretching, and foods with lots of water

^c^The second swab will be stored in RNA*later*^TM^ Stabilization Solution for future testing to characterise the vaginal microbiome by 16S rRNA sequencing (funds not included)

^d^Standardised scales from the IEA’s Trends in International Mathematics and Science Study – TIMSS 2011 Copyright © 2012 International Association for the Evaluation of Educational Achievement (IEA). Publisher: TIMSS & PIRLS International Study Center, Lynch School of Education, Boston College

*Educational attainment* will be assessed through bespoke pre-tested assessments administered and marked by the Uganda National Examination Examinations Board (UNEB). The baseline assessment will test Mathematics and Biology material taught to S1 students pre-intervention (2020–2021), and the endline assessment will assess recently taught Mathematics, Biology, and English material. UNEB assessors will be masked to the trial arm. The outcome will be the composite examination score at endline. The rationale for the subject choice is that the Biology curriculum taught in S2 includes puberty knowledge in the reproduction topic and is directly related to our intervention and that English and Mathematics are core subjects recognised by policymakers as influential for scale-up.

Mental health problems will be assessed for all girls at baseline and endline using the Total Difficulties Scale of the Strengths and Difficulties Questionnaire (SDQ-25). This includes emotional symptoms, attention, and peer-relationship problems. The outcome will be total SDQ-25 score, analysed as the examination data above. The SDQ-25 has been validated among adolescents in sub-Saharan Africa including in Uganda [27].

### Participant timeline {13}

**Fig. 1:**
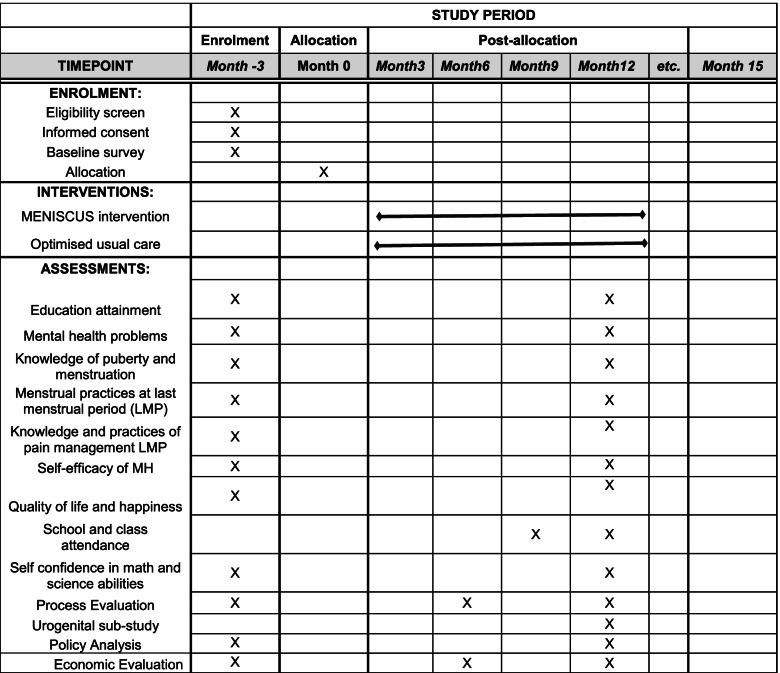
Time schedule of enrolment, interventions, and assessments

Different items will be measured according to the point in time when the data are collected. The enrollment into the study is over 3 months, and assessments for the primary and secondary outcomes will take place at different time points up to 12 months, with the majority of the assessments at both baseline and endline (Fig. 1).

### Sample size {14}

The sample size of 60 schools is based on an estimated harmonic mean of 60 female students per school at endline. This sample size provides 84% power to detect a standardised mean difference of 0.2 for continuous outcomes, assuming an intra-cluster correlation (*ρ*) of 0.05 and type 1 error of 0.05. The effect size of 0.2 is that observed for the primary outcome of SDQ score for girls in the pilot [17] and is considered moderate for educational outcomes and hence of policy relevance. This sample size provides 90% power to detect 12% vs 20% participants answering all knowledge questions correctly in the control vs intervention arms for girls (assuming *ρ* = 0.05) and 85% power to detect 4% vs 12% for boys (as in the pilot), assuming 10 boys per school are seen at endline. We have not adjusted the type 1 error because the two primary outcomes are independent, and an improvement in one without the other will be informative. From our pilot, we expect >95% of girls to be menstruating at endline, and this is accounted for in the power calculation where relevant.

The subsample of 1500 girls selected to provide daily diary data on school/class attendance and their menstrual cycle over 12 weeks (60 school days) at endline provides >98% power to detect a 20% reduction in period-related absence at endline based on pilot data [17] and ~89% power to detect this difference by district or other equally sized subgroups.

### Recruitment {15}

Schools were selected and assessed for eligibility in two stages. First, an initial desk review was conducted using the 2019 Master List of Education Institutions. Of 631 secondary schools listed in the two districts (583 in Wakiso and 48 in Kalungu), 160 schools (138 in Wakiso, 22 in Kalungu) were deemed potentially eligible based on the listed information (i.e. mixed-sex, not exclusively boarding, and 40–125 (Kalungu) or 50–125 (Wakiso) female students registered in S1). The remaining schools were stratified by district and Government/private ownership and randomly ordered.

The second stage was a rapid assessment which consisted of stakeholder workshop, phone calls, school visits, and interviews to confirm eligibility and assess the schools’ physical and social environments for MH, in preparation for the trial. Of 110 head teachers of schools contacted, 84 were deemed potentially eligible for the trial from an initial screening phone call and 75 (89% of those potentially eligible) were invited to participate in the rapid assessment. Following the rapid assessment, 67 schools were confirmed eligible and 58 (87%) agreed to participate. Head teachers of these schools and district officials were invited to a second stakeholder workshop in each district in October 2021, where results of the rapid assessment were discussed and schools were asked to sign a Memorandum of Understanding (MOU) and provide a list of female students due to start S2 in 2022 if they agreed to participate in the trial. We assessed a further two schools (to replace two that declined to participate) from the original list for eligibility in February 2022 to achieve the final sample size of 60 trial schools (16 in Kalungu District and 44 in Wakiso District).

### Assignment of interventions: allocation

#### Sequence generation {16a}

The 60 trial schools will be allocated using 1:1 restricted randomisation. Within each district, we will stratify schools by median baseline examination score (high/low) and will use covariate-constrained randomisation to minimise imbalance with respect to key factors (mean baseline examination score, past school examination scores, mean baseline SDQ score, school type (Government vs. private), mean score on the Menstrual Practice Needs Scale, and the number of S2 female students). A list of 1000 eligible allocations will be randomly selected from those meeting the specified balance criteria and will be sent to the data manager by the trial statistician.

#### Concealment mechanism {16b}

We will minimise biased recruitment by conducting the randomisation after the baseline survey [28] and conducting semi-public randomisation ceremonies to select the allocation.

#### Implementation {16c}

Semi-public randomisation ceremonies will be held in each district following completion of the baseline survey, in the presence of representatives from schools, the District Education Officer (DEO), the MoES, and the MoH. At these ceremonies, one allocation will be randomly selected from the list of 1000 allocations as follows: Three representatives will each select one ball from an opaque bag to form a 3-digit number corresponding to the selected allocation sequence. A fourth ball will be selected, to decide which schools are in the intervention arm (even digit) and which are in the control arm (odd digit).

### Assignment of interventions: blinding

#### Who will be blinded {17a}

The primary outcome of the educational assessment will be masked as it will be independently administered by UNEB with no knowledge of arm allocation. We will minimise bias in other outcome assessments by having separate teams for implementation (WoMena Uganda) and outcome assessment (MRC/UVRI and LSHTM Uganda Research Unit). Masking of participants, school staff, and implementers is not possible as it will be clear whether the intervention is being implemented. Laboratory technicians testing self-collected swabs for bacterial vaginosis, vaginal yeast, and UTI will be masked. All outcome data will be anonymised, analysed, and interpreted masked to the trial arm. The clinical officer will not be masked to arm as they will be training staff in the intervention schools and will be responsible for assessing causality of adverse events.

#### Procedure for unblinding if needed {17b}

The independent Data Monitoring and Ethics Committee will advise if the principal investigator needs to be unblinded due to safety concerns.

### Data collection and management

#### Plans for assessment and collection of outcomes {18a}

Prior to randomisation, the research team will be trained on collecting baseline survey data, which will be conducted in all the 60 schools before randomisation. This will include qualitative process evaluation baseline data. After randomisation, the intervention will be implemented by WoMena with ongoing process evaluation data collection. The endline survey will take place approximately 1 year after randomisation. Details of outcome measures and tools used are given in Table 2.

#### Plans to promote participant retention and complete follow-up {18b}

The trial is powered to compare outcomes between participants in the two randomised groups at endline, adjusting for school-level baseline measures of outcome variables. There may be differential loss-to-follow-up by arm, if, for example, the intervention reduces school dropout. We will collect data on school dropout in girls and adjust for the proportion dropout in endline analyses.

### Data management {19}

Baseline and endline survey data will be self-collected by participants on tablets using ODK Collect software and uploaded to the MRC/UVRI and LSHTM Uganda Research Unit secure ODK Central server. Other quantitative data (daily diaries, laboratory forms, and analgesic vouchers) will be collected on paper case report forms (CRFs) and entered into a REDCap database. The data manager will regularly monitor data quality and any data queries will be resolved in collaboration with the relevant field team member or data entrant. In-depth interviews and focus group discussions will be audio-recorded and transcribed verbatim, including relevant non-verbal communications, and translated into English by the study interviewers for those conducted in Luganda. Cost data will be inputted into an Excel-based costing tool.

In the endline survey, female participants will be asked a set of additional questions on current genital symptoms. After completing the survey, trained study staff will explain and demonstrate the procedure for obtaining the vaginal swab. These will be tested for bacterial vaginosis and vaginal yeast. After the student has watched the video and asked any questions, the study staff will pass the student the 2 swabs for self-collection in a private setting. The swabs will be returned to the staff member. Participants reporting urogenital symptoms will be referred to an appropriate clinic for syndromic management and treatment as per Ugandan Clinical Guidelines. Those with symptoms of a UTI (e.g. urinary burning and frequency) will have their urine tested by Multistix 8 dipstick to look for signs (e.g. nitrite and leucocyte esterase positivity) to aid diagnosis of a UTI as per Ugandan Clinical Guidelines. The test result will be sent with the participant to the local clinic for management.

The process evaluation will use quantitative data from the endline survey supplemented with data from log books, structured observations, focus group discussions and in-depth interviews (Tables 3 and 4). This will enable us to understand how the intervention was implemented and received and the social, structural, and logistical factors that impede or facilitate this, as well as how staff and students were able to engage with it. We will also explore implementation processes and mechanisms of impact, and how these vary across the schools, using qualitative data. Data will be collected from all intervention schools via phone interviews with one senior staff member at baseline and endline and semi-structured interviews with one member of the school’s MH Action Group member purposively sampled to ensure overall variation across schools in gender and type of member (e.g. staff, student, other stakeholders) at mid-line and endline. More in-depth qualitative data will be collected from 4 purposively selected case study schools: group discussions with female students, male students and school staff at mid-line and endline and semi-structured interviews with staff and female students at mid-line and endline and with caregivers at endline.

**Table 3:** Overview of qualitative data collection activities

Tool	Participants	When
Semi-structured phone interviews with senior school staff	60 senior staff members (one per school, *N*=60)	Baseline and endline
Semi-structured interviews with MH Action Group members	30 members of the school’s MHM leadership group member (one per intervention school *N*=30) (e.g. staff, student, other stakeholder)	After initial intervention delivery and endline
Semi-structured interviews with intervention providers	8 WoMena Uganda staff	After initial intervention delivery and endline
**In case study schools (*N*=4)**		
Focus groups	4 FGs with 4 female students (*N* = 16) 4 FGs with 4 school staff (*N* = 16) 4 FGs with 4 male students (*N* = 16)	After initial intervention delivery and endline
Semi-structured interviews with school staff and female students	12 staff members and female students (menstrual cup recipients and non-recipients)	After initial intervention delivery and endline
Semi-structured interviews with female caregivers	8 female caregiver MH kit recipients	Endline

**Table 4:** Process evaluation research questions and data collection methods and sources

Research domain		Research questions	Data sources
**Implementation** *What is implemented, to who, and how acceptable is it?*	**Fidelity**	Was training and school components implemented as planned? What adaptations were made? How did fidelity vary between schools? What were the barriers and facilitators to implementation fidelity?	Log books Structured observations Endline surveys with male and female students IDIs and/or FGDs with students, staff, MH Action Group members, intervention deliverers WASH checklist
	**Reach and acceptability**	What proportion of students received and accessed various components of the intervention? How acceptable was the intervention to students? How did reach and acceptability vary by student characteristics? What contextual factors affected reach and acceptability?	
**Usual treatment**		What is usual provision of puberty lessons and MH activities in control schools? What puberty and MH activities are happening within schools and in the wider community? What WASH provision exists in control schools?	Baseline/endline surveys with male and female students Rapid assessment data Structured interview with senior staff member at endline WASH checklist
**Mechanism of impact** *How does intervention lead to change?*		How did intervention providers and school staff and students describe how they used the intervention resources to enact the intervention, including any adaptations? What mechanisms did participants say were triggered by enactment of intervention activities and what impacts and other consequences did these have on students and the school?	IDIs and/or FGDs with students, staff, MH Action Group members, intervention deliverers Structured interview with senior staff member at endline
**Context** *How does context affect implementation and outcomes?*		How did participants describe enactment and mechanisms as affected by school context? What were contextual reasons for adaptations to the intervention and its delivery?	IDIs and/or FGDs with students, staff, MH Action Group members, intervention deliverers Rapid assessment data Structured interview with senior staff member at endline Log books

The economic evaluation will evaluate the costs of setting up and running the intervention package, the unit cost per female student reached, and the incremental cost-effectiveness of the intervention per unit increase in selected policy-relevant outcomes, relative to optimised usual care. The cost-effectiveness measures will be compared to similar school-based interventions in the region to inform scalability and financial sustainability. The policy analysis will assess the policy environment and financial sustainability of our intervention in line with Government policies and frameworks for MH and sexual and reproductive health.

### Confidentiality {27}

Identifiable data collected will be stored securely and their confidentiality protected in accordance with the Data Protection Act 1998. The risk of disclosure will be minimised by training staff on the need for confidentiality (including training on Research Ethics), secure storage of documents, and use of linked data with a unique participant ID instead of names or other personal identifiers. All information regarding the participants will remain confidential to the extent allowed by law. Screening forms and case report forms will be kept in a secured location with access limited to authorised study staff. Consent and assent forms will be stored separately in a locked cabinet as will all other forms, logbooks, and appointment books that link participant ID numbers to other identifying information.

### Plans for collection, laboratory evaluation, and storage of biological specimens {33}

The self-collected vaginal swabs will be transported to the MRC/UVRI laboratory in Entebbe for processing and storage. Upon receipt, one swab will be Gram stained and Nugent scored [29]. The second swab will be stored immediately in a cool box before being transferred to a −80C freezer within 24 h for storage and potential future testing related to vaginal microbiota. Participants with symptoms of a UTI (e.g. urinary burning and frequency) will have their urine tested by Multistix 8 dipstick to look for signs (e.g. nitrite and leucocyte esterase positivity) to aid diagnosis of a UTI as per Ugandan Clinical Guidelines. The test result will be sent with the participant to the local clinic for management.

### Statistical methods

#### Statistical methods for primary and secondary outcomes {20a}

We will follow CONSORT guidelines for the analysis of cluster-randomised trials [30]. A Statistical Analysis Plan will be finalised prior to data unblinding and analysis. A participant flow diagram will be completed, following CONSORT guidelines for CRTs. Descriptive statistics for school-level and individual-level baseline characteristics will be shown by arm and will include mean and SD (or median and range if non-normal) (for continuous variables) and counts and proportions (for categorical variables). Factors showing substantial baseline imbalance by arm (agreed in advance of unmasking by the co-investigators) will be noted for inclusion in the effectiveness analysis. The primary and secondary outcome analyses (Table 2) will be carried out for all schools as randomised (intention-to-treat). Intervention effects will be estimated using individual-level regression analysis, adjusted for clustering by school and for baseline measures of outcome variables, restriction variables and factors imbalanced by arm at baseline. Results will be presented as standardised mean difference (continuous) or odds/prevalence ratio (binary) with a 95% confidence interval. For the analyses of daily diary data, there are repeated measures per girl, and analyses will additionally adjust for within-girl clustering using random-effects. Sensitivity analyses will be conducted, including randomisation inference.

#### Interim analyses {21b}

We have no interim analyses or stopping guidelines as there are no anticipated problems with the majority of trial components that are detrimental to the participants.

#### Methods for additional analyses (e.g. subgroup analyses) {20b}

Effect modification will be assessed for a-priori-defined subgroups (e.g. district; Government vs private school; age group; baseline examination score; baseline WASH facilities). The cost-effectiveness analysis will include cost–utility analysis comparing costs and utilities of the two groups with costs considered from a provider perspective and health outcomes measured by QALYs based on the Child Health Utility 9D (CHU9D), cost-effectiveness analyses of the intervention effect on SDQ score, and cost–well-being analysis, with costs considered from a provider perspective and outcomes measured by improvement in happiness and life satisfaction scores. To assess bias due to attrition, we will compare reasons for students leaving school, and baseline characteristics associated with dropout, between arms.

#### Methods in analysis to handle protocol non-adherence and any statistical methods to handle missing data {20c}

We will use intention-to-treat methods for primary and secondary outcomes. The primary analysis includes all female trial participants present at endline and will adjust for relevant baseline measures. We therefore do not need to impute missing endline data.

#### Plans to give access to the full protocol, participant-level data, and statistical code {31c}

The full protocol will be made available on the study website (https://www.lshtm.ac.uk/research/centres-projects-groups/meniscus), and participant-level data and statistical code will be available through the LSHTM Data Repository (https://datacompass.lshtm.ac.uk/).

### Oversight and monitoring

#### Composition of the coordinating centre and trial steering committee {5d}

The trial sponsor, the London School of Hygiene and Tropical Medicine (LSHTM), will organise monitors to review the source documents as needed, to determine whether the data reported are complete and accurate. The Trial Steering Committee (TSC) is chaired by an independent scientist and consists of 6 further independent members (including Government representatives), 3 observers (including a funding representative), and 7 non-independent members. The Trial Management Group (TMG), chaired by the PI and attended by co-investigators, meet every 1–2 months to oversee trial progress and report to the TSC.

Our research will be communicated to the public and stakeholders in Uganda, the UK, and globally through the trial website and webinars. At the beginning of the trial, we conducted initial stakeholder workshops to introduce the trial to representatives from the Ministry of Education and sports, Ministry of Health representative, district, WoMena Uganda, and schools. At the end of the trial, we will visit each school to hold a facilitated discussion about the findings with the students, parents, and teachers and will offer the intervention to schools randomised to the control arm. We will conduct a stakeholder workshop including local and national stakeholders (school representatives, parents, community leaders, policymakers from the Ministries of Education and Health, NGOs, and other academic researchers) to discuss policy implications of the findings. We are working with the Community Advisory Boards that are composed of representatives of the community participating in the research (parents, adolescents, teachers). Their role is to provide advice on how best to conduct the informed consent process and the implementation of research protocols

#### Composition of the data monitoring committee, its role, and reporting structure {21a}

The Independent Data Monitoring and Ethics Committee (IDMEC) consists of 3 independent members who are knowledgeable in the subject area, safety monitoring, and clinical trials. One of the members is a statistician to provide independent statistical expertise, especially with regard to interpretation of accumulating data and guidance through the report. The role of IDMEC is to safeguard the interests of trial participants, monitor the main outcome measures, and monitor the overall conduct of the trial. The IDMEC reviews the trial’s progress including updated figures on recruitment, data quality, adherence to protocol treatment and follow-up, and main outcomes and safety data. The IDMEC shares their recommendations with the Trial Steering Committee through the trial statistician, usually within 2 weeks of the meeting. A copy of this will be stored at LSHTM.

#### Adverse event reporting and harms {22}

Prior to randomisation, the trial clinical officer will map and visit health centres III, IV, district hospitals, and referral hospitals in each district and meet with District Health Officers to confirm referral procedures. Health centres III are sub-county-level clinics led by a senior clinical officer, with a general outpatient clinic, maternity ward, and functioning laboratory. Health centres IV are county or parliamentary constituency-level mini-hospitals, led by a senior medical officer, with in-patient wards for men, women, and children, respectively, and a theatre for emergency operations. Hospitals provide comprehensive medical services, receive referrals from lower health facilities, are relatively equipped with advanced diagnostics, and have advanced capabilities for resuscitation and life support. As part of the intervention, girls will be provided with the toll-free number for Public Health Ambassadors Uganda (PHAU) toll-free number 0800-300-600 and the Marie Stopes Uganda (MSU) contact centre (phone: 0800 220 333; WhatsApp: 754001 503), which operates 7 days per week and is managed by a team of 12 qualified counsellors and 2 nurses to provide confidential advice on sexual and reproductive health and referral to services in Luganda and English.

School nurses or designated person in both arms will be trained to monitor and report all suspected serious adverse events (SAEs) (i.e. death, life-threatening event, hospitalisation, persistent or significant disability or incapacity). These will be assessed by the trial clinical officer and noted in the SAE reporting form, including determination of a possible relationship with the intervention (analgesic use or the menstrual cup). Participants with SAEs will be rapidly referred to previously mapped health centre IVs by the MENISCUS clinical officer or the school or health facility III as appropriate. Transportation to the hospital will be provided by the study if needed. The trial clinical officer will be responsible for initially assigning causality and SAEs will be reported to the trial manager and PI within 24 h, using an SAE form. SAEs that are at least possibly related to the study interventions will be reported to the IDMEC within 24 h of the PI becoming aware of it. If awaiting further details, a follow-up SAE report will be submitted promptly upon receipt of any outstanding information.

Non-serious adverse events will be reported in the intervention arm schools only, following training by the trial clinical officer. Each participant experiencing a non-serious adverse event in intervention arm schools will have a record of their adverse events archived confidentially at the MRC MENISCUS office.

Girls who self-report urogenital symptoms during the endline survey will be referred to a local clinic (both private and government) for syndromic management as per Ugandan clinical guidelines [31], together with the Multistix 8 test result for those with UTI symptoms. The trial clinical officer will provide training to the school nurses on symptom identification and referral.

#### Frequency and plans for auditing trial conduct {23}

The TSC meets approximately once per year to review trial conduct and progress. The TMG meets approximately every 6 weeks to review trial progress. The IDMEC meet twice a year to review safety issues and trial progress. The study sponsor (LSHTM) is responsible for organising monitors to review the source documents as needed, to determine whether the data reported are complete and accurate. The monitors will audit the overall quality and completeness of the data, examine source documents, interview investigators and coordinators, and confirm that the coordinating centre has complied with the requirements of the protocol. The monitors will verify that all adverse events were documented in the correct format and are consistent with protocol definition.

#### Plans for communicating important protocol amendments to relevant parties (e.g. trial participants, ethical committees) {25}

Protocol modifications which may impact on the conduct of the study and potential benefit of the patient or may affect patient safety will be approved by the ethics committees as a formal amendment prior to implementation. Administrative changes of the protocol are minor corrections and/or clarifications that have no effect on the way the study is to be conducted. These administrative changes will be agreed upon by the TMG and TSC and will be documented in a memorandum.

### Dissemination plans {31a}

Results will be disseminated among schools and local and national stakeholders to discuss policy implications of the findings. Policymakers and partners will be engaged through the Technical Working Groups (TWGs) for Ministry of Education and Ministry of Health. Communication with the wider academic community will include publication of the trial protocol and results as well as the economic, qualitative, and process evaluations in peer-reviewed academic journals with open access, guided by a dissemination plan.

## Discussion

Poor menstrual experience can adversely affect education, health, and well-being [5]. Our formative studies among Ugandan secondary school girls in Wakiso District found an unmet need for effective MH interventions to enable girls to better manage both the psychosocial and physical aspects of menstruation, supported by a broad consensus that MH interventions need to address menstrual stigma and literacy as well as the provision of products or improving WASH facilities [17]. We developed the MENISCUS multi-component intervention to address individual, social, behavioural, and environmental barriers to good MH, mental health problems, and educational engagement in line with these findings, and with the social cognitive theory underpinning our research.

To date, most previous MH interventions have focused on either puberty education, provision of menstrual products, or WASH improvements and have largely been conducted in rural primary schools [8, 26]. The MENISCUS intervention is innovative in evaluating a multi-component menstrual health intervention that includes all these components, and also pain management, inclusion of boys to address menstrual-related stigma and improve the school environment. It includes secondary schools in rural and peri-urban areas. The intervention has been co-designed with schools to be culturally appropriate, feasible to deliver, aligned with Government guidelines, cost-effective, environmentally-friendly, and practically sustainable within the schools. If the trial shows the intervention to be cost-effective and acceptable, it will have the potential to be scaled-up nationally and regionally. We will evaluate the implementation of current MoES guidelines in the control arm and in diverse settings, estimate the incremental cost and cost-effectiveness of delivering the MENISCUS package, and estimate scale-up scenarios following Ugandan government guidelines.

A recent systematic review highlighted the lack of evidence on the experience of women and girls participating in MH interventions [32]. The mixed-methods planned process evaluation will assess how the contextual factors (e.g. setting, school, staff, students) affect implementation and perceptions of the intervention. The qualitative data on mechanisms and context and the quantitative subgroup analyses will inform the potential transportability of the intervention. The policy analysis component will facilitate translation of findings to policy and inform refinement of the Ugandan Government MHM guidelines and implementation, including a better understanding of the lack of implementation of the current Circular.

## Trial status

Protocol version 3.0, January 2022, was approved by UVRI-REC on 11 February 2022. ISRCTN: 45461276

Recruitment began on March 21, 2022. Recruitment ended in June 2022.

## Supplementary Information

**Additional file 1.** SPIRIT checklist.

**Additional file 2.**

## Data Availability

Data will be available from the LSHTM Data Repository (https://datacompass.lshtm.ac.uk/) 12 months after trial completion. Trial materials are available at the trial website (https://www.lshtm.ac.uk/research/centres-projects-groups/meniscus).

## Abbreviations

CHU9D: Child Health Utility 9-dimension questionnaire
CRF: Case report form
ICF: Informed consent form
IDI: In-depth interview
IDMEC: Independent Data Monitoring and Ethics Committee
MH: Menstrual health
MHM: Menstrual hygiene management
MoES: Ministry of Education and Sports
MOH: Ministry of Health
MRC: UK Medical Research Council
NGO: Non-governmental organisation
TMG: Trial Management Group
TSC: Trial Steering Committee
UNCST: Uganda National Council for Science and Technology
UNEB: Uganda National Examination Board
UTI: Urinary tract infection
UVRI: Uganda Virus Research Institute
WASH: Water, sanitation, and hygiene
