# Supplementary material for: Menstrual health interventions, schooling, and mental health problems among Ugandan students (MENISCUS): study protocol for a school-based cluster-randomised trial
Source: Trials. 2022 Sep 7;23:759. doi: 10.1186/s13063-022-06672-4 (PMC9449307; doi:10.1186/s13063-022-06672-4)
Supplement: Supplementary file 2 — Additional file 2. [file 13063_2022_6672_MOESM2_ESM.zip › AN0505~1R1.PDF]

MRC/UVRI and LSHTM Uganda Research Unit

MRC Medical Research Council | Uganda Virus Research Institute | LONDON SCHOOL of HYGIENE &TROPICAL MEDICINE

Ekiwandiiko ekiriko amawulire agasaba abazadde/abalabirira abayizi abanaawebwa ettu ly'ebikozesebwa mu nsonga z'ekikyala mu kunonyereza kwa MENISCUS.

**Project title:**	Menstrual health interventions, schooling and mental health symptoms among Ugandan students (MENISCUS): a school-based cluster-randomised trial
**Funder:**	UK Joint Global Health Trials (Medical Research Council-Department for International Development-Wellcome Trust) Grant # MR/V005634/1
**Research Site:**	Wakiso and Kalungu Districts<br>C/o MRC/UVRI and LSHTM Uganda Research Unit<br>Plot 51-59, Nakiwogo Road<br>P O Box 49, Entebbe, Uganda<br>Tel: +256(0) 417 704000; (0)312 262910/1; (0)702 438487
**Principal Investigators:**<br><br><br><br>**Local Principal Investigator:**	**1. Prof Helen Weiss,**<br>Professor of Epidemiology and Director of the MRC Tropical Epidemiology Group, London School of Hygiene and Tropical Medicine (LSHTM), UK<br>*Email: helen.weiss@lshtm.ac.uk*<br>**2. Prof Janet Seeley**<br>Professor of Anthropology and Health, London School of Hygiene and Tropical Medicine (LSHTM), UK<br>and Head of Social Science Programme, MRC/UVRI and LSHTM Uganda Research Uni<br>*Email: janet.seeley@lshtm.ac.uk*
**Trial Manager:**	Dr. Catherine Kansiime,<br>MRC/UVRI and LSHTM Uganda Research Unit<br>*Email:Catherine.Kansiime@mrcuganda.org*

Mu bufunze (By'olina okumanya ku kunoonyereza kuno):

Ekigendererwa ky'okugezesa kuno kwe kulaba oba nga ettu lya MENISCUS lilongosa ebyenjigiriza/ebyekusoma, obubonero obulabirwako eby'obulamu ebikwata kubwongo, okukwata obulunji ensonga z'ekikyala awamu n'embeela y'obulamu bwabwe mu mamasomero ga siniya mu district ze Wakiso ne Kalungu mu Uganda.

- Ekiwandiiko kino kinnyonnyola ekigendererwa ky'okunoonyereza kuno n'omwana wo ky'anasabibwa okukola singa onooba omukkirizza okwetabamu.
- Okw'etaba kw'omwanawo mu kunoonyereza kuno kwa kyeyagalire. Dembe lyo gwe n'omwanawo okusalawo obutetabamu, oba okukkiriza okukwetabamu kati era oluvannyuma n'emukyusa endowooza yammwe/nemukuvaamu.
- Kyonna ky'anaaba asazeewo tekijja kukosa ngeri gwe oba omwana/muwala wo jafunamu bujjanjabi wadde obuyambi n'akatono.
- Yita/soma ekiwandiiko kino n'obwegendereza. Buuze ebibuuzo byonna by'oyagala nga tonasalawo.

UVRI-RESEARCH ETHICS COMMITTEE
VALID UNTIL
26 MAY 2023
APPROVED
P. O. BOX 49, ENTEBBE (U)

Ojja kuweebwa kopi ku kiwaandiiko kino

Ekitundu Ekisooka: Ebikwata ku kunoonyereza kuno

Enyanjula:

Okunonyereza kwa MENISCUS - trial, kukulembedwa MRC/UVRI ne LSHTM Uganda Research Unit okuva Entebbe n'ettendekero lya London School of Hygiene and Tropical Medicine mu United Kingdom nga bakolaganira wamu ne WoMena Uganda.

Tukola Okunoonyereza kuno okulungamya amasomero ga Siniya okuzuula engeri ezisoboka ez'okuyamababamu abaana abawala okubeera n'okusigala abalamu era bamalirize okusoma okuyita mu nkwata y'ensonga z'ekikyala erongoseddwamu. Twafunye olukusa okukola okunonyereza kuno okuva kubakulu b'essomero lyo, district, ekitongole ky'ebyenjigiriza n'emizannyo n'obukiiko obulondoola okunonyereza obwa UVRI, LSHTM awamu ne Uganda National Council of Science and Technology Omwana/muwalawo asabiddwa okwetaba mukunonyereza kuno, osobola okusalawo oba omwana/muwala wo yetaba mukunonyereza kuno oba nedda. Oli wa ddembe okutubuuza ebibuuzo kati or oluvannyuma ng'okozesa emikutu gyaffe egyempuliziganya ezitereddwa wamanga. Tujja kutwala obudde okukunnyonnyola.

Ekigendererwa:

Ekigendererwa ky'okunoonyereza kwa MENISCUS trial kwe kulaba oba nga enkola yokutumbula eby'obulamu mu mumasomero ga siniya enayambako mu kulongosa ensonga z'ekikyala (engeri abaana abawala jebasobola okubeera obulunji n'obuvumu nga bali mu nsonga z'ekikyala). Twagala kumanya oba nga ettu liyinza okuyambako mu kulongosa ebyenjigiriza/eby'okusoma, eby'obulamu mubana abawala awamu n'okumanya n'endowooza za baana abalenzi kubikwata kusonga za bakyala. Okunonyereza kunno bwekunaba kuvudemu ebirungi, kujja kutongozebwa mumasomera amalala mu Uganda.

Okulonda

Omwana wo alondeddwa okwetaba mukunonyereza kuno olw'ensonga nti muyizi mulimu ku masomera amakumi asatu (30) agagwa ku mukono gw'ago aganafuna ettu lya MENISCUS era oba nga ekisooka yetaba mu kibiina kya 'Menstrual Health Action Group oba ekyokubiri nga muyizi abanguddwa/atendekeddwa okuyamba ku banne kunsonga ezekuusa kunsonga zekikyala.

Okwetabamu kwakyeyagalire

Okwetaba mu kunoonyereza kuno kwa kyeyagalire. Ggwe oba omwana wo muli baddembe okugaana. Okusalawo obuteegatta mu kunoonyereza kuno tekijja kukosa gwe ne famileyo bye mulina kufuna ku somero wadde ewajjanjabirwa wonna. Oli wa ddembe okutubuuza ebibuuzo byonna byoyagala era tuli beetegefu okubyanukula. Osobola obutasalawo kati/leero, oli waddembe okusooka okukirowoozaako n'otubuulira oluvannyuma ky'onooba osazeewo. Ggwe oba omwana wo musobola okulekerawo okwetabamu/okuva mukunonyereza kuno essawa yonna.

Emitendera:

Okunonyereza kuno kukoleddwa wakati w'omwaka 2021 n'omwaka 2023 mu masomero 60 agalondeddwa mu Wakiso ne Kalungu Districts. Ku gano, amasomero 30 gajja kutekebwa kukalulu galondebwe okufuna ettu lya MENISCUS omuli okusomesebwa ku nsongo z'okuvubuka n'ensonga z'ekikyala, okulongoosa kabuyonjo z'essomero, omukisa okwetaba mu mizannyo gyakatemba egyekuusa ku nsonga z'ekikyala n'okuwebwa ettu ly'ebikozesebwa mu nsonga z'ekikyala wamu n'enkola ezikendeeza obulumi.

<u>Okuwebwa ettu ly'ebikozesebwa mu nsonga z'ekikyala</u>

UVRI-RESEARCH ETHICS COMMITTEE
VALID UNTIL
26 MAY 2023
APPROVED
P. O. BOX 49, ENTEBBE (U)

Omwana wo ajja kuwebwa ettu omuli paadi ezozebwa neziddamu nezikozesebwa (AFRIpads) nga zitereddwa mu nsawo, obupale bw'omunda, eccuppa y'amazzi, sabbuuni, akatawulo wamu n'omukebe ogw'ekyuma.

Bajja kwetaba mu misomo enakulemberamu omutendesi ku ngeri y'okukozesaamu paadi, ez'ozebwa neziddamu nezikozesebw. Omutendesi ajja kulaga omwana wo engeri paadi ezozebwa ne ziddamu nezikozesebwa gyezikozesebwamu era bajja kukubagany ebirowoozo ku bweralikirivu bwonna bwebayinza okuba nabwo kunsonga eno. Abetabyemu abawala bajja kusabibwa okugezaako okukozesa ebikozesebwa mu nsonga zekikyala okumala ebbanga ly'omwaka gumu ogunaddako, bwebaba nga bagala/bawulira emirembe okukikola. Bwebanaaba nga balina obuzibu bwebasanze mu ttu ly'ebikozesebwa munsonga, bajja kusobola okukyogerako n'omukulembeze, omutendesi omukugu oba Clinic Officer w'okunonyereza kuno.

Okutendekebwa okusosbols okusomesa abalala ku nsonga z'ekikyala

Bw'anaaba alondeddwa, omwanawo ayinza okusabibwa okwetaba mukutendekebwa ku ngeri y'okusomesaamu abalala kubyekuusa kukwatamu ensonga z'ekikyala. Okutendeka kuno kujja kukulemberwamu abatendesi abamanyirivu okuva mu WoMena Uganda era abajja kwanukula ebibuuzo byonna oba ensonga zonna zebayinza okuba nazo nga tebanasomesa balala

Okubuzibwa ebibuuzo okwa sekinoomu (eddakiika 60):

Omwana wo ayinza okusabibwa okunyumyamu/okwetaba mukubuzibwa ebibuuzo bya sekinoomu n'omu kubanonyereza atendekeddwa, okumubuuza kw'ebyo byayiseemu nga yetaba mu Action Group, mukutendekebwa kubikwata ku nsonga z'ekikyala, ne/oba okukozeza ebikozesebwa mu nsonga z'ekikyala. Okukubaganya ebirowoozo kuno kujja kuba mukifo ekikanyizidwako munda musomero. Okukubaganya ebiriwoozo kwonna kuyinza okwatibwa ku katambi akakwata amaloboozi. Obutambi obwo bwa kusibirwa mu kabada ku MRC/UVRI and LSHTM Uganda Research Unit. Ebinaakwatibwa ku butambi bya kukumibwa nga bya kyama era tewali ajja ku biwulirako okujjako abakola ku kunoonyereza abakkirizibwa mu mateeka agafuga okunoonyereza okugeza abawandiika ebikwattiddwa kubutambi, abatekamu ensimbi oba obukiiko obulondoola n'okulabirira okunonyereza era nga bagereddwa ku butambi bajja kukirizibwa okuwuliriza obutambi.

Obutyabaga n'okuteteganyizibwa: Kino kibi oba kya bulabe eri gy'oli?

Tuyinza okubuuza omwana wo ebimukwatako ng'omuntu, okugeza by'ayitamu olw'okwetaba mu Action Group ez'ekuusa ku nsonga z'ekikyala ku ssomero ne/oba endowooza z'abwe ku nsonga z'ekikyala. Ayinza obutawulira bulungi nga oby'ogerako.

Omwana wo ajja kusomesebwa ku ngeri y'okwoza n'okukaza paadi neziddamu nezikozesebwa, okuzuula obubonero bw'obulwadde n'ani gw'alina okulaba/okutukirira.
Singa oba togoberedde biragiro bino wayiza okubalukawo akatyabaga kokukwatibwa obulwadde oba obutawulira bulungi mukukozesa paadi zino. Wabula wewaberawo akatyabaga konna akaamanyi oba ebizibu mukukozesa paadi ezozebwa, ggwe oba omwana wo ajakubera waddembe okutukirila omusawo w'esomero asobole okuyambibwa oba okuwerezebwa awala asobole okuyambibwa. Tulina omusawo waffe (Clinic Officer) yo awerezebwa obutyabaga/obuzibu obwamanyi. Omwana wo asabibwa okwogera amangu singa aba afunyemu okusomozebwa kwonna mukukozesa ettu ly'ebikozesebwa mu nsonga z'ekikyala.

Okuganyurwa (benefits): Waliwo ekirungi kyonna ekinakutukako mukwetabamu?

Okwetabamu kw'omwana wo kujja kutuyamba, amasomero, ebifo ebijjanjabirwamu n'ebyenjigiriza awamu n'abavunanyizibwa ku by'obulamu okwongera okuzuula kubyobulamu byammwe n'empereza ezetagibwa.

UVRI-RESEARCH ETHICS COMMITTEE
26 MAY 2023
APPROVED
P. O. BOX 49, ENTEBBE (U)

Okusasulwa: Onaafuna ekintu knyonna olw'okwetaba mu kunoonyereza kuno?
Omwana wo tajja kusasulwa olw'okwetaba mukunonyereza kuno. Wabula, bwanaba olondeddwa okwetaba mukubuzibwa ebibuuzo ebya ssekinoomu, ajja kuwebwa bbayiro, akatabo k'eddiba eggumu n'akokunywa akogonvu kulw'obudde ne kawefube gw'anaaba bataddemu. Ojja kuwebwayo omutwalo gumu 10000 olwobudde bwo nekawefube gw'onaba otaddemu.

Emmizi (Confidentiality): Waliwo omuntu yenna agenda okumanya ku kino?
Tetujja kubuulira bantu balala nti omwana wo yeetabye mu kunoonyereza kuno. Tetujja kugabana mawulire agategeeza nti yemwana wo n'omuntu yenna atakola kukunonyereza kuno. Ekikwata ku mwana wo kyonna, tujja kuba tukozesa namba (study number) mu kifo ky'e linnyalye. Wabula ebibakwatako biyinza okulabibwako ba auditors.

Okutegeezebwa ebinaazuulibwa: Onootegeezebwa ebinaazuulibwa mu kunoonyereza kuno?
Okunoonyereza kuno nga kuwedde tujja kutegeeza muwala wo ebinaaba bizuuliddwa. Era tujja kubitegeeza abazadde/abalabirira abaana, abakulira essomero lino ko aba Munisipaali ne Gwanga lyonna okutwalira awamu omuli nebyo byetunaba tuyize.

Oluvanyuma tujja kubitegeeza n'abantu abalala omuli ba Nasayansi, abakola ku by'obulamu, n'abantu abalala kubinaaba bizuliddwa. Kino tujja kikola nga tuyita mu kuwandiika zi alipoota, n'okusisinkana bonna be kikwatako. Ebinaava mu kunoonyereza kuno era bya kuteekebwa mu butabo bwa sayansi obw'ensi yonna (international science journals) ko n'emikutu ja intaneti abantu abalala basobole okutuyigirako. Wabula ebinava mukunonyereza kuno tebijja kuwandikibwa mungeri ekkiriza omuntu yenna okujjako abakola kukunonereza kuno okumanya omwana byeyatugamba oba ebyo ebimukwatako ng'omuntu ebeimigiddwako. Ebivudde mukunonyereza kuno era biyinza okutekebwa kumukutu okuyita ku London School of Hygiene and Tropical medicine (data repository. Kino kitegeza nti tuyinza okubikozesa okwongera okubyekenenya. Amawulire gonna gasunsulwa mungeri en'ekusifu ekitegeeza; tegasobola kwekuusa ku mwana wo oba kutegeeza nti oyo ye mwana wo.

Ani gw'oyinza okutukirira: Ani gw'oyinza okwogerako naye oba okubuuza ebibuuzo ebyekuusa ku kunonyereza kuno.

Osobola okutubuuza ebibuuzo kati oba oluvannyuma ng'oyita ku ssimu, e-mail, post oba kundagiriro yaffe eragiddwa kulupapula olusaba okwetabamu olunakuwebwa. Bw'oba oli kumpi, osobola okujja n'otulaba.
Oyinza okutukirira abantu bano wammanga kunsonga ezikwata kukunonyereza kuno
a) Dr.Catherine Kansiime, MENISCUS trial Project Lead
Email: catherine.kansiime@mrcuganda.org Phone number +256 702438487
Bwoba olina ekibuuzo oba okwemulugunya ku ddembelyo ku by'okwetabakwo mu kunoonyereza kuno tuukirira akakiiko ka UVRI akalondoola n'okulabirira okunonyereza ku simu
+256 0414 321962 oba +256 716 321962.

EKITUNDU II: Okukkiriza okwetabamu (VERSION 1.0 JANUARY 2022)
Nga ntekako omukono wammanga , nzikiriza omwana wange okwetaba mu kunonyereza kuno nga bwekulambikiddwa/bwekunnyonnyoddwa waggulu nga mulimu;

- Okufuna ettu ly'ebikozesebwa munsonga z'ekikyala n'okutendekebwa ku ngeri y'okubikozesa mu.
- Okwetaba mukubuzibwa ebibuuzo bya ssekinoomu singa aba aloneddwa.
- Amawulire gonna agakunganyiziddwa mu ngeri enekusifu okukozesebwa mukunonyereza kuno n'okugabanibwako n'abanonyereza abalala.

Ebibuuzo byange ebyekuusa kukunonyereza kuno bididdwamu……………………………………………

UVRI-RESEARCH ETHICS COMMITTEE
VALID UNTIL
26 MAY 2023
APPROVED
P. O. BOX 49, ENTEBBE (U)

Soma ebibuzo bino wamanga	Londako Ye oba nedda	
Osomye/ bakusosomedde ebikwata/amawulire agakwata ku kunonyereza kuno?	Yee	Nedda
Waliwo omuntu omulala yenna akunnyonyodde kukunonyereza kuno?	Yee	Nedda
Otegedde kiki okunonyereza kuno kyekukwatako?	Yee	Nedda
Ebibuuzo byo bikuddiddwamu mungeri gy'otegeera?	Yee	Nedda
Otegedde nti oli waddembe okuva mu kunoonyereza kuno wonna woba oyagalidde?	Yee	Nedda
Oli musanyusa okwetaba mukunonyereza kuno?	Yee	Nedda

Erinnya ly'etabyemu: ______________________________ School ID: |__|__|__|

Erinnya ly'omuzadde/alabirira omwana: ______________________

Omukono gw'omuzadde/alabirira omwana: ____________________________________

Ennaku z'omwezi (olunaku/omwezi/omwaka): |__|__|/|__|__|/|__|__|__|__|

Bw'aba tasobola kusoma na kuwandiika: Omujulizi asobola okusoma n'okuwandiika alina okutekako omukono. (bwekiba kisoboka, omuntu ono alina okulondebwa eyetabyemu era talina kuba nankolagana yonna n'abakola kukunonyereza kuno). Omuzadde/alabirira omwana atasobola kusoma nakuwandiika atekeko ekyenkumu kye.

Erinnya ly'omujulizi ____________________ **ne** **Ekyenkumu ky'eyetabyemu**

Omukono gw'omujulizi____________________

Ennaku z'omwezi ____________________ **Olunaku/omwezi/omwaka**

Omunonyereza: Nkakasa nti omuntu ono asabidwa olukusa era akiriza okwetaba mu kunonyereza kuno nga yeyagalidde.

Erinya ly'anonyereza: ____________________ Enaku z'omwezi:|__|__|/|__|__|/|__|__|__|__|

dd / mm / yyyy

Omukono: ________________

UVRI-RESEARCH ETHICS COMMITTEE
VALID UNTIL
26 MAY 2023
APPROVED
P. O. BOX 49, ENTEBBE (U)

UVRI-RESEARCH ETHICS COMMITTEE
VALID UNTIL
26 MAY 2023
APPROVED
P. O. BOX 49, ENTEBBE (U)
