# Supplementary material for: Menstrual health interventions, schooling, and mental health problems among Ugandan students (MENISCUS): study protocol for a school-based cluster-randomised trial
Source: Trials. 2022 Sep 7;23:759. doi: 10.1186/s13063-022-06672-4 (PMC9449307; doi:10.1186/s13063-022-06672-4)
Supplement: Supplementary file 2 — Additional file 2. [file 13063_2022_6672_MOESM2_ESM.zip › AN2263~1R1.PDF]

MRC/UVRI and LSHTM Uganda Research Unit

MRC Medical Research Council | Uganda Virus Research Institute | LONDON SCHOOL of HYGIENE &TROPICAL MEDICINE

Ekiwandiiko ekiriko amawulire agasaba olukusa abakyala abakola mu masoomero oba abalabirira abaana okukiriza abaana abawala okufuna akakopo akakozesebwa mu nsonga z'ekikyala mu kunonyereza kwa MENISCUS.

**Project title:**	Menstrual health interventions, schooling and mental health symptoms among Ugandan students (MENISCUS): a school-based cluster-randomised trial
**Funder:**	UK Joint Global Health Trials (Medical Research Council-Department for International Development-Wellcome Trust) Grant # MR/V005634/1
**Research Site:**	Wakiso and Kalungu Districts<br>C/o MRC/UVRI and LSHTM Uganda Research Unit<br>Plot 51-59, Nakiwogo Road<br>P O Box 49, Entebbe, Uganda<br>Tel: +256(0) 417 704000; (0)312 262910/1; (0)702 438487
**Principal Investigators:**<br><br><br><br>**Local Principal Investigator:**	**1. Prof Helen Weiss,**<br>Professor of Epidemiology and Director of the MRC Tropical Epidemiology Group, London School of Hygiene and Tropical Medicine (LSHTM), UK<br>*Email: helen.weiss@lshtm.ac.uk*<br>**2. Prof Janet Seeley**<br>Professor of Anthropology and Health, London School of Hygiene and Tropical Medicine (LSHTM), UK<br>and Head of Social Science Programme, MRC/UVRI and LSHTM Uganda Research Uni<br>*Email: janet.seeley@lshtm.ac.uk*
**Trial Manager:**	Dr. Catherine Kansiime,<br>MRC/UVRI and LSHTM Uganda Research Unit<br>*Email:Catherine.Kansiime@mrcuganda.org*

Mu bufunze (By'olina okumanya ku kunoonyereza kuno)

- Ekigendererwa ky'okunoonyereza kuno kwekuzula obanga okugaba ebikozesibwa munsonga z'ekikyala wamu ne misomo ku nsonga z'ekikyala kinayamba kukusoma, obubonero ku bulamu bw'obwongo, okwerabirira nga ali munsonga z'ekikyala awamu n'embela yobulamu mu baana abawala mu mamasomero ga secondary e Wakiso ne Kalungu mu Uganda.
-

    - Ekiwandiiko kino kinnyonnyola ekigendererwa ky'okunoonyereza kuno ne ky'onasabibwa okukola singa okiriza okukwetaba mu kunoonyereza kuno.

      Okw'etaba kw'o mu kunoonyereza kuno kwa kyeyagalire. Dembe lyo okukwetabamu, oba okukwetabamu oluvannyuma n'okyusa endowooza yo.

UVRI RESEARCH ETHICS COMMITTEE
VALID UNTIL
2 6 MAY 2023
APPROVED
P. O. BOX 49, ENTEBBE (U)

Kyonna ky'anaaba asazeewo tekijja kukosa ngeri jafunamu bujjanjabi wadde obuyambi.

Soma ekiwandiiko kino n'obwegendereza era obuuze ekibuuzo kyonna ky'oyagala nga tonasalawo.

Ojja kuweebwa kopi ku kiwaandiiko kino okitereke

Ekituundu ekisookal: Ebikwata ku kunoonyereza kuno

Enyanjula:
Okunoonyereza kwa MENISCUS ku kulembeddwamu ekitongole kya MRC/UVRI ne Tendekero lya London School of Hygiene and Tropical Medicine (LSHTM) nga bakolerera wamu n'ekitongole kya WOMENA Uganda.

Tukola Okunoonyereza kuno okulungamya amasomero ga Siniya okuzuula engeri ezisoboka okuyambamu abaana abawala okubeera abalamu era bamaririze emisomo gyabwe ku somero nga tuyita mukulungosa engeri abaana abawala gyeberabirila nga bali munsonga z'ekikyala.

Twafunye olukusa okukola Okunoonyereza kuno okuva eri abatwala e Somero lino, okuva ku disitirikiti, okuva mu kitongole ky'ebyenjigiliza n'emizanyo n'okuva mu bukiiko obulondoola okunoonyereza obwa MRC/UVRI ne LSHTM.

Tukusaba okirize weteba mu kunoonyereza kuno. Ddembe lyo okusaalawo okwetaba mu kunoonyereza kuno oba nedda. Oli waddembe okutubuuza ekibuuzo kyonna ky'oyagala kati oba oluvannyuma ng'oyita ku email ne namba z'esimu eziragiddwa wa manga era tujja kutwala obuvunaanyizibwa tukunnyonnyole otegeere.

Ekigendererwa
Ekigendererwa ky'okunoonyereza kwa MENISCUS kwe kulaba oba nga enkola yokutumbula eby'obulamu mu mumasomero ga siniya enayambako mu kulongosa ensonga z'ekikyala (engeri abaana abawala jebasobola okubeera obulungi nga bali mu nsonga z'ekikyala)

Twagala okuzuula oba ettu lya MENISCUS lijja kusobola okulongosa eby'okusooma, eby'obulamu n'okubeera obulungi eri abaana abawaala era n'okumanya okutegera ne neyisa y'abaana abalenzi ku nsonga z'ekikyala.

Okunonyereza kunno bwekunaba kuvudemu ebirungi, kujja kutongozebwa mumasomera amalala mu Uganda. Nga ekimu mukunoonyereza kuno boona abaana abawala mu siniya ey'okubiri bajja webwa padi ezikozesibwa nezidamu nezikozesibwa.

Okulonda:
Tusaaba abaamu kubakola mu masomero n'abazadde b'abaana mu masomeero ga siniya okwetaba mu kunoonyereza kwa MENISCUS n'okufuna akakopo akakozesibwa munsonga z'ekikyala nga bo' Olunodedwa kubanga i) omwanawo muyizi kusomeero erimu erigenda okufuna ettu lya MENISCUS ii) wetaba mu kibina ekitumbula eb'obulamu ku someero.

Okwetamu kwakyeyagalire:
Okwetaba mu kunoonyereza kuno kwa kyeyagalire. oyinza okusaalawo okugana okukwetabamu. Okusalawo obuteegatta mu kunoonyereza kuno tekijja kukosa gwe ne famile yo bye mulina okufuna ku somero wadde ewajjanjabirwa wonna. Oli wa ddembe okutubuuza ebibuuzo byonna era tuli beetegefu okubyanukula. Osobola obutasalawo kati Oli waddembe okusooka okukirowoozaako n'otubuulira oluvannyuma ky'onooba osazeewo. Oliwadembe okuva mu kunoonyereza kuno ekiseera kyona.

UVRI RESEARCH ETHICS COMMITTEE
VALID UNTIL
26 MAY 2023
APPROVED
P. O. BOX 49, ENTEBBE (U)

Emitendera:
Okunoonyereza kuno kukolibwa wakati w'omwaka 2021 ne 2023 mu masomeero ga siniya 60 agalondidwa. Kwago 60 amakumi assatu (30) kugo aganaba galondedwa bajja kufuna ettu lya MENISCUS omuli Emisomo ekikwatagana kukikula kyabwe ne nsonga z'ekikyala, okudabiriza kabuyonjo ye someero, omukisa okwetaba mu katemba akwatagana ku nsonga z'ekikyala wamu nokugaba ebikozisibwa mu nsonga z'ekikala omuli nakakopo akakozisibwa mu nsonga z'ekikyala.

Nga ekimu kubikozesibwa mu nsonga z'ekikyala, ojja kuba n'okusaalawo okukiriza oba obutakiriza kuffuna kakopo kakozesibwa mu nsonga z'ekikyala (Ruby Cup). Akakopo kano kagonvu nga kazingibwa ne kasonsenkebwa mu bukyala ne kagendamu omusaayi gw'ensonga z'ekikyala era omuntu asobola okukambala obudde bw'emisana bwonna n'ekiro nga tekajjudde kuyiika. Bwe kajjula okajjayo mu bukyala n'oyiwa ebirimu, n'okooza balunji n'okazzaayo. Abawala/Abakyala kibetagisa okukajjayo ne bayiwa omusaai ku makya n'olweggulo Bwanakuguka mukakozesa, akakozesa ajja kumanya ddi lwalina okukajayo nayiwa omusaayi ogulimu okusinzira kubyetago bye. Bw'osalawo obutakiriza kuwebwa kakopo kakozesibwa munsonga z'ekikyala ojja kusigala nga offuna ebikozisibwa mu nsonga z'ekikyala ebirala.

Ojja kusabibwa okwetaba mu musomo ogunaakubirizibwa omusomesa atandekidwa mu nsonga z'ekikyala ku ngeri y'okozesa akakopo akakozesebwa mu nsonga z'ekikyala. Bajja kulaga engeri y'okukozesaamu Akakopo era era bajja kunnyonnyola buli ky'onaaba oyagala okumanya ku nsonga eno. Ojja kusabibwa okukozesa akakopo okumala omwaka ogunaddako bw'oba nga tokirinamu buzibu. Bwofunamu obuzibu bw'ona mu koozesa ebikozesibwa mu nsonga z'ekikyala ojja kuba ojja kuba n'omukisa okunyumya n'omu kubakula bekibinja, omukugu mu nsonga z'ekikyala oba omusaawo ku kunoonyereza kuno.

Obutyabaga n'okuteteganyizibwa: Kino kibi oba kya bulabe eri gy'oli?

Obukopo buno bukozesidwa mu nsi eziwerako nga ne Uganda mweri era tebulina nyo bulabe bwebuleta eri obulamu bwaffe. Ojja kusomesibwa okozesa akakopo. Akakopo bwe kamala okusonsekebwayo abakyala abasinga obungi tebalina bulumi bwe bafuna. Naye era oyinza okuwuliramu obubi nga okateekayo oba nga okajjayo (naddala emirundi egisooka), era oyinza okuwulira ensonyi oba okutya nga okasonseka mu bukyala.

Waliwo akatyabaga k'okufuna allergy nga eva ku matiriyo ekozebwa mu kukola akakopo kano (silicone), naye kino tekitera kubaawo. Singa oba nga ofunye obulumi oba okusiyiibwa oba okuzimba mubitundu byo eby'ekyama oba obulumi bwona nga afuyisa, olina okujayo akakopo n'okwogera amangu eri omusawo oba akulira okunonyereza kunno.
Obukopo buno bukozesebwa abakyala banji nnyo mu nsi eziwerako era waliwo esonga emu yooka ewandikibwako eyomukyala eyafuna embeera eyitibwa (toxic shock syndrome) bweyakozesa akakopo akayitibwa DivaCup. Embera eno eleta omusujja, senyiga, kamunguluze ela osobola okulwala. Toxic shock syndrome tetela kubelawo ate ela tewulilwangako nga eva kukozesa (Ruby cup). Akokopo ketugenda okugaba, singa oba ofunye obubonero bunno nga olimunsonga z'ekikyala oyina okujjayo akakopo amangu ddala oba okugenda mumaso n'oyogera amangu eri omusawo oba akulira okunoonyereza kunno kunambaye ey'esimu. Bw'oba nga waffunako ku toxic shock syndrome emabega okubirizibwa obutakoseza kintu kyona nga kiyingila mu bukyalabwe okugeza akakopo ne tampon. Ojja kusomesebwa engeri y'okwozaamu akakopo. Wayinza okubalukawo akatyabaga kokukwatibwa obulwadde singa akakopo kaba tekalongosedwa bulungi.

UVRI-RESEARCH ETHICS COMMITTEE
VALID UNTIL
26 MAY 2023
APPROVED
P. O. BOX 49, ENTEBBE (U)

Okuganyurwa: Waliwo ekirungi kyonna ekinakutukako mukwetabamu?

Owereddwa eddembe okulondako kubikozesebwa munsonga z'ekikyala, oyinza okusalawo/okwagala okukozesa obukopo okusinga ku paadi ez'ozebwa neziddamu neziozesebwa. Okwetabamukwo kujja kutuyamba, kuyamba amasomero, malwaliro/ebitongole by'obulamu wamu n'abavunanyizibwa kuby'obulamu okwongera okuzuula kubyobulamu byammwe n'empereza ezetagibwa. Tusubira nti bino bijja kuyamba abo bonna bekikwatako okutuukiriza burungi ebyetago ebyo gyebujja.

Okusasulwa: Onaafuna ekintu knyonna olw'okwetaba mu kunoonyereza kuno?
Ojja kuddizibwawo omutwalo gumu 10000 olwobudde bwo nekawefube gw'onaba otaddemu. Ojja kufuna okutendekebwa ku nsonga z'ekikyala, ebyokunywa mukutendekebwa n'okuddizibwa ebisale by'entambula (transport) singa omusomo/okutendekebwa kunaba kutegekeddwa wabweru we ssomero.

Okukuma ebyama: Ebintu bino binaamanyibwako abantu abalala?
Tewali gwe tujja kubuulirako nti wetabye mu kunoonyereza kuno. Tewali muntu yenna atakola mu kunoonyereza kuno gwe tujja kubuulirako ku bimukwatako era tujja kuba tukozesa namba mu kifo ky'e linnyalye. Wabula amawulire g'otuwadde gayinza okulabibwako ba auditors.

Okutegeezebwa ebinaazuulibwa mu kunoonyereza: Onootegeezebwa ebinaazuulibwa mu kunoonyereza kuno?

Okunoonyereza kuno nga kuwedde tujja ne kutegeezebwa ebinaaba bizuuliddwa era tujja kubitegeeza abazadde, abalabirira abaana, abobuyinza ku masomero ko n'aba Munisipaali ne Gwanga lyonna okutwalira awamu omuli nebyo byetunaba tuyize. Oluvanyuma tujja kubitegeeza n'abantu abalala omuli ba Nasayansi, abakola ku by'obulamu, n'abantu abalala. Kino tujja kikola nga tuyita mu kuwandiika zi alipoota, n'okusisinkana bonna be kikwatako. Ebinaava mu kunoonyereza kuno era bya kuteekebwa mu butabo (journals) bwa sayansi obw'ensi yonna ko n'emikutu ja intaneti abantu abalala basobole okutuyigirako. Ebivudde mukunonyereza kuno. era biyinza okutekebwa ku mukutu gwa London School of Hygiene and Tropical medicine abantu abalala gyebayinza okubisanga. Kino kitegeza nti tuyinza okudamu okwekenenya ebinaba bivudde mukunonyereza naye nga tewali ngeri yonna mu kwogera ebinaava mu kunoonyereza kuno muwalawo bye yatubuulira ng'omuntu we bijja kulabikira.

Ani gw'oyinza okutukirira: Ani gw'oyinza okwogerako naye oba okubuuza ebibuuzo ebyekuusa ku kunonyereza kuno.
Osobola okutubuuza ebibuuzo kati oba oluvannyuma ng'oyita ku ssimu, e-mail, post oba kundagiriro yaffe eragiddwa kulupapula olusaba okwetabamu olunakuwebwa. Bw'oba oli kumpi, osobola okujja n'otulaba.

Oyinza okutukirira abantu bano wammanga kunsonga ezikwata kukunonyereza kuno

a) Dr.Catherine Kansiime, MENISCUS trial Project Lead
Email: catherine.kansiime@mrcuganda.org Phone number +256 702438487

Bwoba olina ekibuuzo oba okwemulugunya ku ddembelyo ku by'okwetabakwo mu kunoonyereza kuno tuukirira akakiiko ka UVRI akalondoola n'okulabirira okunonyereza ku simu
+256 0414 321962 oba +256 716 321962.

UVRI-RESEARCH ETHICS COMMITTEE
VALID UNTIL
26 MAY 2023
APPROVED
P. O. BOX 49, ENTEBBE (U)

EKITUNDU II: Okukkiriza okwetabamu (VERSION 2.0 JANUARY 2022)

Nga ntekako omukono wammanga, nzikiriza okwetaba mu kunonyereza kuno nga bwekulambikiddwa/bwekunnyonnyoddwa waggulu nga mulimu;

- Okufuna akakopo akakozesebwa mu nsonga z'ekikyala n'okutendekebwa ku ngeri y'okukakozsaamu nga akamu ku ttu lyebikozesebwa munsonga zekikyala.
- Amawulire gonna agakunganyiziddwa mu ngeri enekusifu okukozesebwa mukunonyereza kuno n'okugabanibwako n'abanonyereza abalala.

Ebibuuzo byange ebyekuusa kukunonyereza kuno bididdwamu...

Soma ebibuzo bino wamanga	Londako Ye oba nedda	
Osomye/ bakusosomedde ebikwata/amawulire agakwata ku kunonyereza kuno?	Ye	Nedda
Waliwo omuntu omulala yenna akunnyonyodde kukunonyereza kuno?	Ye	Nedda
Otegedde kiki okunonyereza kuno kyekukwatako?	Ye	Nedda
Ebibuuzo byo bikuddiddwamu mungeri gy'otegeera?	Ye	Nedda
Otegedde nti oli waddembe okuva mu kunoonyereza kuno wonna woba oyagalidde?	Ye	Nedda
Oli musanyusa okwetaba mukunonyereza kuno?	Ye	Nedda

Erinnya ly'etabyemu : ______________________ School ID: |__|__|__|:

Omukono gw'eyetabyemu: ______________________

Ennaku z'omwezi (olunaku/omwezi/omwaka): |__|__|/|__|__|/|__|__|__|__|

Bw'aba tasobola kusoma na kuwandiika: Omujulizi asobola okusoma n'okuwandiika alina okutekako omukono. (bwekiba kisoboka, omuntu ono alina okulondebwa eyetabyemu era talina kuba nankolagana yonna n'abakola kukunonyereza kuno). Omuzadde/alabirira omwana atasobola kusoma nakuwandiika atekeko ekyenkumu kye.

Erinnya ly'omujulizi ______________________ NE Ekyenkumu
ky'eyetabyemu

Omukono gw'omujulizi______________________

______________________ Olunaku/omwezi/omwaka

To be completed by the researcher: I confirm that the individual has given consent freely.

Name of researcher: ______________________ Date:|__|__|/|__|__|/|__|__|__|__|

dd / mm / yyyy

Signature: ______________________

UVRI-RESEARCH ETHICS COMMITTEE
VALID UNTIL
★ 2 8 MAY 2023 ★
APPROVED
P. O. BOX 49, ENTEBBE (U)

UVRI-RESEARCH ETHICS COMMITTEE
VALID UNTIL
★ 2 6 MAY 2023 ★
APPROVED
P. O. BOX 49, ENTEBBE (U)
