# Supplementary material for: Menstrual health interventions, schooling, and mental health problems among Ugandan students (MENISCUS): study protocol for a school-based cluster-randomised trial
Source: Trials. 2022 Sep 7;23:759. doi: 10.1186/s13063-022-06672-4 (PMC9449307; doi:10.1186/s13063-022-06672-4)
Supplement: Supplementary file 2 — Additional file 2. [file 13063_2022_6672_MOESM2_ESM.zip › AN2CB6~1R1.PDF]

MRC Medical Research Council | Uganda Virus Research Institute | LONDON SCHOOL of HYGIENE & TROPICAL MEDICINE

Information and consent form for parents of females in the MENISCUS to be provided with the menstrual cup

**Project title:**	Menstrual health interventions, schooling and mental health symptoms among Ugandan students (MENISCUS): a school-based cluster-randomised trial
**Funder:**	UK Joint Global Health Trials (Medical Research Council-Department for International Development-Wellcome Trust) Grant # MR/V005634/1
**Research Site:**	Wakiso and Kalungu Districts<br>C/o MRC/UVRI and LSHTM Uganda Research Unit.<br>Plot 51-59, Nakiwogo Road<br>P O Box 49, Entebbe, Uganda<br>Tel: +256(0) 417 704000; (0)312 262910/1; (0)702 438487
**Principal Investigators:**	**1. Prof Helen Weiss,**<br>Professor of Epidemiology and Director of the MRC Tropical Epidemiology Group, London School of Hygiene and Tropical Medicine (LSHTM), UK<br>*Email: helen.weiss@lshtm.ac.uk*
**Local Principal Investigators:**	**2. Prof Janet Seeley**<br>Professor of Anthropology and Health, London School of Hygiene and Tropical Medicine (LSHTM), UK<br>and Head of Social Science Programme, MRC/UVRI and LSHTM Uganda Research Unit<br>*Email: janet.seeley@lshtm.ac.uk*
**Trial Manager:**	Dr. Catherine Kansiime,<br>MRC/UVRI and LSHTM Uganda Research Unit<br>*Email:Catherine.Kansiime@mrcuganda.org*

Summary (What you should know about this exploratory study):

- The aim of the sub-study is to assess whether provision of a menstrual cup as part of a school-based intervention focused on improving management of menstrual periods improves education, health and well-being outcomes among girls in secondary school in Wakiso and Kalungu districts in Uganda.
- This document explains the purpose of this study and asks whether you agree for your daughter to participate in the study. We will explain what your daughter will be asked to do if you and she agree to participate.
- Your daughter's participation is completely voluntary. You or your daughter have the right for your daughter to take part in the study or to agree to take part now and change your mind later.
- Whatever you decide will not affect your daughter's regular healthcare and support.
- Please review this form carefully. Ask any questions before you make a decision.

You will be given a copy of this form to keep.

RESEARCH ETHICS COMMITTEE
VALID UNTIL
★ 26 MAY 2023 ★
APPROVED
P. O. BOX 49, ENTEBBE (U)

Part I: Information about this study

Introduction

You have already agreed for your daughter to take part in the MENISCUS trial, which is led by scientists at the London School of Hygiene & Tropical Medicine, MRC/UVRI and LSHTM Uganda Research Unit, with our partner WoMena Uganda.

We would now like to explain an additional, optional part of the study. This is the offer for your daughter to receive a menstrual cup, and have training on how to use it. If your daughter receives the cup or not, she will still receive the reusable pads and all parts of the intervention.

We invite your daughter to be part of this research. It is optional for you to choose whether or not you want her to participate in this research. We have received permission to conduct this research from your child's/dependent's school administration, the district, the Ministry of Education and Sports, and the Research Ethics Committees of the UVRI, LSHTM and Uganda National Council of Science and Technology (UNCST).

If you agree to allow your daughter to participate, we shall ask your daughter for approval as well. Both of you have to approve before she can be involved.

Please feel free to ask us questions now or later using our contact information which is indicated below. We will take time to explain to you.

Purpose

The purpose of the main MENISCUS study is to see whether a health promotion intervention in secondary schools improves menstrual health (i.e. how girls manage their periods safely and confidently).

The purpose of offering girls the menstrual cup is to see whether providing an additional method of menstrual hygiene management, in addition to the re-usable pads, is helpful for girls to manage their menstruation.

Selection

We request your daughter to participate in this research because she is a female secondary school student starting Form 2 in one of 60 schools chosen for this study. We are seeking your consent because you are the parent/guardian of your daughter.

Voluntary Participation

It is optional for your daughter to participate in this research. You or she can choose to say no. That decision shall not affect any services that she receives at the secondary school and/or health facilities. You can ask as many questions as you like and we shall be available to answer them. You don't have to decide today. You can think about it and tell us what you decide later.

It is optional for you to choose whether or not you want your daughter to participate in this part of research.

Your daughter can take part in all other parts of the study but not receive the menstrual cup.

UVRI-RESEARCH ETHICS COMMITTEE
VALID UNTIL
28 MAY 2023
APPROVED
P. O. BOX 49, ENTEBBE (U)

Procedures
As part of the menstrual kit that your daughter will receive, she will also receive a menstrual cup (called the Ruby Cup). This is an alternative to pads to manage periods. The cup is a soft silicone cup which is folded and inserted into the vagina to collect the menstrual fluids and can be worn during the day and overnight without concern of leaking. The cup must be emptied once it is full, then it will be rinsed and reinserted again if necessary. Most girls/women need only to empty it in the morning and again in the evening. By experience, your daughter will know how often to empty it according to her specific needs.

Your daughter will be asked to participate in a session led by a teacher or peer who has been trained by an expert trainer in menstrual health, the menstrual cup and re-usable pads. They will show your daughter how to use the menstrual cup and re-usable pads, and will discuss any concerns you might have about this. your daughter will be asked to use the menstrual cup and/or re-usable pads for the next year, until she is in S3, if she feels comfortable doing so. At the end of this time, your daughter may be asked about her experience using the cup and re-usable pads. If she is experiencing any problems using the cup, she will be able to discuss it with the trained team leader.

Risks and discomfort: Is this bad or dangerous for you?
Menstrual cups have been used widely in many countries, including Uganda, and have few health risks. your daughter will be taught how to insert it. Once the cup is inserted, most girls find it painless, even if they have never had sexual intercourse. However, your daughter may feel some discomfort when she inserts and removes it (especially the first few times), and she may also feel embarrassed or may be afraid of inserting the cup into the vagina.

There is a risk of an allergy to the material that is used to make the menstrual cup (silicone), but this is very rare. If your daughter experiences pain, burning, irritation, inflammation in the genital area or discomfort during urination, she must remove the cup and contact the school study nurse or study coordinator immediately. The menstrual cup has been used by many millions of women around the world, and there has been one documented case of a woman becoming ill with toxic shock syndrome after using a menstrual cup called the DivaCup. It causes high fever, flu-like symptoms, dizziness, and can rapidly lead to severe illness. Toxic shock syndrome is very rare, and it has not been reported with RubyCup, which is the cup that we are offering. However, if your daughter experiences these symptoms during her periods, then she should remove the menstrual cup immediately, go to the local health clinic for a check-up, and contact our study coordinator by phone. If she has suffered from toxic shock syndrome previously, it is recommended not to use any internal form of sanitary protection, including menstrual cups or tampons. Your daughter will be taught how to clean the cup. There is a risk of infection or irritation if the cup is not cleaned properly.

Benefits: Is there anything good that happens to you?
Your daughter is being offered a choice of menstrual products. She may prefer using the cup to the pads.

Reimbursements: Will you get anything for being in the research?
You will be given 10,000 shillings to compensate for your time and effort. Your daughter will not be paid to take part in this research. However, she will be given a pen, a hardcover note book and a soft drink to compensate for your time and effort.

Confidentiality: Is anybody going to know about this?
We will not tell other people that your daughter was involved in this research. We shall not share personal information that identifies your daughter to anyone who does not work in this research. Any information about your daughter will have a study number on it instead of your name. However, your daughter's data may be seen by auditors.

VRI RESEARCH ETHICS COMMITTEE
VALID UNTIL
26 MAY 2023
APPROVED
P. O. BOX 49, ENTEBBE (U)

Sharing the Findings: Will you be told the study results?
When this research is completed, we shall inform you and your daughter about the results obtained. Then we shall share the research results with authorities at the school, municipal and national levels, including what we have learnt.

Afterwards, we will be telling other people, scientists, health workers and others, what we found. We will do this by writing and sharing reports and by going to meetings with people who are interested in this work. The research findings will be published in international science journals and electronic websites so that other people may learn from us. However, the results will never be reported in a way that allows anyone except members of the research team to know what your daughter specifically told us or any of the individual results we obtained from her. Data may be made available in the public domain via the London School of Hygiene and Tropical Medicine data repository. This means that it may be used for further analyses. All data will be anonymised i.e. it cannot be linked to your daughter.

Who to contact: Who can you talk to or ask questions about this study?
You can ask us questions now or later by telephone, e-mail, post or at the physical addresses indicated on the assent/consent form to be given to you. If you are nearby, you can come and see us.

You can ask us questions now or later by telephone, e-mail, post or at the physical addresses indicated on the assent/consent form to be given to you. If you are nearby, you can come and see us.

You can contact any of the following about this research:

a) Dr. Catherine Kansiime, MENISCUS Trial Project Lead
Email: Catherine.Kansiime@mrcuganda.org; Phone number +256 702438487

If you have any questions, complaints or concerns about your rights as a person involved in this research, please contact: UVRI Research Ethics Committee: Phone number +256 0414 321962 or +256 716 321962.

UVRI-RESEARCH ETHICS COMMITTEE
VALID UNTIL
★ 26 MAY 2023 ★
APPROVED
P. O. BOX 49, ENTEBBE (U)

PART 2: PARENTAL CONSENT (VERSION 1.3, NOVEMBER 2021)

By signing below I consent for my daughter to participate in the sub-study as described above, including for:

- My daughter to receive a reusable menstrual cup and training on how to use it
- All anonymised data collected to be used as part of the research and shared with other researchers

My questions concerning this study have been answered by ……………………………….

Please read each question below	Please circle all you agree with:	
Have you read (or had read to you) information about this project?	Yes	No
Has somebody else explained this project to you?	Yes	No
Do you understand what this project is about?	Yes	No
Have you had any questions answered in a way you understand?	Yes	No
Do you understand that it is ok to stop taking part at any time?	Yes	No
Are you happy for your daughter to take part in this study? [CONSENT]	Yes	No

Student name: ______________________________ School ID: |__|__|__|

Name of Parent/Guardian/
Authorized Delegate: ___________________________ Signature: ______________________

Date of consent: |__|__|/|__|__|/|__|__|__|__|
dd / mm / yyyy

An Informed Assent Form will be completed by your child, if they also agree.

If literacy challenged : A literate witness must sign (if possible, this person should be selected by the participant and should have no connection to the research team). Literacy challenged parents/guardians should include their thumb print as well.

Print name of witness________________________ **AND left thumb print of participant**

Signature of witness _________________________

Date _____________________ **Day/month/year**

To be completed by the researcher: I confirm that the individual has given consent freely.

Name of researcher: ______________________ Date:|__|__|/|__|__|/|__|__|__|__|
dd / mm / yyyy

Signature: ___________________

[illegible] RESEARCH ETHICS COMMITTEE
VALID UNTIL
26 MAY 2023
APPROVED
P. O. BOX 49, ENTEBBE (U)

UVRI-RESEARCH ETHICS COMMITTEE
VA[illegible]
26 MAY 2023
A[illegible]D
P. O. BOX 49, ENTEBBE (U)
