# Supplementary material for: Menstrual health interventions, schooling, and mental health problems among Ugandan students (MENISCUS): study protocol for a school-based cluster-randomised trial
Source: Trials. 2022 Sep 7;23:759. doi: 10.1186/s13063-022-06672-4 (PMC9449307; doi:10.1186/s13063-022-06672-4)
Supplement: Supplementary file 2 — Additional file 2. [file 13063_2022_6672_MOESM2_ESM.zip › AN35CD~1R1.PDF]

**Ekiwandiiko ky'abazadde b'abaana abawala abanawebwa akakopo akakozesebwa mu nsonga z'ekikyala mu kunonyereza kwa MENISCUS.**

| | |
|---|---|
| **Project title:** | Menstrual health interventions, schooling and mental health symptoms among Ugandan students (MENISCUS): a school-based cluster-randomised trial |
| **Funder:** | UK Joint Global Health Trials (Medical Research Council-Department for International Development-Wellcome Trust) Grant # MR/V005634/1 |
| **Research Site:** | Wakiso and Kalungu Districts<br>C/o MRC/UVRI and LSHTM Uganda Research Unit.<br>Plot 51-59, Nakiwogo Road<br>P O Box 49, Entebbe, Uganda<br>Tel: +256(0) 417 704000; (0)312 262910/1; (0)702 438487 |
| **Principal Investigators:** | **1. Prof Helen Weiss,**<br>Professor of Epidemiology and Director of the MRC Tropical Epidemiology Group, London School of Hygiene and Tropical Medicine (LSHTM), UK<br>*Email: helen.weiss@lshtm.ac.uk* |
| **Local Principal Investigators:** | **2. Prof Janet Seeley**<br>Professor of Anthropology and Health, London School of Hygiene and Tropical Medicine (LSHTM), UK<br>and Head of Social Science Programme, MRC/UVRI and LSHTM Uganda Research Unit<br>*Email: janet.seeley@lshtm.ac.uk* |
| **Trial Manager:** | Dr. Catherine Kansiime,<br>MRC/UVRI and LSHTM Uganda Research Unit<br>*Email:Catherine.Kansiime@mrcuganda.org* |

**Mu bufunze (By'olina okumanya ku kunoonyereza kuno):**

- Ekigendererwa ky'okunonyereza kuno kwekuzula obanga okugaba akakopo akakozesebwa mu nsonga z'ekikyala nga emu kungeri abaana abawala jebasobola okubeera obulunji nga bali mu nsonga z'ekikyala kiyamba kukusoma, awamu n'embela yobulamu mu baana abawala mu mamasomero ga secondary e Wakiso ne Kalungu mu Uganda.
- Ekiwandiiko kino kinnyonnyola ekigendererwa ky'okunoonyereza kuno ne muwala wo kyanasabibwa okukola singa gwe naye munabamukirizza okukwetabamu
- Okw'etaba kw'omwanawo mu kunoonyereza kuno kwa kyeyagalire. Dembe lyo gwe n'omwana wo okukwetabamu, oba okukwetabamu oluvannyuma nemukuvaamu.
- Kyonna ky'anaaba asazeewo tekijja kukosa ngeri muwala wo jafunamu bujjanjabi wadde obuyambi n'akatono.
- Soma ekiwandiiko kino n'obwegendereza era obuuze ekibuuzo kyonna ky'oyagala nga tonasalawo.

**Ojja kuweebwa kopi ku kiwaandiiko kino**

UVRI RESEARCH ETHICS COMMITTEE
26 MAY 2023
APPROVED
P. O. BOX 49, ENTEBBE (U)

**Part I: Ebikwata ku kunoonyereza kuno**
**Enyanjula:**

Wamaze dda okusalawo nti muwala wo yetabe mu kunonyereza kwa MENISCUS - trial, okukulembedwa bana science okuva ku London School of Hygiene & Tropical Medicine, MRC/UVRI ne LSHTM Uganda Research Unit, nga bakolaganira wamu ne WoMena Uganda.

Tugenda kukunyonyola ekitundu ekyennjawulo mukunonyeleza kuno, ekikwata ku muwalawo okuwebwa akakopo akakozesebwa mu nsonga z'ekikyala awamu n'okusosomesebwa enkozesa yako. Muwala wo bwanaba afunye oba nga tafunye kakopo kano ajja genda maaso okufuna paadi ezoozebwa ne ziddamu ne zikozesebwa (re-usable pads) nebintu ebilala byona ebinaba bigabibwa mukunonyereza kuno.

Tuyise muwalawo okwetaba mukunonyereza kuno, oliwaddembe okusalawo oba muwalawo yetaba mukunonyereza kuno oba nedda. Okunoonyereza kuno kwa kiriziddwa akulira e somero muwala wo gyasomera, district, ekitongole ky'ebyenjigiriza n'emizannyo awamu n'akakiiko ka UVRI akavunanyizibwa ku kunoonyereza, Akakiiko akavunanyizibwa ku kunonyereza aka London School of Hygiene and Tropical Medicine, awamu n'akakiiko ka Uganda akavunanyizibwa ku Sayansi ne Tekinologiya (UNCST).

Bwonakkiriza muwalawo okwetaba mu kunoonyereza kuno naye tujja kumusaba akkirize. Mwembi muyina okukiriza nga tanetabamu.

Oli waddembe okutubuuza ekibuuzo kyonna ky'oyagala kati oba oluvannyuma ng'oyita ku email ne namba z'esimu eziragiddwa wa manga era tujja kutwala obuvunaanyizibwa tukunnyonnyole otegeere.

**Ekigendererwa:**
Ekigendererwa ky'okunoonyereza kwa MENISCUS kwekulaba oba nga okutumbula eby'obulamu mu masomero ga secondary kiyamba ku nsonga za bakyala (okugeza, engeri abaana abawala jebasobola okubeera obulunji nga bali mu nsonga z'ekikyala)

Ekigendelerwa kyokuwa abaana abawala akakopo akakozesebwa mu nsonga z'ekikyala kwekuzula oba engeri endala abaana abawala jebasobola okubeera obulunji nga bali mu nsonga z'ekikyala) nga ogaseko okukozesa paadi ezoozebwa ne ziddamu ne zikozesebwa (re-usable pads) kiyamba abaana abawala okubera obulungi nga bali munsonga z'ekikyala.

## Okulonda
Tusaba muwala wo okwetaba mu kunoonyereza kuno kubanga muyizi Muwala (Female student) owa siniya 2 mu limu ku masomero 60 agaalondeddwa okukoleramu okunonyereza kuno. Tukusaba olukusa kuba ggwe muzadde/alina obuvunaanyizibwa (Guardian) okkirize omwanawo

**Okwetaba mu kunoonyereza kuno kwa kyeyagalire:**

Okwetaba mu kunoonyereza kuno kwa kyeyagalire. Ggwe oba omwana muli baddembe okugaana. Okusalawo obuteegatta mu kunoonyereza kuno tekijja kukosa bye mulina kufuna ku somero wadde ewajjanjabirwa wonna. Oli wa ddembe okutubuuza ebibuuzo byonna era tuli beetegefu okubyanukula. Osobola obutasalawo kati, oli waddembe okusooka okukirowoozaako n'otubuulira oluvannyuma ky'onooba osazeewo.

Oli waddembe okukkiriza oba obutakiriza muwala wo obuteteba mukino ekintu kyokunonyereza kuno.

UVRI-RESEARCH ETHICS COMMITTEE
VALID UNTIL
28 MAY 2023
APPROVED
P. O. BOX 49, ENTEBBE (U)

Muwala wo asobola okwetaba mubintu by'okunonyereza byona ebirala naye natafuna kakopo.

**Emitendera:**

Muwalawo ajja kuweebwa ettu ly'ebikozesebwa mu nsonga z'ekikyala nga mulimu akakopo akakozesebwa mu nsonga z'ekikyala (Ruby Cup) mu kifo kya paadi. Akakopo kano kagonvu nga kazingibwa ne kasonsenkebwa mu bukyala ne kagendamu omusaayi gw'ensonga z'ekikyala. Omuntu asobola okukambala obudde bw'emisana bwonna n'ekiro nga tekajjudde kuyiika, bwe kajjula okajjayo mu bukyala n'oyiwa ebirimu, n'okooza balunji n'okazzaayo. (Abawala/Abakyala abasinga bakajjayo ku makya n'olweggulo). Bwanaatandika okakozesa ajja kulaba ekinaamwanguyira okusinziira ku bwetaavubwe.

Muwalawo ajja kusabibwa okwetaba mu musomo ogunaakubirizibwa omusomesa oba omu ku bayizi banne anaaba atendekeddwa abakugu mu nsonga z'ekikyala, mu nsonga z'akakopo akakozesebwa mu nsonga z'ekikyala, ne paadi ezoozebwa ne ziddamu ne zikozesebwa. Bajja ku mulaga engeri y'okukozesaamu Akakopo ne paadi ezo era bajja ku munnyonnyola buli kyanaaba ayagala okumanya ku nsonga eno. Ajja kusabibwa okukozesa paadi ezoozebwa ne ziddamu ne zikozesebwa / Akakopo okumala omwaka ogunaddako mpaka nga ali mu senior 3 bwanaaba takirinaako buzibu. ajja kubitegeeza ku abakulembera abakola ku kunoonyereza (Team leader).

**Obutyabaga n'okuteteganyizibwa: Kino kibi oba kya bulabe eri muwalawo?**

Obukopo buno bukozesebwa abakyala banji nnyo mu nsi eziwerako nga ne Uganda mweri era tebulina nyo bulabe singa buba bukozesedwa mubutufu bwabwo. Akakopo bwe kamala okusonsekebwayo abawala abasinga obungi tebalina bulumi bwe bafuna wadde nga tewegattangako na basajja. Naye era muwalawo ayinza obutawulira bulunji ng'akateekayo oba ng'akajjayo (naddala emirundi egisooka), era ayinza okuwulira ensonyi oba okutya ng'akasonseka mu bukyala.

Waliwo akatyabaga k'okufuna allergy nga eva ku matiriyo ekozebwa mu kukola akakopo kano (silicone), naye kino tekitera kubaawo. Muwalawo singa aba afunye obulumi oba okusiyiibwa mubitundu bye eby'ekyama oba obulumi bwona nga afuyisa, aba alina okujayo akakopo n'okwogera amangu eri omusawo oba akulira okunonyereza kunno.

Obukopo buno bukozesebwa abakyala banji nnyo mu nsi eziwerako era waliwo esonga emu yooka ewandikibwako eyomukyala eyafuna embeela eyitibwa (toxic shock syndrome) bweyakozesa akakopo akayitibwa DivaCup. Embera eno eleta omusujja, senyiga, kamunguluze ela osobola okulwala. Toxic shock syndrome tetela kubelawo ate ela tewulilwangako nga eva kukozesa (Ruby cup) akokopo ketugenda okugaba, naye singa muwalawo aba afunye obubonero bunno nga alimunsonga z'ekikyala ayina okugenda mumaso okujayo akakopo n'okwogera amangu eri omusawo oba akulira okunonyereza kunno. Muwala wo bwaba yali afunyeko ku toxic shock syndrome emabega akubirizibwa obutakoseza kintu kyona nga kiyingila mu bukyalabwe okugeza akakopo ne tampon. Muwalawo ajja kusomesebwa engeri y'okwozaamu akakopo. Wayinza okubalukawo akatyabaga kokukwatibwa obulwadde singa akakopo kaba tekalongosedwa bulungi.

**Okuganyurwa (benefits): Waliwo engeri muwalawo gyanaganyurwamu?**

Muwalawo aweredwa ebikozesebwa munsonga z'ekikyala eby'enjawulo. Ayinza okusalawo okukozesa akakopo oba padi.

**Okusasulwa: Muwalawo anaasasulwa olw'okwetaba mu kunoonyereza kuno?**



RESEARCH ETHICS COMMITTEE
VALID UNTIL
26 MAY 2023
APPROVED
P. O. BOX 49, ENTEBBE (U)

Ojja kusasulwa omutwalo gumu 10000 olwobudde bwo nekawefube gw'onaba otaddemu. Muwalawo tajja kusasulwa olw'okwetaba mu kunoonyereza kuno, mpozzi ajja kuweebwayo ka peni n'akatabo akeddiba eggumu ko n'akokunywa akagonvu olw'obuddebwe ne kaweefube gw'anaaba ataddemu.

**Emmizi (Confidentiality): Ebintu bino binaamanyibwako abantu abalala?**

Tewali gwe tujja kubuulirako nti muwalawo yeetabye mu kunoonyereza kuno. Tewali muntu yenna atakola mu kunoonyereza kuno gwe tujja kubuulirako ku bimukwatako era tujja kuba tukozesa namba (study number) mu kifo ky'e linnyalye. Wabula amawulire muwala wo gatuwadde gayinza okulabibwako ba auditors.

**Okutegeezebwa ebinaazuulibwa mu kunoonyereza: Onootegeezebwa ebinaazuulibwa mu kunoonyereza kuno?**

Okunoonyereza kuno nga kuwedde gwe ne muwalawo mujja kutegeezebwa ebinaaba bizuuliddwa era tujja kubitegeeza n'abakulira essomero lino ko aba Munisipaali ne Gwanga lyonna okutwalira awamu omuli nebyo byetunaba tuyize.

Oluvanyuma tujja kubitegeeza n'abantu abalala omuli ba Nasayansi, abakola ku by'obulamu, n'abantu abalala. Kino tujja kikola nga tuyita mu kuwandiika zi alipoota, n'okusisinkana bonna be kikwatako. Ebinaava mu kunoonyereza kuno era bya kuteekebwa mu butabo (journals) bwa sayansi obw'ensi yonna ko n'emikutu ja intaneti abantu abalala basobole okutuyigirako. Ebivudde mukunonyereza kuno era biyinza okutekebwa ku mukutu gwa London School of Hygiene and Tropical medicine abantu abalala gyebayinza okubisanga. Kino kitegeza nti tuyinza okudamu okwekenenya ebinaba bivudde mukunonyereza naye nga tewali ngeri yonna mu kwogera ebinaava mu kunoonyereza kuno muwalawo bye yatubuulira ng'omuntu we bijja kulabikira.

**Okwebuuza: Ani gw'oyinza okw'ogerako naye oba okubuuza ebikwata ku kunoonyereza kuno?**

Oli waddembe okubuuza ekibuuzo kyonna kati oba je bujja ng'oyita ku simu oba ku e-mail oba okujja ku MRC/UVRI kwe nnyini n'otulaba mu buntu. Osobola okutuukirira omu ku bantu bano wa manga

a) Dr. Catherine Kansiime, Akulila okunonyereza kwa MENISCUS-Trial
*Email:* Catherine.Kansiime@mrcuganda.org; Enamba y'esimu +256 702438487

Bwoba olina ekibuuzo oba okwemulugunya ku ddembelyo ku by'okwetabakwo mu kunoonyereza kuno tuukirira akakiiko ka UVRI akalondoola n'okulabirira okunonyereza ku simu
+256 0414 321962 oba +256 716 321962.

UVRI-RESEARCH ETHICS COMMITTEE
VALID UNTIL
26 MAY 2023
APPROVED
P. O. BOX 49, ENTEBBE (U)

**EKITUNDU 2: OKUKKIRIZA KW'ABAZADDE (VERSION 1.3 NOVEMBER 2021)**

Nga ntekako omukono, nzikiriza muwala wange okwetaba mu kitundu ekimu ekiri mu kunonyereza kuno nga mulimu;

- Muwala wange okufuna akakopo n'okusomesebwa engeri y'okukakozesamu
- Ebinava mukunonyereza kuno okukozesebwa n'okutegezako abanonyereza abalala naye nga ebikwata ku muwala wange tebija kumanyibwa.

Ebibuuzo byange ebikwata ku kunoonyereza kuno byanukuddwa ……………………………….

| Soma ebibuzo bino wamanga | Circle byokiriza | |
|---|---|---|
| Osomye/ bansomedde ebikwata ku kunonyereza kuno? | Yee | Nedda |
| Waliwo omuntu omulala okunyonyonde ebikwata ku kunonyereza kuno? | Yee | Nedda |
| Otegedde ebikwata ku kunonyereza kuno? | Yee | Nedda |
| Ebibuzo byo bididwamu mungeri gyotegera? | Yee | Nedda |
| Otegedde nti oli waddembe okuva mu kunoonyereza kuno wonna woba oyagalidde | Yee | Nedda |
| Oli musanyusa olwa muwalawo okwetaba mukunonyereza kuno? | Yee | Nedda |

Student name: ________________________ School ID: |__|__|__|

Wandiika (Print) Amannya g'omuzadde/avunaanyizibwa ku mwana/Eyaweebwa

Obuvunaanyizibwa ___________________________________

Omukono gwo'omuzadde(signature)_______________________

Date of consent (IDATE): |__|__|/|__|__|/|__|__|__|__|
dd / mm / yyyy

**Omwanawo naye ajja kuyisibwa mu kiwandiiko ky'okukkiriza bwanaaba naye akkirizza.**

**Omuzadde atasobola kusoma na kuwandiika**: Omuzade nga tasobola kusoma na kuwandiika omujulizi yateekako omukono, omujulizi ono bwe kiba kisobose omuzadde ya mwerondera era tasaanye kuba nga alina akakwate konna n'abakola ku kunoonyereza kuno.

Erinnya ly'omujulizi _________________________________

Omukono (Signature) g'omujulizi___________________________

Ennaku z'omwezi_______________________________Day/month/year

Ekyinkumu ky'omuzadde

**To be completed by the researcher**
I confirm that the individual has given consent freely.

Name of researcher: ______________________ Date:|__|__|/|__|__|/|__|__|__|__|
dd / mm / yyyy

Signature: ____________________

UVRI RESEARCH ETHICS COMMITTEE
[illegible]
26 MAY 2023
APPROVED
P. O. BOX 49, ENTEBBE (U)
