# Supplementary material for: Menstrual health interventions, schooling, and mental health problems among Ugandan students (MENISCUS): study protocol for a school-based cluster-randomised trial
Source: Trials. 2022 Sep 7;23:759. doi: 10.1186/s13063-022-06672-4 (PMC9449307; doi:10.1186/s13063-022-06672-4)
Supplement: Supplementary file 2 — Additional file 2. [file 13063_2022_6672_MOESM2_ESM.zip › AN556A~1R1.PDF]

MRC/UVRI and LSHTM Uganda Research Unit

MRC Medical Research Council | Uganda Virus Research Institute | LONDON SCHOOL of HYGIENE &TROPICAL MEDICINE

Ekiwandiiko ekisaaba abazadde oba abavunanyizibwa ku baana abawala abanawebwa akakopo akakozesebwa mu nsonga z'ekikyala mu kunonyereza kwa MENISCUS.

**Project title:**	Menstrual health interventions, schooling and mental health symptoms among Ugandan students (MENISCUS): a school-based cluster-randomised trial
**Funder:**	UK Joint Global Health Trials (Medical Research Council-Department for International Development-Wellcome Trust) Grant # MR/V005634/1
**Research Site:**	Wakiso and Kalungu Districts<br>C/o MRC/UVRI and LSHTM Uganda Research Unit<br>Plot 51-59, Nakiwogo Road<br>P O Box 49, Entebbe, Uganda<br>Tel: +256(0) 417 704000; (0)312 262910/1; (0)702 438487
**Principal Investigators:**<br><br><br><br>**Local Principal Investigator:**	**1. Prof Helen Weiss,**<br>Professor of Epidemiology and Director of the MRC Tropical Epidemiology Group, London School of Hygiene and Tropical Medicine (LSHTM), UK<br>*Email: helen.weiss@lshtm.ac.uk*<br>**2. Prof Janet Seeley**<br>Professor of Anthropology and Health, London School of Hygiene and Tropical Medicine (LSHTM), UK<br>and Head of Social Science Programme, MRC/UVRI and LSHTM Uganda Research Uni<br>*Email: janet.seeley@lshtm.ac.uk*
**Trial Manager:**	Dr. Catherine Kansiime,<br>MRC/UVRI and LSHTM Uganda Research Unit<br>*Email:Catherine.Kansiime@mrcuganda.org*

Mubufunze (By'Olina okutegera ku kunonyereza kuno):

- Ekigendererwa ky'okunonyereza kuno kwekumanya oba nga ettu lya MENISCUS linayambako mu kulongosa eby'okusoma, obubonero obulabirwako eby'obulamu ebikwata ku by'obwongo, okutumbula engeri abaana abawala jebasobola okubeera obulungi nga bali mu nsonga z'ekikyala awamu n'embeera y'obulamu bwabwe mu masomero ga siniya mu Wakiso ne Kalungu Districts mu Uganda.

- Ekiwandiiko kino kinnyonnyola ekigendererwa ky'okunoonyereza kuno n'omwana wo ky'anasabibwa okukola singa onooba omukkirizza okukwetabamu.

- Okwetaba kw'omwanawo mu kunoonyereza kuno kwa kyeyagalire. Dembe lyo gwe n'omwana wo okukwetabamu, Oba okukwetabamu oluvannyuma nemukuvaamu.

- Kyonna kyonaaba asazeewo tekijja kukosa ngeri muwala wo jafunamu bujjanjabi wadde obuyambi n'akatono.

UVRI-RESEARCH ETHICS COMMITTEE
[illegible]
26 MAY 2023
APPROVED
P. O. BOX 49, ENTEBBE (U)

- Soma ekiwandiiko kino n'obwegendereza era obuuze ekibuuzo kyonna ky'oyagala nga tonasalawo.

Ojjakuweebwa kopi ku kiwaandiiko kino

Ekitundu Ekisooka: Ebikwata ku kunoonyereza kuno

Enyanjula:

Okunonyereza kwa MENISCUS, kukulembedwamu bana science okuva ku MRC/UVRI ne London School of Hygiene & Tropical Medicine Entebbe, nga bakolaganira wamu ne WoMena Uganda. Tukola Okunoonyereza kuno okulungamya amasomero ga Siniya okuzuula engeri ezisoboka ez'okuyambamu abaana abawala okubeera abalamu n'okubeera ku somero obulungi nga bali mu nsonga z'ekikyala. Twafunye olukusa okukola Okunoonyereza kuno okuva eri abatwala e Somero lino, okuva ku disitirikiti, okuva mu kitongole ky'ebyenjigiliza n'emizanyo n'okuva mu bukiiko obulondoola okunoonyereza obwa MRC/UVRI ne LSI-IT M wamu ne National Council of Science and Technology.

Tukusaba (okiriize) muwalawo yetabe mukunonyereza kuno, oliwaddembe okusalawo oba muwalawo yetaba mukunonyereza kuno oba nedda. Oli waddembe okutubuuza ekibuuzo kyonna ky'oyagala kati Oba oluvannyuma ng'oyita ku email ne namba z'esimu eziragiddwa wa manga era tujja kutwala obuvunaanyizibwa tukunnyonnyole otegeere.

Ekigendererwa:
Ekigendererwa ky'okunoonyereza kwa MENISCUS kwekulaba oba nga okutumbula eby'obulamu mu masomero ga secondary kiyamba ku nsonga z’ekikyala (okugeza, engeri abaana abawala jebasobola okubeera obulunji nga bali mu nsonga z'ekikyala). Twagala okumanya oba nga kinaayambako mu kulongosa eby'okusoma, eby'obulamu mubaana abawala awamu n'okumanya kwa baana abalenzi kubikwata kusonga za bakyala. Okunonyereza kunno bwekunaba kuvudemu ebirungi, kujja kutongozebwa mumasomera amalala mu Uganda.

Okulonda
Omwana wo alondeddwa okwetaba mukunonyereza kuno kubanga muyizi mu limu ku masomero amakuumi assatu (30) agaalondeddwa okufuna ettu lya MENISCUS era ayinza okuba nga ali mu kibiina/group erondoola emirimu egikolebwa mu somero egikwatagana ku kutumbula engeri abaana abawala jebasobola okubeera obulungi nga bali mu nsonga z’ekikyala.

Okwetaba mu kunoonyereza kuno kwa kyeyagalire:
Okwetaba mu kunoonyereza kuno kwa kyeyagalire. Ggwe Oba omwana muli baddembe okugaana. Okusalawo obuteegatta mu kunoonyereza kuno tekijja kukosa bye mulina kufuna ku somero wadde ewajjanjabirwa wonna. Oli wa ddembe okutubuuza ebibuuzo byonna era tuli beetegefu okubyanukula. Osobola obutasalawo kati, oli waddembe okusooka okukirowoozaako n'otubuulira oluvannyuma ky'onooba osazeewo. Gwe n’omwana wo muli ba ddembe okuva mu kunonyereza kuno obudde bwonna.

Emitendera:
Okunonyereza kuno kukolebwa wakati w'omwaka 2021 ne 2023 mu masomero nkaaga (60) aga secondary agaalondeddwa mu Wakiso ne Kalungu Districts. Ku gano, amasomero amakuumi assatu (30) aganaba galondeddwa, bajja kufuna ettu lya MENISCU nga mwemuli okusomesebwa ku nkyukakyuka ezibawo nga omwana avubuka n'ensonga z'ekikyala, okuterezamu ku kabuyonjo

UVRI-RESEARCH ETHICS COMMITTEE
VALID UNTIL
26 MAY 2023
APPROVED
P.O. BOX 49, ENTEBBE (U)

z'essomero era bajakuba n'omukisa okwetaba mu katemba oba emizanyo nga bikwatagana n'ensonga z'ekikyala, n'okufuna ettu ly'ebikozesebwa munsonga zekikyala wamu n'okusomesebwa kungeri ezikendeza/y'okukendeeza ku bulumi nga omuwala ali munsonga z'ekikyala.

Nga ekimu kubinabera mu ttu ly'ebikozesebwa mu nsonga z'ekikyala muwala wo lyanafuna, olina eddembe okumukiriza okufuna/okuwebwa akakopo akakozesebwa mu nsonga z'ekikyala (Ruby Cup). Akakopo kakozesebwa mu nsonga z'ekikyala mu kifo kya paadi. Akakopo kano kagonvu nga kazingibwa ne kasonsenkebwa mu bukyala ne kagendamu omusaayi gw'ensonga z'ekikyala. Omuntu asobola okukambala obudde bw'emisana bwonna n'ekiro nga tekajjudde kuyiika, bwe kajjula okajjayo mu bukyala n'oyiwa ebirimu, n'okooza balunji n'okazzaayo. (Abawala/Abakyala abasinga bakajjayo ku makya n'olweggulo). Bwanaatandika okakozesa aja kulaba ekinaamwanguyira okusinziira ku bwetaavubwe. (Bwosalawo muwalawo obutawebwa/obutafuna kakopo akakozesebwa mu nsonga z'ekikyala, era ajjakufuna ebintu byonna ebirala ebiri mu ttu ly'ebikozesebwa mu nsonga z'ekikyala.

Muwalawo aja kusabibwa okwetaba mu musomo ogunaakubirizibwa abakugu mu nsonga z'ekikyala, n'engeri y'okukozesamu akakopo akakozesebwa mu nsonga z'ekikyala. Baja ku mulaga engeri y'okukozesaamu Akakopo era bajja ku munnyonnyola buli kyanaaba ayagala okumanya ku nsonga eno. Aja kusabibwa okukozesa paadi ezoozebwa ne ziddamu ne zikozesebwa / Akakopo okumala omwaka ogunaddako bwanaaba takirinaako buzibu. Bwanaba alina obuzibu bwafunye mu kukozesa ekimu ku biri mu ttu ly'ebikozesebwa nga ali munsong z'ekikyala. Ajakusabibwa okubitegeeza ku abakulembera abakola ku kunoonyereza (Team leader)., abakugu abakola ku kunoonyereza oba dokita akwasaganya ensonga mukunoonyereza kuno.

Obutyabaga n'okuteteganyizibwa: Kino kibi Oba kya bulabe eri muwalawo?
Obukopo buno bukozesebwa abakyala banji nnyo mu nsi eziwerako nga ne Uganda mweri era tebulina nyo bulabe Singa buba bukozesedwa mubutufu bwabwo. Akakopo bwe kamala okusonsekebwayo abawala abasinga obungi tebalina bulumi bwe bafuna wadde nga tewegattangako na basajja. Naye era muwalawo ayinza obutawulira bulunji ng'akateekayo oba ng'akajjayo (naddala emirundi egisooka), era ayinza okuwulira ensonyi oba okutya ng'akasonseka mu bukyala.

Waliwo akatyabaga k'okufuna allergy nga eva ku matiriyo ekozebwa mu kukola akakopo kano (silicone), naye kino tekitera kubaawo. Muwalawo Singa aba afunye obulumi oba okusiyiibwa mubitundu bye eby'ekyama oba obulumi bwona nga afuyisa, aba alina okujayo akakopo n'okwogera amangu eri omusawo oba akulira okunonyereza kunno.

Obukopo buno bukozesebwa abakyala banji nnyo mu nsi eziwerako era waliwo esonga emu yooka ewandikibwako eyomukyala eyafuna embeela eyitibwa (toxic shock syndrome) bweyakozesa akakopo akayitibwa DivaCup. Embera eno eleta omusujja, senyiga, kamunguluze ela osobola okulwala. Toxic shock syndrome tetela kubelawo ate ela tewulilwangako nga eva kukozesa (Ruby cup) akokopo ketugenda okugaba, naye Singa muwalawo aba afunye obubonero bunno nga alimunsonga z'ekikyala ayina okugenda mumaso okujayo akakopo n'okwogera amangu eri omusawo oba akulira okunonyereza kunno. Muwala wo bwaba yali afunyeko ku toxic shock syndrome emabega akubirizibwa obutakoseza kintu kyona nga kiyingila mu bukyalabwe okugeza akakopo ne tampon. Muwalawo aja kusomesebwa engeri y'okwozaamu akakopo. Wayinza okubalukawo akatyabaga kokukwatibwa obulwadde Singa akakopo kaba tekalongosedwa bulungi.

Okuganyurwa (benefits): Waliwo engeri muwalawo gyanaganyurwamu?
Muwalawo ajakuwebwa ebikozesebwa munsonga z'ekikyala eby'enjawulo. Ayinza okusalawo okukozesa akakopo Oba padi.

UVRI-RESEARCH ETHICS COMMITTEE
VALID UNTIL
20 MAY 2023
APPROVED
P. O. BOX 49, ENTEBBE (U)

Okwetaba kw'omwanawo mu kunoonyereza kuno kusobola okutuyamba, okuyamba amasomero, amalwaliro, n'abavunaanyizibwa ku byenjigiriza okuzuula amawulire (information) n'obuweereza (services) bye mwetaaga. Tusuubira nga kino kijja kuyamba be kikwatako okukola ku byetaago byabwe mu ngeri esinga okuba ennunji eyo jebujja.

Okusasulwa: Muwalawo anaasasulwa olw'okwetaba mu kunoonyereza kuno?
Muwalawo tajja kusasulwa olw'okwetaba mu kunoonyereza kuno. Wabula, oja kusasulwa omutwalo gurnu 10,000 olwobudde bwo nekawefube gw'onaba otaddemu.

Emmizi (Confidentiality): Ebintu bino binaamanyibwako abantu abalala?
Tewali gwe tujja kubuulirako nti muwalawo yeetabye mu kunoonyereza kuno. Tewali muntu yenna atakola mu kunoonyereza kuno gwe tujja kubuulirako ku bimukwatako era tujja kuba tukozesa namba (study number) mu kifo ky'e linnyalye. Wabula amawulire muwala wo gatuwadde gayinza okulabibwako ba auditors.

Okutegeezebwa ebinaazuulibwa mu kunoonyereza: Onootegeezebwa ebinaazuulibwa mu kunoonyereza kuno?
Okunoonyereza kuno nga kuwedde gwe ne muwalawo mujja kutegeezebwa ebinaaba bizuuliddwa era tujja kubitegeeza n'abakulira essomero lino ko aba Munisipaali ne Gwanga lyonna okutwalira awamu ornuli nebyo byetunaba tuyize.

Oluvanyuma tujja kubitegeeza n'abantu abalala ornuli ba Nasayansi, abakola ku by'obulamu, n'abantu abalala. Kino tujja kikola nga tuyita mu kuwandiika zi alipoota, n'okusisinkana bonna be kikwatako. Ebinaava mu kunoonyereza kuno era bya kuteekebwa mu butabo (journals) bwa sayansi obw'ensi yonna ko n'emikutu ja intaneti abantu abalala basobole okutuyigirako. Ebivudde mukunonyereza kuno era biyinza okutekebwa ku mukutu gwa London School of Hygiene and Tropical medicine abantu abalala gyebayinza okubisanga. Kino kitegeza nti tuyinza okudamu okwekenenya ebinaba bivudde mukunonyereza naye nga tewali ngeri yonna mu kwogera ebinaava mu kunoonyereza kuno muwalawo bye yatubuulira ng'omuntu we bijja kulabikira.

Okwebuuza: Ani gw'oyinza okw'ogerako naye Oba okubuuza ebikwata ku kunoonyereza kuno?

Oli waddembe okubuuza ekibuuzo kyonna kati Oba je bujja ng'oyita ku simu Oba ku e-mail Oba okujja ku MRC/UVRI kwe nnyini n'otulaba mu buntu. Osobola okutuukirira omu ku bantu bano wa manga

a) Dr. Catherine Kansiime, Akulila okunonyereza kwa MENISCUS-Trial
Email: Catherine.Kansiime@mrcuganda.org; Enamba y'esimu +256 702438487

Bwoba olina ekibuuzo Oba okwemulugunya ku ddembelyo ku by'okwetabakwo mu kunoonyereza kuno tuukirira akakiiko ka UVRI akalondoola n'okulabirira okunonyereza ku simu +256 0414 321962 oba +256 716 321962.

EKITUNDU 2: OKUKKIRIZA KW'ABAZADDE (VERSION 1.0 JANUARY 2022)

Nga ntekako omukono, nzikiriza muwala wange okwetaba mu kitundu ekimu ekiri mu kunonyereza kuno nga mulimu;

- Okufuna ettu lye bikozesebwa munsonga z'ekikyala n'okusomesebwa engeri yokulikozesamu/ engeri ettu jelikozesebwamu.

UVRI-RESEARCH ETHICS COMMITTEE
VALID UNTIL
26 MAY 2023
APPROVED
P.O. BOX 49, ENTEBBE (U)

- Ebinava mukunonyereza kuno okukozesebwa n'okutegezako abanonyereza abalala naye nga ebikwata ku muwala wange tebija kumanyibwa.

Ebibuuzo byange ebikwata ku kunoonyereza kuno byanukuddwa ....

Soma ebibuzo bino wamanga	Sazaako byokiriza	
Osomye/ bansomedde ebikwata ku kunonyereza kuno?	Yee	Nedda
Waliwo omuntu omulala okunyonyonde ebikwata ku kunonyereza kuno?	Yee	Nedda
Otegedde ebikwata ku kunonyereza kuno?	Yee	Nedda
Ebibuzo byo bididwamu mungeri gyotegera?	Yee	Nedda
Otegedde nti oli waddembe okuva mu kunoonyereza kuno wonna woba oyagalidde?	Yee	Nedda
Oli musanyusa olwa muwalawo okwetaba mukunonyereza kuno?	Yee	Nedda

Erinya ly'eyataba mu kunonyereza: ______________________________ School ID: |__|__|__|

Erinya ly'omuzadde/omukuza: ______________________________

Omukono gw'omuzadde/omukuxa: ____________________

Enaku z'omwezi (dd/mm/yyyy): |__|__|/|__|__|/|__|__|__|__|

Bw'aba tasobola kusoma na kuwandiika: Omujulizi asobola okusoma n'okuwandiika alina okutekako omukono. (bwekiba kisoboka, omuntu ono alina okulondebwa eyetabyemu era talina kuba nankolagana yonna n'abakola kukunonyereza kuno). Omuzadde/alabirira omwana atasobola kusoma nakuwandiika atekeko ekyenkumu kye.

Erinnya ly'omujulizi ______________________ **ne** **Ekyenkumu ky'eyetabyemu**

Omukono gw'omujulizi____________________

Ennaku z'omwezi ______________________ **Olunaku/omwezi/omwaka**

Omunonyereza ajjuzemu: Nkakasa nti omuntu ono asabidwa olukusa era akiriza okwetaba mu kunonyereza kuno nga yeyagalidde.

Erinya ly'anonyereza: ______________________ Enaku z'omwezi:|__|__|/|__|__|/|__|__|__|__|

dd / mm / yyyy

Omukono: __________________

UVRI-RESEARCH ETHICS COMMITTEE
VA[illegible]
26 MAY 2023
[illegible]
P. O. BOX 49, ENTEBBE (U)
