# Supplementary material for: Menstrual health interventions, schooling, and mental health problems among Ugandan students (MENISCUS): study protocol for a school-based cluster-randomised trial
Source: Trials. 2022 Sep 7;23:759. doi: 10.1186/s13063-022-06672-4 (PMC9449307; doi:10.1186/s13063-022-06672-4)
Supplement: Supplementary file 2 — Additional file 2. [file 13063_2022_6672_MOESM2_ESM.zip › AN607D~1R1.PDF]

MRC Medical Research Council | Uganda Virus Research Institute | LONDON SCHOOL of HYGIENE & TROPICAL MEDICINE

Information and consent form for teachers of students in secondary schools participating in the MENISCUS trial formative interviews

**Project title:**	Menstrual health interventions, schooling and mental health symptoms among Ugandan students (MENISCUS): a school-based cluster-randomised trial
**Funder:**	UK Joint Global Health Trials (Medical Research Council-Department for International Development-Wellcome Trust) Grant # MR/V005634/1
**Research Site:**	Wakiso and Kalungu Districts<br>C/o MRC/UVRI Uganda Research Unit on AIDS<br>Plot 51-59, Nakiwogo Road<br>P O Box 49, Entebbe, Uganda<br>Tel: +256(0) 417 704000; (0)312 262910/1; (0)752 731733
**Principal Investigator:**<br><br><br><br>**Local Principal Investigator:**	**1. Prof Helen Weiss,**<br>Professor of Epidemiology and Director of the MRC Tropical Epidemiology Group, London School of Hygiene and Tropical Medicine (LSHTM), UK<br>*Email: helen.weiss@lshtm.ac.uk*<br>**2. Prof Janet Seeley**<br>Professor of Anthropology and Health, London School of Hygiene and Tropical Medicine (LSHTM), UK<br>and Head of Social Science Programme, MRC/UVRI<br>*Email: janet.seeley@lshtm.ac.uk*
**Trial Manager:**	Dr. Catherine Kansiime,<br>MRC/UVRI and LSHTM Uganda Research Unit<br>*Email:Catherine.Kansiime@mrcuganda.org*
**Project Manager**	Ms Sophie Belfield<br>WoMena Uganda Research and Innovation Manager<br>*Email:* Sophie.belfield@womena.dk

Summary (What you should know about this study):

- The aim of the study is to assess whether a school-based menstrual health intervention improves education, health and well-being outcomes among girls in secondary school in Wakiso and Kalungu districts in Uganda.
- This document explains the purpose of this study, and what you will be asked to do if you agree to participate
- Your participation is completely voluntary. You have the right to not take part in the study or to agree to take part now and change your mind later.
- Whatever you decide will not affect your regular support and work at your school.
- Please review this form carefully. Ask any questions before you make a decision.

You will be given a copy of this form to keep.

UVRI RESEARCH ETHICS COMMITTEE
VALID UNTIL
26 MAY 2023
APPROVED
P. O. BOX 49, ENTEBBE (U)

Part I: Information about this study

Introduction
The MENISCUS trial is led by the MRC/UVRI Unit in Entebbe and the London School of Hygiene and Tropical Medicine in United Kingdom, in collaboration with our partner WoMena Uganda.

We are carrying out research to guide secondary schools to identify practical ways of helping girls to become and stay healthier and complete studying at school through improved management of menstrual periods.

We invite you to be part of this research. It is optional for you to choose whether or not you want to participate in this research. We have received permission to conduct this research from your school administration, the district, the Ministry of Education and Sports, and the Research Ethics Committees of the UVRI, LSHTM and Uganda National Council of Science and Technology (UNCST).

Please feel free to ask us questions now or later using our contact information which is indicated below. We will take time to explain to you.

Purpose
The purpose of the MENISCUS trial is to refine and introduce a health promotion intervention in schools, to promote menstrual hygiene for girls and to learn whether the package is likely to improve educational attainment, mental health symptoms, menstrual management and quality of life outcomes among girls in secondary school in Uganda.

Selection
We request you to participate in this research because you are a teacher of secondary school students in a school that has previously/currently had a menstrual health intervention.

Voluntary Participation
It is optional for you to participate in this research. You can choose to say no and you can choose to stop participating at any time. That decision shall not affect any support or services that you receive at the secondary school. You can ask as many questions as you like and we shall be available to answer them.

Procedures
We request you to participate in a focus group discussion with up to 10 other staff members, facilitated a researcher (180 minutes).

Procedures
This research will take place between February and June 2022 in two secondary schools in Wakiso and Kalungu Districts and is a part of the MENISCUS trial which is taking place from 2021-2023 in a separate 60 identified secondary schools in Wakiso and Kalungu Districts. The research has been formally approved by the: Uganda National Council for Science and Technology (UNCST); UVRI Research and Ethics Committee (UVRI REC); the Ethics Committee of the London School of Hygiene and Tropical Medicine (LSHTM) and by the authorities in the Departments of Education and Sports and of Health for the local government.

1) <u>Focus group discussion (~180 minutes)</u>:
You will be requested to have an group interview with a trained researcher, to ask about the school timetables and procedure after school closures, to get feedback on a training of trainers for teachers on menstrual health, and to understand how groups (such as health groups) work in your school. The

UVRI-RESEARCH ETHICS COMMITTEE
VALID UNTIL
2 6 MAY 2023
APPROVED
P. O. BOX 49, ENTEBBE (U)

discussion will take place at an agreed venue or within the school premises. The researcher will record paper notes on the discussion. The information recorded is confidential, and no one else except the researchers or other ethical eligible person(s) such as the sponsor or ethics review committees with regulated access to the notes will be allowed to read the notes.

Risks and discomfort: Is the study bad or dangerous for you?
We will ask you to share your views on resources which talk about menstrual management at school. You may feel uncomfortable talking about some of the topics.

Benefits: Is there anything good that happens to you from participating?
Your participation is likely to help us, the schools, health facilities and the education and health authorities to find out more about your health information and service needs. We hope that these will help all the relevant people to meet those needs better in the future. Taking part in this research may also remind you to think deeply about the girls' health and future.

Reimbursements: Will you receive anything for being in the study?
You will be given a meal and transport refund to compensate for your time and effort.

Confidentiality: Is anybody going to know about this?
We will not tell other people that you were involved in this research. We shall not share personal information that identifies you to anyone who does not work in this research. Any information about you will have a study number on it instead of your name. However, your data may be seen by auditors.

Sharing the Findings: Will you be told the study results?
When this research is completed, we shall inform you about the results obtained. Then we shall share the research results with parents/guardians, authorities at the school, municipal and national levels, including what we have learnt.

Afterwards, we will be telling other people, scientists, health workers and others, what we found. We will do this by writing and sharing reports and by going to meetings with people who are interested in this work. The research findings will also be published in international science journals and electronic websites so that other people may learn from us. However, the results will never be reported in a way that allows anyone except members of the research team to know what you specifically told us or any of the individual results we obtained from you. Data may be made available in the public domain via the London School of Hygiene and Tropical Medicine data repository. This means that it may be used for further analyses. All data will be anonymised i.e. it cannot be linked to you.

Who to Contact: Who can you talk to or ask questions about this study?
You can ask us questions now or later by telephone, e-mail, post or at the physical addresses indicated on the assent/consent form to be given to you. If you are nearby, you can come and see us. You can contact the following about this research:

Dr. Catherine Kansiime
MENISCUS trial Project Lead
Email: catherine.kansiime@mrcuganda.org
Phone number +256 702438487

If you have any questions, complaints or concerns about your rights as a person involved in this research, please contact: UVRI Research Ethics Committee: Phone number +256 0414 321962 or +256 716 321962

UVRI RESEARCH ETHICS COMMITTEE
V[illegible]
26 MAY 2023
APPROVED
P. O. BOX 49, ENTEBBE (U)

PART II: Consent form (Version 1.0 January 2022)

By signing below I consent to participate in the study as described above, including:

- To participate in a focus group discussion
- For all anonymised data collected to be used as part of the research and shared with other researchers

My questions concerning this study have been answered by ………………………………..

Please read each question below	Please circle all you agree with:	
Have you read (or had read to you) information about this project?	Yes	No
Has somebody else explained this project to you?	Yes	No
Do you understand what this project is about?	Yes	No
Have you had any questions answered in a way you understand?	Yes	No
Do you understand that it is ok to stop taking part at any time?	Yes	No
Are you happy to take part in this study? [CONSENT]	Yes	No

School ID: |__|__|__|

Name of participant: ________________________________

Signature of Participant: ____________________

Date of consent (dd/mm/yyyy): |__|__|/|__|__|/|__|__|__|__|

UVRI-RESEARCH ETHICS COMMITTEE
VALID UNTIL
26 MAY 2023
APPROVED
P. O. BOX 49, ENTEBBE (U)

To be completed by the researcher: I confirm that the individual has given consent freely.

Name of researcher: ______________________ Date:|__|__|/|__|__|/|__|__|__|__|

dd / mm / yyyy

Signature: ___________________
