# Supplementary material for: Menstrual health interventions, schooling, and mental health problems among Ugandan students (MENISCUS): study protocol for a school-based cluster-randomised trial
Source: Trials. 2022 Sep 7;23:759. doi: 10.1186/s13063-022-06672-4 (PMC9449307; doi:10.1186/s13063-022-06672-4)
Supplement: Supplementary file 2 — Additional file 2. [file 13063_2022_6672_MOESM2_ESM.zip › AN7C76~1R1.PDF]

MRC/UVRI and LSHTM Uganda Research Unit

MRC Medical Research Council | Uganda Virus Research Institute | LONDON SCHOOL of HYGIENE &TROPICAL MEDICINE

Head Teacher information and consent form for school participation in the MENISCUS trial

**Project title:**	Menstrual health interventions, schooling and mental health symptoms among Ugandan students (MENISCUS): a school-based cluster-randomised trial
**Funder:**	UK Joint Global Health Trials (Medical Research Council-Department for International Development-Wellcome Trust) Grant # MR/V005634/1
**Research Site:**	Wakiso and Kalungu Districts<br>C/o MRC/UVRI and LSHTM Uganda Research Unit.<br>Plot 51-59, Nakiwogo Road<br>P O Box 49, Entebbe, Uganda<br>Tel: +256(0) 417 704000; (0)312 262910/1; (0)702 438487
**Principal Investigators:**<br><br><br><br><br><br>**Local Principal Investigator:**	**1. Prof Helen Weiss,**<br>Professor of Epidemiology and Director of the MRC Tropical Epidemiology Group, London School of Hygiene and Tropical Medicine (LSHTM), UK<br>*Email: helen.weiss@lshtm.ac.uk*<br>**2. Prof Janet Seeley**<br>Professor of Anthropology and Health, London School of Hygiene and Tropical Medicine (LSHTM), UK<br>and Head of Social Science Programme, MRC/UVRI and LSHTM Uganda Research Unit.<br>*Email: janet.seeley@lshtm.ac.uk*
**Trial Manager:**	Dr. Catherine Kansiime,<br>MRC/UVRI and LSHTM Uganda Research Unit<br>*Email:Catherine.Kansiime@mrcuganda.org*

Summary (What you should know about this study):

- The aim of the study is to assess whether a school-based menstrual health intervention improves education, health and well-being outcomes among girls in secondary school in Wakiso and Kalungu districts in Uganda.
- This document explains the purpose of this study and asks whether you agree for your school to participate in the study. We will explain what is involved if your school decides to take part.
- Your participation is completely voluntary. You have the right to decide whether to take part and if you agree now you can change your mind later. Your decision will not affect any of your rights or the rights of your school.
- Please review this form carefully. Ask any questions you would like.

You will be given a copy of this form to keep.

UVRI-RESEARCH ETHICS COMMITTEE
VALID UNTIL
26 MAY 2023
APPROVED
P. O. BOX 49, ENTEBBE (U)

Part I: Information about this study

Introduction

The MENISCUS trial is led by scientists at the London School of Hygiene & Tropical Medicine, MRC/UVRI and LSHTM Uganda Research Unit (MUL), with our partner WoMena Uganda.

We are carrying out research to guide secondary schools to identify practical ways of helping girls to become and stay healthier and complete studying at school through improved management of menstrual periods. We have received permission to conduct this research the district, the Ministry of Education and Sports, and the Research Ethics Committees of the UVRI, LSHTM and Uganda National Council of Science and Technology (UNCST).

We invite your school to be part of this research. You can choose whether or not you want your school to participate. If you agree, we will also ask for consent from the parents of female S2 students who are directly involved in the research study as well as for permission (assent) from the students themselves. Please feel free to ask us any questions now or later using our contact information which is indicated below.

Purpose

The purpose of the MENISCUS study is to see whether a health promotion intervention in secondary schools improves menstrual health (i.e. how girls manage their periods safely and confidently). We want to learn whether the package is likely to improve education, health and well-being outcomes among girls, and menstrual health knowledge and attitudes towards periods among boys. If the intervention is successful, it could be introduced in other schools in Uganda.

Selection

Your school has been selected because it is a secondary school in Wakiso or Kalungu district that has both male and female S2 students, and your school previously participated in the MENISCUS Rapid Assessment study to assess other eligibility criteria relating to school size and water, sanitation, and hygiene (WASH) facilities.

Voluntary Participation

It is optional for your school to participate in this research. You can choose to say no and that decision will not affect any of your or your school's rights. You can ask as many questions as you like and we shall be available to answer them. If you agree to take part now, you can change your mind later.

Procedures

There will be 60 schools in the trial. Of these, 30 will be randomly selected in a public ceremony to receive the MENISCUS intervention in 2022. The other half will have the opportunity to receive the MENISCUS intervention in 2023.

If you agree for your school to take part, you will need to allow access to the school facilities, students, and registers to enable us to conduct parental consent, surveys, trainings, and to deliver the intervention. You will need to liaise with MUL to facilitate parental consent and all data collection activities; with the Uganda National Examinations Board (UNEB) to facilitate the educational assessment; and with WoMena Uganda to support the delivery of the MENISCUS intervention. By agreeing to take part, you agree for your school to participate in the following procedures:

<u>Consent and assent</u>: We will seek parental consent from the parents/guardians of all female students starting S2 in 2022 ahead of students' direct participation in the intervention or data collection activities. We will also seek consent and parental consent from additional staff and students selected to participate in interviews or group discussions. We will ask your school to provide contact information for students' parents and support

UVRI-RESEARCH ETHICS COMMITTEE
VALID UNTIL
26 MAY 2023
APPROVED
P. O. BOX 49, ENTEBBE (U)

informational meetings held at the school. Students will be asked for their assent ahead of participating in the individual-level elements of the study.

Research data collection: To evaluate the impact of the intervention, we will collect school- and student-level data at the start ("baseline") and end ("endline") of the study as well as process data throughout the intervention year.

All female students with parental consent as well as a sample of male S2 students will complete a questionnaire at the beginning and end of the study if they provide assent. At the end of the study, we will ask female students to self-collect vaginal swabs and a urine sample to test for urinary tract infection, bacterial vaginosis, and candida. A random sample of female S2 students will complete a daily diary on school attendance and menstruation for approximately 3 months. We may also ask some students and school staff to participate in individual interviews or focus group discussions.

Participating schools will be required to allow UNEB to administer educational assessments covering recently taught curricula at baseline and endline on dates set by UNEB and to further allow the research team to use these data. Individual-level assessment results will not be available to your school nor to students.

At baseline and endline, a trained research team member will conduct a survey of the school WASH facilities, with some photos, lasting about 1-2 hours on one day. You will not be told of the day in advance, so that they researcher can observe the facilities on a typical school day. We may ask you or another school staff member to answer some additional questions about the WASH facilities and resources available.

If you choose to participate and your school is selected to receive the intervention during the study, we will require your assistance in collecting process data using simple log-books, such as documenting attendance at puberty education trainings and drama skit rehearsals, and completing log-books on analgesics redeemed by students involved in the study. These logs should be completed with accuracy and shared with the research team in a timely manner. A research team member may also conduct unannounced school visits (up to monthly) to monitor the intervention and observe the status of the WASH facilities.

Randomisation: Following the baseline data collection, a ceremony will be held with school and district representatives. At the ceremony, the participating schools will be randomly divided into two groups: the intervention arm, which will receive the MENISCUS intervention package during the trial in 2022, and the control arm, which will receive Government materials and be offered the MENISCUS intervention after the evaluation is completed. Schools have an equal chance of being in either group and are requested to not share information about the intervention package with schools in the opposite group until the end of the study.

Intervention delivery: Schools allocated to the intervention arm and those choosing to receive the intervention package after the end of the study will be required to support WoMena Uganda in delivering the intervention. The intervention includes:

i. Formation of a Menstrual Health Action Group comprised of staff, parents, and students. This group should be recognised as a school committee (or similar) and provided space and time to meet.

ii. Puberty education training for all relevant teachers, lasting a total of approximately three days. You should allow relevant teachers to participate and support them in delivering puberty education sessions at school following the training.

iii. Performance of a drama skit about menstrual health. Participating schools are asked to identify an appropriate person to receive an allowance to lead the group, allow time and space for rehearsals, and organise a performance for students, parents, and community members.

iv. Workshops for teachers and senior students to become 'menstrual health trainers' and deliver training sessions on menstrual health, menstrual products, and puberty to male and female S2 students. Participating schools will be required to put forward potential trainers and facilitate their delivery of the sessions. At these trainings, female students with parental consent and assent will receive a menstrual health kit containing reusable pads and optional menstrual cup.

UVRI-RESEARCH ETHICS COMMITTEE
VALID UNTIL
26 MAY 2023
APPROVED
P. O. BOX 49, ENTEBBE (U)

v. Small improvements to school WASH facilities (door locks, sanitary bins, toilet paper cages, liquid soap and drums for water) which should be maintained to the school's by identified focal persons.

vi. Provision of analgesics (paracetamol and ibuprofen) provided by MUL to S2 female students. These should be distributed by the school nurse or other designated teacher who are available to be trained by the study team and who will also need to track stock and distribution using the MENISCUS voucher system and log book.

Risks and discomfort: Is the study bad or dangerous?
There is a risk that the study activities may cause some disruption on the days of the baseline and endline surveys and intervention training sessions.

Benefits: Is there anything good that happens from participating?
Participating schools will benefit from improvements to the WASH facilities. Your school will also receive training for teachers and senior students to support menstrual health. We will provide a school-level summary of students' performance on the UNEB assessments. Overall, the MENISCUS intervention has the potential to improve girls' health and wellbeing, and may reduce school absence and dropout. All students may benefit from a potential reduction in stigma around menstruation and a school environment that is more supportive of menstrual health.

Confidentiality: Is anybody going to know about this?
All information collected will be kept confidential and stored securely. We will not share any personal information that identifies your school to anyone who does not work on this study. Some data may be seen by auditors. Your school will not be named in any publication or report. Any information about your school will have a study number on it instead of a name.

Sharing the Findings: Will you be told the study results?
When this research is completed, we will inform the school community about results obtained from the trial. The results will never be reported in a way that allows anyone except members of the research team to know what you specifically told us or any of the individual results we obtained from you. We will also shall share the research results with authorities at the school, district and national levels, including what we have learnt.

Afterwards, we will be telling other people, scientists, health workers and others, what we found. We will do this by writing and sharing reports and by going to meetings with people who are interested in this work. The research findings will be published in international science journals and electronic websites so that other people may learn from us. Data may also be made available in the public domain via the London School of Hygiene and Tropical Medicine. This means that it may be used for further analyses. All data will be anonymised i.e. it cannot be linked to your school.

Who to Contact: Who can you talk to or ask questions about this study?
You can ask us questions now or later by telephone, e-mail, post or at the physical addresses indicated on the assent/consent form given to you. If you are nearby, you can come and see us.

You can contact any of the following about this research

Dr. Catherine Kansiime, MENISCUS Trial Manager
Email: Catherine.Kansiime@mrcuganda.org; Phone number +256 702438487

If you have any questions, complaints or concerns about your rights as a person involved in this research, please contact: UVRI Research Ethics Committee: Phone number +256 0414 321962 or +256 716 321962

UVRI-RESEARCH ETHICS COMMITTEE
VALID UNTIL
26 MAY 2023
APPROVED
P. O. BOX 49, ENTEBBE (U)

PART 2: HEAD TEACHER CONSENT (VERSION 1.0 JANUARY 2022)

By signing below, I consent for my school to participate in this study. I have been told about the study and understand what will take place. I understand my school will not be named in any report or publication, and that all anonymised data collected can be used and shared as part of this research.

My questions concerning this study have been answered by ....

Please read each question below	Please circle all you agree with:	
Have you read (or had read to you) information about this project?	Yes	No
Has somebody else explained this project to you?	Yes	No
Do you understand what this project is about?	Yes	No
Have you had any questions answered in a way you understand?	Yes	No
Do you understand that it is ok to stop taking part without any consequences?	Yes	No
Are you happy for your school to take part in this study?	Yes	No

Authorisation of school to participate in the MENISCUS study:

Name: ____________________________ Name of school: __________________________

Position (circle) : Head teacher / Deputy HT / Other____________________ School ID ____________

Signature: ___________________________ Date (dd/mm/yyyy): |__|__|/|__|__|/|__|__|__|__|

dd / mm / yyyy

If literacy challenged: A literate witness must sign (if possible, this person should be selected by the participant and should have no connection to the research team). Literacy challenged parents/guardians should include their thumb print as well.

Print name of witness_________________________ AND left thumb print of participant

Signature of witness __________________________

Date ________________________ (dd/mm/yyyy)

To be completed by the researcher: I confirm that the individual has given consent freely.

Name of researcher: _______________________ Date:|__|__|/|__|__|/|__|__|__|__|

dd / mm / yyyy

Signature: ____________________

UVRI-RESEARCH ETHICS COMMITTEE
VALID UNTIL
26 MAY 2023
APPROVED
P. O. BOX 49, ENTEBBE (U)

UVRI-RESEARCH ETHICS COMMITTEE
VA[illegible] UNTIL
26 MAY 2023
A[illegible]ED
P. O. BOX 49, ENTEBBE (U)
