# Supplementary material for: Menstrual health interventions, schooling, and mental health problems among Ugandan students (MENISCUS): study protocol for a school-based cluster-randomised trial
Source: Trials. 2022 Sep 7;23:759. doi: 10.1186/s13063-022-06672-4 (PMC9449307; doi:10.1186/s13063-022-06672-4)
Supplement: Supplementary file 2 — Additional file 2. [file 13063_2022_6672_MOESM2_ESM.zip › ANA15F~1R1.PDF]

MRC/UVRI and LSHTM Uganda Research Unit

MRC Medical Research Council | Uganda Virus Research Institute | LONDON SCHOOL of HYGIENE &TROPICAL MEDICINE

Information and assent form for students in the secondary schools invited to participate in the MENISCUS trial

**Project title:**	Menstrual health interventions, schooling and mental health symptoms among Ugandan students (MENISCUS): a school-based cluster-randomised trial
**Funder:**	UK Joint Global Health Trials (Medical Research Council-Department for International Development-Wellcome Trust) Grant # MR/V005634/1
**Research Site:**	Wakiso and Kalungu Districts<br>C/o MRC/UVRI and LSHTM Uganda Research Unit.<br>Plot 51-59, Nakiwogo Road<br>P O Box 49, Entebbe, Uganda<br>Tel: +256(0) 417 704000; (0)312 262910/1; (0)702 438487
**Principal Investigators:**	**1. Prof Helen Weiss,**<br>Professor of Epidemiology and Director of the MRC Tropical Epidemiology Group, London School of Hygiene and Tropical Medicine (LSHTM), UK<br>*Email: helen.weiss@lshtm.ac.uk*
**Local Principal Investigator:**	**2. Prof Janet Seeley**<br>Professor of Anthropology and Health, London School of Hygiene and Tropical Medicine (LSHTM), UK<br>and Head of Social Science Programme, MRC/UVRI and LSHTM Uganda Research Unit.<br>*Email: janet.seeley@lshtm.ac.uk*
**Trial Manager:**	Dr. Catherine Kansiime,<br>MRC/UVRI and LSHTM Uganda Research Unit<br>*Email:Catherine.Kansiime@mrcuganda.org*

Who to Contact: Who can you talk to or ask questions about this study?
You can ask us questions now or later by telephone, e-mail, post or at the physical addresses indicated above. If you are nearby, you can come and see us.

You can contact any of the following about this research:
Dr. Catherine Kansiime, MENISCUS Trial Project Lead
Email: Catherine.Kansiime@mrcuganda.org; Phone number +256 702438487

If you have any questions, complaints or concerns about your rights as a person involved in this research, please contact: UVRI Research Ethics Committee: Phone number +256 0414 321962 or +256 716 321962

UVRI RESEARCH ETHICS COMMITTEE
VALID UNTIL
26 MAY 2023
APPROVED
P. O. BOX 49, ENTEBBE (U)

PART 2: ASSENT (VERSION 1.1, APRIL 2021)

I have been asked to give consent to participate in the MENISCUS study. I have been given digital information concerning this study and I understand what will be required of me if I take part in this study.

The digital information about this study can be found here
https://www.lshtm.ac.uk/research/centres-projects-groups/meniscus#resources
or by contacting the researchers listed on the other page.

My questions concerning this study have been answered by ……………………………….

I understand that at any time, I may withdraw from this study without giving a reason. I agree to the possible use of my data being made available in the public domain via the London School of Hygiene and Tropical Medicine data repository. This means that it may be used for further analyses. The data will be anonymised i.e. it cannot be linked to me.

**Please read each question below**	**Please circle all you agree with:**	
Have you been given information about this project?	Yes	No
Has somebody else explained this project to you?	Yes	No
Do you understand what this project is about?	Yes	No
Have you had any questions answered in a way you understand?	Yes	No
Do you understand that it is ok to stop taking part at any time?	Yes	No
Are you happy to take part?	Yes	No

Study number (IDNO): |__|__|__|__|__|__|__|

Name of student: ______________________________

Signature of student: ____________________

Date of interview (IDATE): |__|__|/|__|__|/|__|__|__|__|
dd / mm / yyyy

To be completed by the researcher
Statement by the investigator/researcher:
I confirm that the individual has given assent freely.

Name of researcher: ______________________ Date:|__|__|/|__|__|/|__|__|__|__|
dd / mm / yyyy

Signature: ____________________

UVRI-RESEARCH ETHICS COMMITTEE
VALID UNTIL
26 MAY 2023
APPROVED
P. O. BOX 49, ENTEBBE (U)
