# Supplementary material for: Menstrual health interventions, schooling, and mental health problems among Ugandan students (MENISCUS): study protocol for a school-based cluster-randomised trial
Source: Trials. 2022 Sep 7;23:759. doi: 10.1186/s13063-022-06672-4 (PMC9449307; doi:10.1186/s13063-022-06672-4)
Supplement: Supplementary file 2 — Additional file 2. [file 13063_2022_6672_MOESM2_ESM.zip › ANA2F0~1R1.PDF]

MRC/UVRI and LSHTM Uganda Research Unit

MRC Medical Research Council — Uganda Virus Research Institute — London School of Hygiene & Tropical Medicine

Olupapula oluliko Amawulire agasaba Bazadde/Abavunaanyizibwa Ku Baana abalenzi Na'baakwasibwa Obuvunaanyizibwa Bw'okulabirira Abaana Nga bazadde Baabwe Tebaliiwo (Abakulu B'amasomero N'abalala) Okukkiriza Abaana ba Siniya Abalenzi Okwetaba mu kunoonyereza Kwa MENISCUS

**Project title:**	Menstrual health interventions, schooling and mental health symptoms among Ugandan students (MENISCUS): a school-based cluster-randomised trial
**Funder:**	UK Joint Global Health Trials (Medical Research Council-Department for International Development-Wellcome Trust) Grant # MR/V005634/1
**Research Site:**	Wakiso and Kalungu Districts<br>C/o MRC/UVRI and LSHTM Uganda Research Unit<br>Plot 51-59, Nakiwogo Road<br>P O Box 49, Entebbe, Uganda<br>Tel: +256(0) 417 704000; (0)312 262910/1. 0702 438487
**Principal Investigators:**	**1. Prof Helen Weiss,**<br>Professor of Epidemiology and Director of the MRC Tropical Epidemiology Group, London School of Hygiene and Tropical Medicine (LSHTM), UK<br>*Email: helen.weiss@lshtm.ac.uk*
**Local Principal Investigators:**	**2. Prof Janet Seeley**<br>Professor of Anthropology and Health, London School of Hygiene and Tropical Medicine (LSHTM), UK<br>and Head of Social Science Programme, MRC/UVRI and LSHTM Uganda Research Unit<br>*Email: janet.seeley@lshtm.ac.uk*
**Trial Manager:**	Dr. Catherine Kansiime,<br>MRC/UVRI and LSHTM Uganda Research Unit<br>*Email:Catherine.Kansiime@mrcuganda.org*

Mu bufunze (By'olina okumanya ku kunoonyereza kuno):

- Ekigendererwa ky'okunoonyereza kwa MENISCUS kwe kumanya oba nga kinayambako mu kulongosa eby'okusoma, obubonero obulabirwako eby'obulamu ebikwata kubwongo, okutumbula engeri abaana abawala jebasobola okubeera obulungi nga bali mu nsonga z'ekikyala awamu n'embeela y'obulamu bwabwe mu masomero ga siniya mu wakiso ne kalungu mu Uganda
- Ekiwandiiko kino kinnyonnyola ekigendererwa ky'okunoonyereza kuno ne ky'onasabibwaokukola singa onooba okkirizza omwanawo okukwetabamu.
- Okw'etaba kw'amutabani wo mu kunoonyereza kuno kwa kyeyagalire. Dembe lye okukwetabamu, oba okukwetabamu oluvannyuma n'akuvaamu.
- Kyonna ky'anaaba asazeewo tekijja kukosa ngeri jafunamu bujjanjabi wadde obuyambi.
- Soma ekiwandiiko kino n'obwegendereza era obuuze ekibuuzo kyonna ky'oyagala nga tonasalawo.

Ojja kuweebwa kopi ku kiwaandiiko kino

UVRI-RESEARCH ETHICS COMMITTEE
[illegible] UNTIL
28 MAY 2023
APPROVED
P. O. BOX 49, ENTEBBE (U)

Enyanjula (Introduction):
Okunoonyereza kwa MENISCUS ku kulembeddwamu ekitongole kya MRC/UVRI ne Tendekero lya London School of Hygiene and Tropical Medicine (LSHTM) nga bakolerera wamu n'ekitongole kya WOMENA Uganda.

Tukola Okunoonyereza kuno okulungamya amasomero ga Siniya okuzuula engeri ezisoboka ez'okuyambamu abaana abawala okubeera abalamu n'okubeera ku somero obulungi nga bali mu nsonga z'ekikyala.

Tukusaba okkirize omwanawo okwetaba mu kunoonyereza kuno. Ddembe lyo okumukkiriza oba obutamukkiriza. Twafunye olukusa okukola Okunoonyereza kuno okuva eri abatwala e Somero lino, okuva ku disitirikiti, okuva mu ministry y'ebyenjigiliza n'emizanyo n'okuva mu bukiiko obulondoola okunoonyereza obwa MRC/UVRI ne LSHTM.

Bw'onamukkiriza okwetaba mu kunoonyereza kuno, n'omwana tujja kumusaba akkirize.

Oli waddembe okutubuuza ekibuuzo kyonna ky'oyagala kati oba oluvannyuma ng'oyita ku email ne namba z'esimu eziragiddwa wa manga era tujja kutwala obuvunaanyizibwa tukunnyonnyole otegeere.

Ekigendererwa (Purpose):
Ekigendererwa ky'okunoonyereza kwa MENISCUS kwe kulaba oba nga enkola yokutumbula eby'obulamu mu mumasomero ga siniya enayambako mu kulongosa ensonga z'ekikyala (engeri abaana abawala jebasobola okubeera obulungi nga bali mu nsonga z'ekikyala) n'okumanya oba nga kinaayambako mu kulongosa eby'okusoma, eby'obulamu mubaana abawala awamu n'okumanya kwa baana abalenzi kubikwata kusonga za bakyala. Okunonyereza kunno bwekunaba kuvudemu ebirungi, kujja kutongozebwa mumasomera amalala mu Uganda.

Okulonda (Selection):
Tusaba mutabani wo okwetaba mu kunoonyereza kuno kubanga muyizi Mulenzi (male student) owa siniya 2 mu limu ku masomero enkaaga agaalondeddwa okukoleramu okunonyereza. Tukusaba kuba ggwe muzadde oba alina obuvunaanyizibwa (Guardian) kumwana ono.

Okwetamu kwakyeyagalile:
Okwetaba mu kunoonyereza kuno kwa kyeyagalire. Ggwe oba omwana muli baddembe okugaana. Okusalawo obuteegatta mu kunoonyereza kuno tekijja kukosa gwe ne famileyo bye mulina kufuna ku somero wadde ewajjanjabirwa wonna. Oli wa ddembe okutubuuza ebibuuzo byonna era tuli beetegefu okubyanukula. Osobola obutasalawo kati, oli waddembe okusooka okukirowoozaako n'otubuulira oluvannyuma ky'onooba osazeewo. Oli waddembe okukkiriza omukulu w'essomero oba omuntu yenna gw'oyagala okuteeka Omukono ku biwaandiiko ebikkiriza omwanawo okwetaba mu kunoonyereza kuno ku lulwo singa onooba toosobole kubaawo.

Emitendera
Okunonyereza kuno kwetabidwamu amasomero nkaaga (60) nga amakumi assatu (30) kugo aganaba galondedwa bajja kufuna ettu lya MENISCUS. Mu masomero gano amakumi assatu(30), abayizi mu siniya 2 kutandikwa y'omwaka 2022 bajakusomsebwa ku nkyukakyuka ezibawo nga omwana avubuka, ensonga z'ekikyala n'okuterezamu ku kabuyonjo z'essomero.

Ettu lino lijakugabibwa mu masomero mumwaka gwa 2022 gwonna. Ate ago amasomero aganaba tegafunye ettu lino, bajja kuba n'omukisa okufuna ettu lyelimu mu 2023.

UVRI RESEARCH ETHICS COMMITTEE
VALID UNTIL
26 MAY 2023
APPROVED
P. O. BOX 49, ENTEBBE (U)

Tukusaba okkirize mutabani wo okwetaba mu kukubaganya ebirowoozo okw'amu oba okwa sekinoomu . Bajakuyambibwako abavubuka abakola kukunonyereza kuno abanabera bayanyuddwa ku masomero era bajakutambulanga nebibakwattako (Identity cards). Mutabani wo ajakusabibwa okuwa endowooza ye ku ttu lya MENISCUS. Okukubaganya ebirowoozo kwa kubeera ku Somero era kujja kwatibwa ku butambi era obutambi obwo bwa kusibirwa mu kabada ku UVRI. Ebinaakwatibwa ku butambi bya kuumibwa nga bya kyama era tewali ajja ku biwulirako okujjako abakola ku kunoonyereza n'abalala abakkirizibwa mu mateeka agafuga okunoonyereza nga abajja okubissa mu buwandiike (Transicribers), abatadde ensimbi mu mulimu guno ko n'obukiiko obulondoola n'okulabirira okunonyereza. Era tewali linnya lya muntu yenna liggya kw'ogerwako.

Obutyabaga n'okuteteganyizibwa: Kino kibi oba kya bulabe eri mutabani wo?
Mutabani wo ayinza obutawulira mirembe oba bulungi nga ayogera ku nsonga ezimu. Mutabani wo ajakuwebwa omukisa okubuuza ebibuuzo byona n'okukubaganya ebirowoozo kwebyo eby'ogerwa ku nsonga mu bakyala.

Okuganyurwa (benefits): Waliwo engeri muwalawo gyanaganyurwamu?
Asobola okuganyulwa mw'ebyo ebiyinza okukolelwa okunonyeleza gamba ng'okutumbula omutindo gwa kabuyonjo z'esomero. Era okwetabaakwe mu kunoonyereza kuno kusobola okutuyamba, okuyamba amasomero, amalwaliro, n'abavunaanyizibwa ku byenjigiriza okuzuula amawulire (information) n'obuweereza (services) bye mwetaaga. Tusuubira nga kino kijja kuyamba be kikwatako okukola ku byetaago byabwe mu ngeri esinga okuba ennungi eyo jebujja.

Okusasulwa: Mutabani wo anaasasulwa olw'okwetaba mu kunoonyereza kuno?
Ojakuwebwayo omutwalo gwa silingi gumu (10,000/=) olw'obudde bwo. Mutabani wo tajja kusasulwa olw'okwetaba mu kunoonyereza kuno, mpozzi ajja kuweebwayo ka peni n'akatabo ko n'akokunywa akagonvu olw'obudde bwe ne kawefube gw'anaaba ataddemu.

Emmizi (**Confidentiality**): **Ebintu bino binaamanyibwako abantu abalala?**
Tewali gwe tujja kubuulirako nti mutabani yeetabye mu kunoonyereza kuno. Tewali muntu yenna atakola mu kunoonyereza kuno gwe tujja kubuulirako ku bimukwatako era tujja kuba tukozesa namba (study number) mu kifo ky'e linnyalye. Wabula ebimukwatako biyinza okulabibwako ba Auditor.

Okutegeezebwa ebinaazuulibwa mu kunoonyereza: Onotegeezebwa ebinaazuulibwa mu kunoonyereza kuno?
Okunoonyereza kuno nga kuwedde tujakutegeza mutabani wo ne banne bajja kutegeezebwa ebinaaba bizuuliddwa era teri wekijja kulabikira oba kulaga nti mutabani wo yatuwa amawulire ago okugyako abo abakola ku kunonyereza kuno okumanya endowooza zeyatuwa.

Oluvanyuma tujja kubitegeeza n'abantu abalala omuli ba nasayansi, abakola ku by'obulamu, n'abantu abalala nga tubategeza nebyo byetunaba tuyize nga tukola okunonyereza. Kino tujja kikola nga tuyita mu kuwandiika zi lipooti, n'okusisinkana bonna be kikwatako.

Ebinaava mu kunoonyereza kuno era bya kuteekebwa mu butabo (journals) bwa sayansi obw'ensi yonna ko n'emikutu ja intaneti abantu abalala basobole okutuyigirako. Ebivudde mukunonyereza kuno era biyinza okutekebwa ku mukutu gwa London School of Hygiene and Tropical medicine abantu abalala gyebayinza okubisanga. Kino kitegeza nti tuyinza okudamu okwekenenya ebinaba bivudde mukunonyereza naye nga tewali ngeri yonna mu kwogera ebinaava mu kunoonyereza kuno mutabani wo bye yatubuulira ng'omuntu we bijja kulabikira.

UVRI-RESEARCH ETHICS COMMITTEE
VALID UNTIL
26 MAY 2023
APPROVED
P. O. BOX 49, ENTEBBE (U)

Okwebuuza: Ani gw'oyinza okw'ogerako naye oba okubuuza ebikwata ku kunoonyereza kuno?

Oli waddembe okubuuza ekibuuzo kyonna kati oba je bujja ng'oyita ku simu oba ku e-mail oba okujja ku MRC/UVRI kwe nnyini n'otulaba mu buntu.

Osobola okutuukirira:

Dr. Catherine Kansiime
MENISCUS trial Project Lead
Email: catherine.kansiime@mrcuganda.org
Essimu: +256 702438487

Bwoba olina ekibuuzo oba okwemulugunya ku ddembe ly'omwana wo ku by'okwetabakwo mu kunoonyereza kuno tukirira akakiiko ka UVRI akalondoola n'okulabirira okunonyereza ku simu +256 0414 321962 oba +256 716 321962

UVRI-RESEARCH ETHICS COMMITTEE
VALID UNTIL
★ 26 MAY 2023 ★
APPROVED
P. O. BOX 49, ENTEBBE (U)

EKITUNDU 2: OKUKKIRIZA (VERSION 1.2 AUGUST 2021)

Nga ntekako omukuno wamanga, nzikiriza mutabani wange okwetaba mukunonyereza kuno omuli:

- Okwetaba mukukubanya ebirowoozo okwawamu oba okwa sekinoomu
- Ebinava mukunonyereza kuno okukozesebwa n'okutegezako abanonyereza abalala naye nga ebikwata ku mutabani wange tebija kumanyibwa.

Ebibuuzo byange ebikwata ku kunoonyereza kuno byanukuddwa

Erinnya____

**Soma era Oddemu ebibuzo bino**	**Saza kwebyo byonaba osazewo**	
Osomye oba osomedwa ebikwata ku kunonyereza kuno?	Yee	Nedda
Waliwo omuntu omulala yenna akunyonyode ku kunonyereza kuno?	Yee	Nedda
Otegedde bulungi okunonyereza kuno kyekukwattako?	Yee	Nedda
Ebibuuzo byo ku kunonyereza kuno bididwamu bulungi?	Yee	Nedda
Otegedde bulungi nti oli wadembe okuva mukunonyereza kuno ?	Yee	Nedda
Oli musanyufu okukiriza mutabani wo okwetaba mukunonyereza kuno? [CONSENT]	Yee	Nedda

Student study number (IDNO): |__|__|__|__|__|__|__| School ID number |__|__|__|

Wandiika (Print) Amannya g'omuzadde/avunaanyizibwa ku mwana/Eyaweebwa Obuvunaanyizibwa ________________________________

Omukono gwo'omuzadde(signature)______________________

Date of consent (IDATE): |__|__|/|__|__|/|__|__|__|__|
dd / mm / yyyy

Omwanawo naye ajja kuyisibwa mu kiwandiiko ky'okukkiriza bwanaaba naye akkirizza.

Omuzadde atasobola kusoma na kuwandiika: Omuzade nga tasobola kusoma na kuwandiika omujulizi yateekako omukono, omujulizi eno bwe kiba kisobose omuzadde ya mwerondera era tasaanye kuba nga alina akakwate konna n'abakola ku kunoonyereza kuno.

Wandiika (Print) Erinnya ly'omujulizi ______________________ Ekyenkumu ky'omuzadde

Omukono (Signature) g'omujulizi_______________________

Enakku z'omwezi _______________ Day/month/year

To be completed by the researcher
I confirm that the individual has given consent freely.
Name of researcher: ____________________ Date:|__|__|/|__|__|/|__|__|__|__|
dd / mm / yyyy
Signature: __________________

UVRI-RESEARCH ETHICS COMMITTEE VALID UNTIL 26 MAY 2023 APPROVED P. O. BOX 49, ENTEBBE (U)
