# Supplementary material for: Menstrual health interventions, schooling, and mental health problems among Ugandan students (MENISCUS): study protocol for a school-based cluster-randomised trial
Source: Trials. 2022 Sep 7;23:759. doi: 10.1186/s13063-022-06672-4 (PMC9449307; doi:10.1186/s13063-022-06672-4)
Supplement: Supplementary file 2 — Additional file 2. [file 13063_2022_6672_MOESM2_ESM.zip › ANACA8~1R1.PDF]

MRC/UVRI and LSHTM Uganda Research Unit

Medical Research Council MRC | Uganda Virus Research Institute | LONDON SCHOOL of HYGIENE &TROPICAL MEDICINE

Information and consent form for parents/guardians of male students (or the respective school head teacher or their authorized designate in the absence of a Parent) for individual interviews in the MENISCUS trial.

**Project title:**	Menstrual health interventions, schooling and mental health symptoms among Ugandan students (MENISCUS): a school-based cluster-randomised trial
**Funder:**	UK Joint Global Health Trials (Medical Research Council-Department for International Development-Wellcome Trust) Grant # MR/V005634/1
**Research Site:**	Wakiso and Kalungu Districts<br>C/o MRC/UVRI and LSHTM Uganda Research Unit.<br>Plot 51-59, Nakiwogo Road<br>P O Box 49, Entebbe, Uganda<br>Tel: +256(0) 417 704000; (0)312 262910/1; (0)702 438487
**Principal Investigators:**<br><br><br><br><br>**Local Principal Investigator:**	**1. Prof Helen Weiss,**<br>Professor of Epidemiology and Director of the MRC Tropical Epidemiology Group, London School of Hygiene and Tropical Medicine (LSHTM), UK<br>*Email: helen.weiss@lshtm.ac.uk*<br>**2. Prof Janet Seeley**<br>Professor of Anthropology and Health, London School of Hygiene and Tropical Medicine (LSHTM), UK<br>and Head of Social Science Programme, MRC/UVRI and LSHTM Uganda Research Unit.<br>*Email: janet.seeley@lshtm.ac.uk*
**Trial Manager:**	Dr. Catherine Kansiime,<br>MRC/UVRI and LSHTM Uganda Research Unit<br>*Email:Catherine.Kansiime@mrcuganda.org*

Summary (What you should know about this study):

- The aim of the study is to assess whether a school-based intervention focused on improving management of menstrual periods improves education, health and well-being outcomes among girls in secondary school in Wakiso and Kalungu districts in Uganda.
- This document explains the purpose of this study and asks whether you agree for your son to participate in the study. We will explain what he will be asked to do if you and he agree to participate.
- Your son's participation is completely voluntary. You or your son have the right for your son to take part in the study or to agree to take part now and change your mind later.
- Whatever you decide will not affect your son's regular healthcare and support.
- Please review this form carefully. Ask any questions before you make a decision.

You will be given a copy of this form to keep.

UVRI-RESEARCH ETHICS COMMITTEE
VALID UNTIL
★ 28 MAY 2023 ★
APPROVED
P. O. BOX 49, ENTEBBE (U)

Part I: Information about this study
Introduction
The MENISCUS trial is led by scientists at the London School of Hygiene & Tropical Medicine, MRC/UVRI and LSHTM Uganda Research Unit, with our partner WoMena Uganda.

We are carrying out research to guide our secondary schools to identify practical ways of helping girls to become and stay healthier and complete studying at school through improved management of menstrual periods. We are also interested in the role that boys' play in improving menstrual health in school. We have received permission to conduct this research from your school administration, the district, the Ministry of Education and Sports, and the Research Ethics Committees of the UVRI, LSHTM and Uganda National Council of Science and Technology (UNCST).

We invite your son to be part of this research. It is optional for you to choose whether or not you want your son participate in this research. If you agree to allow your son to participate, we shall ask your son for approval as well. Both of you have to approve before your son can be involved.

Please feel free to ask us questions now or later using our contact information which is indicated below. We will take time to explain to you.

Purpose
The purpose of the MENISCUS study is to see whether a health promotion intervention in secondary schools improves menstrual health (i.e. how girls manage their periods safely and confidently). We want to learn whether the package is likely to improve education, health and well-being outcomes among girls, and menstrual health knowledge and attitudes towards periods among boys. We are also interested in the role that boys' play in improving menstrual health in school. If the intervention is successful, it could be introduced in other schools in Uganda.

Selection
We request your son to participate in this research because he is a male secondary school student starting Form 2 in one of 60 schools chosen for this study. We are seeking your consent because you are the parent/guardian of your son.

Voluntary Participation
It is optional for your son to participate in this research. You or he can choose to say no. That decision shall not affect any services that you and your family receive at the secondary school and/or health facilities. You can ask as many questions as you like and we shall be available to answer them. You don't have to decide today. You can think about it and tell us what you decide later. You can agree that the school head teacher or authorized designate shall provide exceptional signed consent on your behalf if you are not available or accessible for documented reasons.

Procedures
There are 60 schools in the trial. Of these, half (30) will be randomly selected to receive the MENISCUS intervention. In these 30 schools, pupils in Form 2 at the start of 2022 will received education about puberty and menstruation and improvements to school toilet facilities. The intervention will be available throughout 2022. The schools which do not receive the MENISCUS intervention in 2022 will have the opportunity to receive it in 2023.

We request your son to participate in a group discussion with a small group of other boys on school premises and/or an individual interview. They will be guided by our trained adolescent-friendly researchers who will be formally introduced to the school authorities and will wear personal identity cards. The researcher will ask about your son's perceptions of the intervention. The discussions will

UVRI-RESEARCH ETHICS COMMITTEE
VALID UNTIL
2 8 MAY 2023
APPROVED
P. O. BOX 49, ENTEBBE (U)

take place at an agreed venue or within the school premises. The entire discussion will be tape-recorded and the tapes will be kept securely in lockable cabinets/cupboards at our research office. The information recorded is confidential, and no one else except the researchers or other ethical eligible person(s) such as the ethics review committees with regulated access to the tapes will be allowed to listen to the tapes. Nobody's name will be mentioned on the tapes and your son will not be able to be identified.

Risks and discomfort: Is the study bad or dangerous for your son?
Your son may feel uncomfortable talking about some of the topics. He will be encouraged to freely ask as many questions as possible and to discuss beliefs and myths about menstruation.

Benefits: Is there anything good that happens to your son from participating?
Your son's participation is likely to help us, the schools, the parents or guardians, health facilities and the education and health authorities to find out more about school health information and service needs. We hope that these will help all the relevant people to meet those needs better in the future.

Reimbursements: Will your son receive anything for being in the study?
You will be given 10,000 shillings to compensate for your time and effort. He will not be paid to take part in this research. However, he will be given a pen, a hardcover note book and a soft drink to compensate for her time and effort.

Confidentiality: Is anybody going to know about this?
We will not tell other people that your son was involved in this research. We shall not share personal information that identifies him to anyone who does not work in this research. Any information about him will have a study number on it instead of his name. However, your son's data may be seen by auditors.

Sharing the findings: Will you be told the study results?
When this research is completed, we shall inform your son, her peers and parents/guardians about the results obtained from the trial. The results will never be reported in a way that allows anyone except members of the research team to know what you specifically told us or any of the individual results we obtained from you. We will also share the research results with authorities at the school, district and national levels, including what we have learnt.

Afterwards, we will be telling other people, scientists, health workers and others, what we found. We will do this by writing and sharing reports and by going to meetings with people who are interested in this work. The research findings will be published in international science journals and electronic websites so that other people may learn from us. Data may be made available in the public domain via the London School of Hygiene and Tropical Medicine data repository. This means that it may be used for further analyses. The data will be anonymised i.e. it cannot be linked to your son.

Who to Contact: Who can you talk to or ask questions about this study?
You can ask us questions now or later by telephone, e-mail, post or at the physical addresses indicated on the assent/consent form to be given to you. If you are nearby, you can come and see us.

You can contact any of the following about this research

a) Dr. Catherine Kansiime, MENISCUS Trial Project Lead
Email: Catherine.Kansiime@mrcuganda.org; Phone number +256 702438487

If you have any questions, complaints or concerns about your rights as a person involved in this research, please contact: UVRI Research Ethics Committee: Phone number +256 0414 321962 or +256 716 321962

UVRI-RESEARCH ETHICS COMMITTEE
VALID UNTIL
26 MAY 2023
APPROVED
P. O. BOX 49, ENTEBBE (U)

PART 2: PARENTAL CONSENT (VERSION 1.2, AUGUST 2021)

By signing below I consent for my son to participate in the study as described above, including for:

- My son to participate in a group discussion or individual interview
- All anonymised data collected to be used as part of the research and shared with other researchers

My questions concerning this study have been answered by ……………………………..

**Please read each question below**	**Please circle all you agree with:**	
Have you read (or had read to you) information about this project?	Yes	No
Has somebody else explained this project to you?	Yes	No
Do you understand what this project is about?	Yes	No
Have you had any questions answered in a way you understand?	Yes	No
Do you understand that it is ok to stop taking part at any time?	Yes	No
Are you happy for your son to take part in this study? [CONSENT]	Yes	No

Student study number (IDNO): |__|__|__|__|__|__| School ID: |__|__|__|

(NAME): ____________________________________

Signature of Parent/Guardian/Authorized Delegate: ____________________

Date of interview (IDATE): |__|__|/|__|__|/|__|__|__|__|

dd / mm / yyyy

An Informed Assent Form will be completed by your son, if he also agrees.

If literacy challenged: A literate witness must sign (if possible, this person should be selected by the participant and should have no connection to the research team). Literacy challenged parents/guardians should include their thumb print as well.

Print name of witness_____________________ **AND** **Thumb print of participant**

Signature of witness ______________________

Date ________________________ **Day/month/year**

To be completed by the researcher: I confirm that the individual has given consent freely.

Name of researcher: ______________________ Date:|__|__|/|__|__|/|__|__|__|__|

dd / mm / yyyy

Signature: ___________________

UVRI-RESEARCH ETHICS COMMITTEE
VALID UNTIL
26 MAY 2023
APPROVED
P. O. BOX 49, ENTEBBE (U)
