# Supplementary material for: Menstrual health interventions, schooling, and mental health problems among Ugandan students (MENISCUS): study protocol for a school-based cluster-randomised trial
Source: Trials. 2022 Sep 7;23:759. doi: 10.1186/s13063-022-06672-4 (PMC9449307; doi:10.1186/s13063-022-06672-4)
Supplement: Supplementary file 2 — Additional file 2. [file 13063_2022_6672_MOESM2_ESM.zip › ANNEX2~1R1.PDF]

MRC/UVRI and LSHTM Uganda Research Unit

Medical Research Council · Uganda Virus Research Institute · London School of Hygiene & Tropical Medicine

Information and assent form for female students in the secondary schools invited to participate in the MENISCUS trial

**Project title:**	Menstrual health interventions, schooling and mental health symptoms among Ugandan students (MENISCUS): a school-based cluster-randomised trial
**Funder:**	UK Joint Global Health Trials (Medical Research Council-Department for International Development-Wellcome Trust) Grant # MR/V005634/1
**Research Site:**	Wakiso and Kalungu Districts<br>C/o MRC/UVRI and LSHTM Uganda Research Unit.<br>Plot 51-59, Nakiwogo Road<br>P O Box 49, Entebbe, Uganda<br>Tel: +256(0) 417 704000; (0)312 262910/1; (0)702 438487
**Principal Investigators:**	**1. Prof Helen Weiss,**<br>Professor of Epidemiology and Director of the MRC Tropical Epidemiology Group, London School of Hygiene and Tropical Medicine (LSHTM), UK<br>*Email: helen.weiss@lshtm.ac.uk*
**Local Principal Investigator:**	**2. Prof Janet Seeley**<br>Professor of Anthropology and Health, London School of Hygiene and Tropical Medicine (LSHTM), UK<br>and Head of Social Science Programme, MRC/UVRI and LSHTM Uganda Research Unit.<br>*Email: janet.seeley@lshtm.ac.uk*
**Trial Manager:**	Dr. Catherine Kansiime,<br>MRC/UVRI and LSHTM Uganda Research Unit<br>*Email:Catherine.Kansiime@mrcuganda.org*

Summary (What you should know about this study):

- The aim of the study is to assess whether a school-based intervention focused on improving management of menstrual periods improves education, health and well-being outcomes among girls in secondary school in Wakiso and Kalungu districts in Uganda.
- This document explains the purpose of this study, and what you will be asked to do if you agree to participate.
- Your participation is completely voluntary. You have the right to take part in the study or to agree to take part now and change your mind later.
- Whatever you decide will not affect your regular healthcare and support.
- Please review this form carefully. Ask any questions before you make a decision.

You will be given a copy of this form to keep.

UVRI-RESEARCH ETHICS COMMITTEE
VALID UNTIL
26 MAY 2023
APPROVED
P. O. BOX 49, ENTEBBE (U)

Part I: Information about this study

Introduction

The MENISCUS trial is led by scientists at the London School of Hygiene & Tropical Medicine, MRC/UVRI and LSHTM Uganda Research Unit, with our partner WoMena Uganda.

We are carrying out research to guide our secondary schools to identify practical ways of helping girls to become and stay healthier and complete studying at school through improved management of menstrual periods. We have received permission to conduct this research from your school administration, the district, the Ministry of Education and Sports, and the Research Ethics Committees of the UVRI, LSHTM and Uganda National Council of Science and Technology (UNCST).

We invite you to be part of this research. It is optional for you to choose whether or not you want to participate in this research. We shall also ask your parent/guardian for permission as well. Both of you have to agree before you can be involved.

Please feel free to ask us questions now or later using our contact information which is indicated below. We will take time to explain to you.

Purpose

The purpose of the MENISCUS study is to see whether a health promotion intervention in secondary schools improves menstrual health (i.e. how girls manage their periods safely and confidently). We want to learn whether the package is likely to improve education, health and well-being outcomes among girls, and menstrual health knowledge and attitudes towards periods among boys. If the intervention is successful, it could be introduced in other schools in Uganda.

Selection

We are asking all female secondary school students starting Form 2 in one of 60 schools chosen for this trial to participate.

Voluntary Participation

It is optional for you to participate in this research. You or your parent/guardian can choose to say no. That decision shall not affect any services that you and your family receive at the secondary school and/or health facilities. You can ask as many questions as you like and we shall be available to answer them. You don't have to decide today. You can think about it and tell us what you decide later.

Procedures

There are 60 schools in the trial. Of these, half (30) will be randomly selected to receive the MENISCUS intervention. In these 30 schools, all students in Form 2 at the start of 2022 will receive education about puberty and menstruation, improvements to school toilet facilities, and will have the option to participate in a drama skit relating to menstrual health. You will also sit two educational assessments covering recently taught criteria. The intervention will be available throughout 2022. The schools which do not receive the MENISCUS intervention in 2022 will have the opportunity to receive it in 2023.

If you agree to participate in the study, you will be asked to participate in the following activities: two self-completed questionnaires (40-60 minutes); the self-collection of vaginal swabs; and receiving a menstrual management kit. Some girls will be asked to participate in an individual interview with a researcher (about 60 minutes), a group discussion (1-2 hours), and/or completion of a diary on school attendance, menstruation and pain.

UVRI-RESEARCH ETHICS COMMITTEE
VALID UNTIL
26 MAY 2023
APPROVED
P. O. BOX 49, ENTEBBE (U)

1) Questionnaires at the start and end of the study (40-60 minutes) and use of assessment scores:
Girls in all 60 schools will be asked to complete a questionnaire at the beginning of the study (~2022) and at the end of the study (~2023). The data will be collected using tablet computers and our trained researchers will explain to you how to use these to complete the questionnaires. If you do not wish to answer some of the questions, you may skip them and move to the next question. In some cases, you may be asked to answer the questions using a paper questionnaire instead. These data will be entered by trained research assistants into a password-protected electronic database. The databases will not contain any information that can identify you.

We would also like to use your score from the educational assessments in our research. Your performance on these exams will not affect your school standing. Individual scores will be used for research purposes only and will not be shared with you, your teachers, or anyone else.

The trained researchers and research assistants will maintain safe custody of paper forms with identification data, including contact details in lockable cabinets and/or cupboards. This will be done to preserve privacy and confidentiality of participants. Nobody will be able to identify you from the research reports, presentations and publications.

2) Provision of Menstrual Management kit
Girls in the 30 intervention schools will be offered a kit which contains reusable sanitary pads provided in a bag with knickers, a water bottle, soap and a towel. You will be asked to participate in a session led by a teacher or a senior girl who has been trained by an expert trainer in menstrual health and how to use re-usable pads. They will show you how to use the re-usable pads, and will discuss any concerns you might have about this. You will be asked to try using the re-usable pads for the next 12 months, if you feel comfortable doing so. If you are experiencing any problems using the reusable pads, you are encouraged to discuss these with the expert trainer, teacher, peer, parents, guardian, school nurse, or the clinical officer on the project. The kit will also include vouchers for pain relief medication. Each voucher can be exchanged for up to 6 paracetamol or ibuprofen tablets per month from the school nurse or specified teacher. If you agree to participate in the study, there is no obligation to use the pads, the vouchers or the pain relief medication.

Girls in the 30 control schools will be offered this kit at the end of the study.

3) Complete a daily diary on school attendance, menstruation and pain
Some girls who have agreed to participate in the study will be asked to complete a daily diary on school attendance, menstruation and pain. This will be administered for a minimum of 12 weeks starting around the end of 2022. MENISCUS research assistants will guide you on how to complete the daily diaries if you are selected. Data from the daily diaries will be entered electronically at the end of each term when diaries are collected.

4) Group discussions and/or interviews among students (1-2 hours)
Some girls in the intervention schools will be asked to take part in a discussion with a small group of other girls and/or individual interviews, on school premises. They will be guided by our trained adolescent-friendly researchers who will be formally introduced to the school authorities and will wear personal identity cards. The researcher will ask about your perceptions of the intervention and how they affected how you manage your periods. The discussions will be tape-recorded and the tapes will be kept securely in lockable cabinets/cupboards at our research office. The information recorded is confidential, and no one else except the researchers or other ethical eligible person(s) such as the ethics review committees with regulated access to the tapes will be allowed to listen to the tapes. Nobody's name will be mentioned on the tapes and you will not be able to be identified.

UVRI-RESEARCH ETHICS COMMITTEE
VALID UNTIL
★ 26 MAY 2023 ★
APPROVED
P. O. BOX 49, ENTEBBE (U)

5) <u>Genital symptoms and self-collection of vaginal swabs</u>
In the questionnaire at the end of the study, you will be asked about how you clean your genitals and any current urogenital symptoms (like itching in the genital area or burning when urinating). If you report having these symptoms, you will be referred to an appropriate clinic for management and treatment as needed. If you have symptoms of a urinary tract infection (UTI) (e.g. urinary burning and frequency) we will ask you for a urine sample to be tested for UTI.

You will also be asked to provide self-two collected vaginal swabs at endline that will be tested for bacterial vaginosis (BV) and candida. Bacterial vaginosis is not a sexually transmitted infection and you do not need to be sexually active to have it. You will receive training on how to do this from the study staff. The swabs will be sent to our laboratory. One swab will be used to test for bacterial vaginosis. The other swab will be stored for future testing. We may need to ship the second swab overseas for detailed testing about the types of bacteria present. We will not give you the results of these tests but you will receive appropriate treatment for any symptomatic infection from the clinic.

Why might one sample be shipped overseas?
The second vaginal swab needs detailed testing so we can identify which types of bacteria are present. This testing needs specialist equipment which is not commonly used in Uganda. The samples will have a study number but not your name or other identifying information about you.

Risks and discomfort: Is this bad or dangerous for you?
We are asking you to share with us some very personal and confidential information including about your periods and how you manage them. You may feel uncomfortable talking about some of the topics. You will be taught and/or helped by an experienced team who have experience in helping young girls use re-usable pads. You will also be taught how to wash and dry the re-usable sanitary pad, identify symptoms of infection and whom to report to. If you do not follow these instructions, there is a risk of infection or irritation when you use them. However, in case of any adverse events or problems with the reusable pads, the girls should contact the school nurse for assistance or for referral if the nurse cannot handle the case. We have a clinical officer to whom adverse events which cannot be handled by the school nurse will be referred. You are encouraged to report early any challenges you experience with using the reusable pads.

There is a risk that you may feel embarrassed answering questions about genital hygiene and symptoms or being asked to self-collect vaginal swabs. To mitigate the risk of embarrassment, a self-completed form on a tablet will be used, and we will develop a short, animated video on basic anatomy and anticipated discomfort for you to view before taking the vaginal swab. Taking these swabs is painless and safe and will not harm you in any way, even if you have never had sexual intercourse. The swabs should not cause any discomfort as they are very thin. They will not break or tear any skin near the opening of your vagina.

You will be encouraged to freely ask as many questions as possible and to discuss beliefs and myths about the female anatomy and menstruation. This information will help you make decisions on how best to manage your menstruation. You will be given re-usable pads to manage your menstruation.

Benefits: Is there anything good that happens to you?
You may benefit from having the menstrual kit in terms of managing your menstruation more easily. The pain relief medication can also help with cramps that you might get during your periods. You may find the diary helpful for you to monitor your menstrual cycle and to know when to expect your next period. Answering the questions about urogenital symptoms and providing a urine sample will enable you to be treated for these symptoms. There is no direct benefit to you of providing the swab samples.

UVRI-RESEARCH ETHICS COMMITTEE
VALID UNTIL
28 MAY 2023
APPROVED
P. O. BOX 49, ENTEBBE (U)

Your participation is likely to help us, the schools, the parents or guardians, health facilities and the education and health authorities to find out more about your health information and service needs. We hope that these will help all the relevant people to meet those needs better in the future. Taking part in this research may also remind you to think deeply about your health and your future.

Reimbursements: Will you get anything for being in the research?
You will not be paid to take part in this research. However, you will be given a pen, a hardcover note book and a soft drink to compensate for your time and effort.

Confidentiality: Is anybody going to know about this?
We will not tell other people that you were involved in this research. We shall not share personal information that identifies you to anyone who does not work in this research. Any information about you will have a study number on it instead of your name. However your data may be seen by auditors.

Sharing the Findings: Will you be told the study results?
When this research is completed, we shall inform you, your peers and parents/guardians about the results obtained from the trial. The results will never be reported in a way that allows anyone except members of the research team to know what you specifically told us or any of the individual results we obtained from you. We will also shall share the research results with authorities at the school, district and national levels, including what we have learnt.

Afterwards, we will be telling other people, scientists, health workers and others, what we found. We will do this by writing and sharing reports and by going to meetings with people who are interested in this work. The research findings will be published in international science journals and electronic websites so that other people may learn from us. Data may be made available in the public domain via the London School of Hygiene and Tropical Medicine data repository. This means that it may be used for further analyses. All data will be anonymised i.e. it cannot be linked to you.

Who to Contact: Who can you talk to or ask questions about this study?
You can ask us questions now or later by telephone, e-mail, post or at the physical addresses indicated on the assent/consent form to be given to you. If you are nearby, you can come and see us.

You can contact any of the following about this research:

Dr. Catherine Kansiime, MENISCUS Trial Project Lead
Email: Catherine.Kansiime@mrcuganda.org; Phone number +256 702438487

If you have any questions, complaints or concerns about your rights as a person involved in this research, please contact: UVRI Research Ethics Committee: Phone number +256 0414 321962 or +256 716 321962

You may re-watch the video about the study at this link:
https://www.lshtm.ac.uk/research/centres-projects-groups/meniscus#resources

UVRI-RESEARCH ETHICS COMMITTEE
VALID UNTIL
26 MAY 2023
APPROVED
P. O. BOX 49, ENTEBBE (U)

PART 2: ASSENT (VERSION 1.2, AUGUST 2021)

By singing below I assent to participate in the study as described above, including:

- To self-complete the two study questionnaires
- To receive a menstrual management kit
- To self-collect two vaginal swabs, one of which will be stored and may be shipped overseas
- To participate in group discussions and complete a diary on school attendance and menstruation, if selected for these activities
- For all anonymised data collected to be used as part of the research and shared with other researchers

My questions concerning this study have been answered by ……………………………….

**Please read each question below**	**Please circle all you agree with:**	
Have you read (or had read to you) information about this project?	Yes	No
Has somebody else explained this project to you?	Yes	No
Do you understand what this project is about?	Yes	No
Have you had any questions answered in a way you understand?	Yes	No
Do you understand that it is ok to stop taking part at any time?	Yes	No
Are you happy to take part in this study? [ASSENT]	Yes	No

Student study number (IDNO): |__|__|__|__|__|__|__|__|

Name of student: ____________________________________

Signature of student: ____________________

Date of consent (IDATE): |__|__|/|__|__|/|__|__|__|__|
dd / mm / yyyy

To be completed by the researcher
I confirm that the individual has given assent freely.

Name of researcher: ______________________ Date:|__|__|/|__|__|/|__|__|__|__|
dd / mm / yyyy

Signature: ___________________

The Parent/Guardian has signed an informed consent (Yes=1, No=2) |__|
_____ (initialed by researcher/assistant)

UVRI-RESEARCH ETHICS COMMITTEE
VALID UNTIL
26 MAY 2023
APPROVED
P. O. BOX 49, ENTEBBE (U)
