# Supplementary material for: Menstrual health interventions, schooling, and mental health problems among Ugandan students (MENISCUS): study protocol for a school-based cluster-randomised trial
Source: Trials. 2022 Sep 7;23:759. doi: 10.1186/s13063-022-06672-4 (PMC9449307; doi:10.1186/s13063-022-06672-4)
Supplement: Supplementary file 2 — Additional file 2. [file 13063_2022_6672_MOESM2_ESM.zip › ANNEX2~2R1.PDF]

MRC/UVRI and LSHTM Uganda Research Unit

Olupapula oluliko Amawulire agasaba Abaana abawala mu masomero ga siniya Okukkiriza Okwetaba mu kunoonyereza Kwa MENISCUS.

**Project title:**	Menstrual health interventions, schooling and mental health symptoms among Ugandan students (MENISCUS): a school-based cluster-randomised trial
**Funder:**	UK Joint Global Health Trials (Medical Research Council-Department for International Development-Wellcome Trust) Grant # MR/V005634/1
**Research Site:**	Wakiso and Kalungu Districts<br>C/o MRC/UVRI Uganda Research Unit on AIDS<br>Plot 51-59, Nakiwogo Road<br>P O Box 49, Entebbe, Uganda<br>Tel: +256(0) 417 704000; (0)312 262910/1; (0)702438487
**Principal Investigators:**<br><br>**Local Principal Investigator:**	**1. Prof Helen Weiss,**<br>Professor of Epidemiology and Director of the MRC Tropical Epidemiology Group, London School of Hygiene and Tropical Medicine (LSHTM), UK<br>*Email: helen.weiss@lshtm.ac.uk*<br>**2. Prof Janet Seeley**<br>Professor of Anthropology and Health, London School of Hygiene and Tropical Medicine (LSHTM), UK<br>and Head of Social Science Programme, MRC/UVRI<br>*Email: janet.seeley@lshtm.ac.uk*
**Trial Manager:**	Dr. Catherine Kansiime,<br>MRC/UVRI and LSHTM Uganda Research Unit<br>*Email:Catherine.Kansiime@mrcuganda.org*

Mu bufunze (By'olina okumanya ku kunoonyereza kuno):

- Ekigendererwa ky'okunoonyereza kwa MENISCUS kwe kumanya oba nga kinayambako mukulongosa ebyekusoma, obubonero obulabirwako eby'obulamu ebikwata kubwongo, okutumbula engeri abaana abawala jebasobola okubeera obulunji nga bali mu nsonga z'ekikyala awamu n'embeela y'obulamu bwabwe mu mamasomero ga siniya mu district ze Wakiso ne Kalungu mu Uganda.
- Ekiwandiiko kino kinnyonnyola ekigendererwa ky'okunoonyereza kuno ne ky'onasabibwa okukola singa onooba okkirizza okwetabamu.
- Okw'etaba mu kunoonyereza kuno kwa kyeyagalire. Dembe lyo okukwetabamu, oba okukwetabamu oluvannyuma n'okuvaamu.
- Kyonna ky'onaaba asazeewo tekijja kukosa ngeri jofunamu bujjanjabi wadde obuyambi.
- Soma ekiwandiiko kino n'obwegendereza era obuuze ekibuuzo kyonna ky'oyagala nga tonasalawo.

Ojja kuweebwa kopi ku kiwaandiiko kino

UVRI-RESEARCH ETHICS COMMITTEE
VALID UNTIL
26 MAY 2023
APPROVED
P. O. BOX 49, ENTEBBE (U)

Ekitundu Ekisooka: Ebikwata ku kunoonyereza kuno.

Enyanjula (Introduction)
Okunoonyereza kwa MENISCUS kukulembeddwamu banascience ku London School of Hygiene and Tropical Medicine, MRC/UVRI ne Tendekero lya (LSHTM) nga bakolerera wamu n'ekitongole kya WoMena Uganda.

Tukola Okunoonyereza kuno okulungamya amasomero ga Siniya okuzuula engeri ezisoboka ez'okuyamabamu abaana abawala okubeera abalamu n'okubeera ku somero obulungi nga bali mu nsonga z'ekikyala. Twafunye olukusa okukola okunonyereza kuno okuva kubakulu b'essomero lyo, ekitongole ky'ebyenjigiriza n'emizannyo n'obukiiko obulondoola okunonyereza obwa UVRI, LSHTM awamu ne Uganda National Council of Science and Technology (UNCST).

Tukusaba okkirize okwetaba mu kunoonyereza kuno. Ddembe lyo okukkiriza oba obutakkiriza. Tujakusaba nemuzadde wo olukusa olukukiriza oketabamu. Tuyina okufuna olukusa okuva eri muzaddewo nawe.

Oli waddembe okutubuuza ekibuuzo kyonna ky'oyagala kati oba oluvannyuma ng'oyita ku email ne namba z'esimu eziragiddwa wa manga era tujja kutwala obuvunaanyizibwa tukunnyonnyole otegeere.

Ekigendererwa (Purpose)
Ekigendererwa ky'okunoonyereza kwa MENISCUS kwe kulaba oba nga enkola yokutumbula eby'obulamu mu mumasomero ga siniya enayambako mu kulongosa ensonga z'ekikyala (engeri abaana abawala jebasobola okubeera obulunji nga bali mu nsonga z'ekikyala) n'okumanya oba nga kinaayambako mu kulongosa eby'okusoma, eby'obulamu mubana abawala awamu n'okumanya kwa baana abalenzi kubikwata kusonga za bakyala. Okunonyereza kunno bwekunaba kuvudemu ebirungi, kujja kutongozebwa mumasomera amalala mu Uganda.

Okulonda (Selection)
Tusaba abana bona abawala aba siniya 2 mu masomero 60 agaalondeddwa okukoleramu okunonyeleza kuno.

Okwetabamu kwakyeyagalile
Okwetaba mu kunoonyereza kuno kwa kyeyagalire. Ggwe oba muzaddewo muli baddembe okugaana. Okusalawo obuteegatta mu kunoonyereza kuno tekijja kukosa gwe ne famileyo bye mulina kufuna ku somero wadde ewajjanjabirwa wonna. Oli wa ddembe okutubuuza ebibuuzo byonna era tuli beetegefu okubyanukula.Osobola obutasalawo kati, oli waddembe okusooka okukirowoozaako n'otubuulira oluvannyuma ky'onooba osazeewo.

Procedures
Okunonyereza kuno kwetabiddwamu amasomero 60 nga amasomero 30 gajja kulondebwa okufuna ettu lya MENISCUS. Mu masomero gano 30, abayizi mu siniya ey'okubiri kuntadikwa y'omwaka 2022 bajakusomesebwa ku nkyukakyuka ezibawo nga omwana avubuka, ensonga z'ekikyala awamu n'okulongosa kabuyonjo z'amassomero era bajjakubera n'omukisa okufuna ettu lyebikozesebwa omuli paadi ezikozesebwa nezozebwa, okwetaba mu katemba oba emizanyo nekigendererwa kyokugyawo okusekelerwa nga abana abawala bali munsonga z'ekikyala n'okusobola okufuna eddagala eriyambako okukendeza obulumi nga oli munsonga. Ettu lijakugabibwa mumasomero mumwaka gwa 2022 gwona ate ago amasomero aganaba tegafunye etu lino, gajja kuba n'omukisa okufuna etu lino mu 2023.

UVRI-RESEARCH ETHICS COMMITTEE
VALID UNTIL
26 MAY 2023
APPROVED
P. O. BOX 49, ENTEBBE (U)

Bwonaba okiriza okwetaba mukunonyereza kuno, ojja kusabibwa okwetaba mubintu ebyenjawulo omuli okudamu ebibuzo (Self completed questionnaire 40-60 minutes) ojakusabibwa okutuwayo ebikeberebwa (self-collection of vaginal swabs) n'okufuna ettu lyebikozesebwa abakyala nga bali munsonga. Abawala abamu bajakusabibwa okudamu ebibuzo ebyasekinomu nga bali n'omunonyereza waffe (Individual interviews about 60 minutes) oba oba okukubaganya ebirowoozo okwawamu (Group discussion 1-2 hours) oba okujuzamu Dayale okulamba ennaku z'onaabeera nga ku Ssomero ne z'onaabeera nga mu nsonga z'ekikyala awamu na buli ddi wofuna obulumi nga oli munsonga(completion of a diary on school attendance, menstruation and pain).

1) <u>Okuddamu ebibuuzo by'okwesomero n'okweyanukulira kuntandikwa ne kunkomerero y'okunonyereza(40-60 minutes) wamu n'okukozesa ebinaba bivudde mukigezo.</u>

Abawala okuva mu masomero 60 mujakusabibwa okwanukula ebibuuzo kuntandikwa y'okunonyereza kuno mu (~2022) ne kunkomerero (~ 2023).Tujja kozesa bu kompyuta obutono (tablet computers) Abakola ku kunoonyereza kuno bajja kunyonyola engeri eyokukozesamu bucomputer buno. Ekibuuzo kyonna ky'onaawulira nga toyagala ku kiddamu ojja kuba wa ddembe okukireka n'ogenda ku kirala. Oyinza okusabibwa okudamu ebibuzo kumpapula (paper questionnaire) abakola ku kunoonyereza baakukuuma ebiwandiiko byonna ebikwata ku beetabye mu kunoonyereza kuno nga bya kyama era bya kusibirwa mu kabada ko n'okuyingizibwa mu Kompyuta esibibwe n'ekigambo eky'ekyama (password-protected electronic database). Era tewali linnya lya muntu yenna liggya kw'ogerwako.

Mukunonyereza kwaffe, tuja kwagala okukozesa obubonero bwonaba ofunye mu kigezo era. ebinava mu bigezo bino tebija kukosa byakusoma kwo. Ebinaba bivudde mu bigezo bijakukozesebwa mukunonyereza kwaffe kwokka era teri muntu mulala yenna ajakubimanya kabe gwe oba abasomesa.

Abakola ku kunoonyereza kuno baakukuuma ebiwandiiko byonna ebikwata ku beetabye mu kunoonyereza kuno nga bya kyama era bya kusibirwa mu kabada. Naye nga tewali ngeri yonna mu kwogera ebinaava mu kunoonyereza kuno bye watubuulira ng'omuntu we bijja kulabikira.

2) **<u>Okugaba ettu ely'ebikozesebwa mu nsonga z'ekikyala.</u>**
Abawala bonna mu masomero 30 aganaba galondedwa bajja kuweebwa ettu ly'ebikozesebwa mu Nsonga z'ekikyala nga mulimu paadi ezoozebwa ne ziddamu ne zikozesebwa, empale ezomunda, akaccupa omunatekebwa amazzi, sabuuni n'ekatowel. Ojja kusabibwa okwetaba mu musomo ogunaakubirizibwa omusomesa oba omu ku bayizi banno omuwala anaaba atendekeddwa abakugu mu nsonga z'ekikyala, ne paadi ezoozebwa ne ziddamu ne zikozesebwa. Bajja ku kulaga engeri y'okukozesaamu paadi ezo era bajja ku kunnyonnyola buli kyonoba oyagala okumanya ku nsonga eno. Ojja kusabibwa okukozesa paadi ezoozebwa ne ziddamu ne zikozesebwa okumala emyezi 12 ejinaddako bwonaaba takirinaako buzibu. Bwonaba olina obuzibu bwofunye mu kukozesa, paadi ezoozebwa okubirizibwa okutegeeza omukugu (expert trainer) omusomesawo (teacher) mwana munno (peer), omuzadde (parent) omukuza (guardian) omusawo wesomero (school nurse) oba dokita akwasaganya ensonga mukunonyereza kuno (Project Clinical officer) Muttu mujjakubamu ne bu kaadi bwonakozesa okufuna eddagala eliyambako okukakanya obulumi nga oli munsonga. Buli Kaadi ejakusobozesa okufuna empeke za Panadol oba Ibuprofen mukaaga (6) buli mwezi okuva eri omusawo wessomero oba omusomesa anaba alondedwa okugaba eddagala. Wokiriza okwetaba mukunonyereza kuno, sikyattekwa kukozesa paadi zino oba bukaadi bweddagala. Abawala mumasomero amalala amakumi assatu bajjakuwebwa ettu lino nga okunonyereza kuno kuwedde.

UVRI-RESEARCH ETHICS COMMITTEE
VALID UNTIL
26 MAY 2023
APPROVED
P. O. BOX 49, ENTEBBE (U)

3) Okujuzamu Dayale okulamba ennaku z'onaabeera nga ku Ssomero ne zonaabeera nga mu nsonga z'ekikyala awamu n'obulumi bwoyinza okufuna nga oli munsonga z'ekikyala.
Abawala abamu abanaba bakiriza okwetaba mu kunonyereza kuno bajakusabibwa okujuzamu dayale eyabuli lunaku nga ekwatagana ku kusoma ku somero, okugenda munsonga na buli ddi omwana omuwala wafuna obulumi nga ali munsonga era kino kijakukolebwa okumala sabiiti 12 okuva nga kunkomereo y'omwaka 2022. Abakola okunonyereza kuno bajja kunyonyola engeri y'okukozesa awamu n'okujuzamu dayale eno kasita onobera nga olondedwa. Tujja kujako dayale eyo ku buli nkomerero ya Taamu ela ebinaabeera mu Dayale bya kuyingizibwa mu Kompyuta.

4) Okukubaganya ebirowoozo okwa wamu / okwanukula ebibuuzo (1-2 hours)
Abawala abamu okuva mumasomero amakumi assatu (30) aganaba gafunye ettu lya MENISCUS bajakusabibwa okwetaba mukukubaganya ebirowozo ne bayizi bannamwe abawala abalala oba okwanukula ebibuuzo nga muli kusomero. Bino bigenda kukubirizibwa abavubuka abakola ku kunoonyereza kuno era nga abakulira e ssomero bajja kuba bamaze okubategeera mu butongole, nga bambadde ne kaada eziboogerako. Tujja kubaganya ebirowoozo ku bikwata ku ttu lya MENISCUS awamu n'okumanya engeri okunonyereza kuno gyekukuyambyemu nga oli mungonga z'ekikyala.Okukubaganya ebirowoozo kwa kubeera ku Somero era kujja kwatibwa ku butambi era obutambi obwo bwa kusibirwa mu kabada ku UVRI. Ebinaakwatibwa ku butambi bya kuumibwa nga bya kyama era tewali ajja ku biwulirako okujjako abakola ku kunoonyereza n'abalala abakkirizibwa mu mateeka agafuga okunoonyereza nga obukiiko obulondoola n'okulabirira okunonyereza. Era tewali linnya lya muntu yenna liggya kw'ogerwako mu butambi.

5) Obubonero bw'obukyafu mubukyala n'okwegyako ebikebelebwa(Genital symptoms and self-collection of vaginal swabs).
Kunkomerero y'okunonyereza ojja kusabibwa okuddamu ebibuuzo ebikubuza ku ngeri gy'oyonjamu ebitundu byo eby'ekyaama n'okumanya oba oyinamu obulwadde bwonna nga (okusiyibwa n'okulumiziba nga ofuuka). Bw'onaba otubulidde nti oyinamu kubulwadde buno, ojakusindikibwa ku dwaliro ofune obujanjabi. Bw'onatubulira nti ofuna okusiyibwa n'okulumizibwa nga ofuuka, tujakusaba otuwe omusulo gwo gukeberebwemu obulwadde bwa UTI.

Ojakusabibwa okutuwa Vaginal swabu biri ezinakozebwa okukebera Bacterial viginosis ne Candida kunkomerero y'okunonyereza Obulwadde bwa Bacterial viginosis buno tebusasanyizibwa lwa kwegatta na basajja era omuntu okuba nabwo tekitegeza nti yegatta n'abasajja. Abakola ku kunoonyereza kuno bajja kutendekebwa ku ngeri gyolina okukozesa ebikebera endwadde eno. Swabu zijakusindikibwa mu laboratory zikeberebwe. Swab emu ejakukozesebwa okukebera obuwuka mu bukyala ate endala ejakuterekebwa ekozesebwe mu biseera by'omumaso . Tuyinza okugisindika ebweru okwongera okwekenenya obuwuka obw'enjawulo obubera mu bukyala. Tetuja kubulira binaba bivudde mu kw'ekenenya kuno wabula ojakuwebwa obujanjabi singa obako obunonero byona bwonaba olaga.

Lwaki sampo emu ejakusindikibwa ebweru we gwanga?

Sampo eyokubiri ejakwetagibwa okwongera okwekenenya kitusobozese okumanya obuwuka obwenjawulo obusangibwa mu bukyala. Kino kyetagisa ebyuuma ebyamanyi byetutalina wano mu Uganda. Sampo zino tezijakuwandikibwako manya gamuntu wabula tujakukozesa enamba enekusiifu (study number) mu kifo ky'e linnyalyo era tewali kintu kyona ekiyinza okutegeza nti yegwe.

UVRI-RESEARCH ETHICS COMMITTEE
VALID UNTIL
26 MAY 2023
APPROVED
P. O. BOX 49, ENTEBBE (U)

Obutyabaga n'okuteteganyizibwa

Tujja kukubuuza ebikukwatako ng'omuntu n'ebikwata ku bulamubwo obw'ekyama n'engeli gyeweyisamu nga olimunsonga z'ekikyala ekiyinza okukuleetera obutawulira bulungi nga oby'ogerako. Ojja kusomesebwa oba okuyambibwa kunkozesa ya paadi ezozebwa nga oyambibwako tiimu eyabakungu mu kuyamba abawala abato kunkozesa ate ela n'okuyonja padi zino. Ojja kusomesebwa engeri y'okwozaamu paadi zino,engeri gyoyinza okumanya obubononero bw'endwadde n'ani gwoyinza okulaba singa oba ofunye obubonero obwo. Singa oba togoberedde biragiro bino wayiza okubalukawo akatyabaga kokukwatibwa obulwadde oba obutawulira bulungi. Wabula wewaberawo akatyabaga konna akaamanyi mukukozesa paadi ezozebwa ojakubera waddembe okutukirila omusawo w'esomero bwanaba tasobola kukuyamba munsonga eyo ojja kutukirila omusawo waffe. Osabibwa okwogera amangu eri omusawo singa oba ofunye obuzibu bwonna mu kukozesa paadi ezozebwa.

Oyinza obutawulira bulungi nga ayanukula ebimu ku bibuzo ebikwatagana n'engeri gyeyeyonjamu oba okusabibwa okukozesa swabu mu bukyala bwo. Okusobola okuziyiza kino, ojakuddamu ebibuzo ebinababiteredwa ku ka computer nga obyesomera era n'obyediramu era tujakutekako ne catoon oba ebifananyi ebinakusobozesa okumanya ekikula kyo, n'engeri gyoyinza okufunamu sampo. Okwejako sampo tekiyina buzibu bwonna bwekikuletera era ne swabu ezikozesebwa ntono nyo mu sayizi, tezisobola kuleta buzibu bwonna mubukyala bwo.

Mu kusomesebwa okunabawo ojja kuba waddembe okubuza ebibuzo byona awamu n'okukubaganya ebirowooza kubintu abantu bye balowooza kubutonde n'ensonga z'abakyala. Obubakka bunno bujja kukuyamba mungeri gyeweyisamu nga olimusonga z'ekikyala. Kino kijja kuyamba okusobola okukola okusalawo kungeli esinga gy'onakozesa nga oli munsonga zekikyala. Ojakuwebwa paadi ez'ozebwa nezidamu nezikozesebwa.

Okuganyurwa (benefits)

Ojakuganyurwa mukufuna ettu ly'ebikozesebwa nga oli mu nsonga z'ekikyala (menstrual kit) ,okufuna empeke ezitta obulumi obuleetebwa ensonga z'ekikyala era ne dayale egenda okukuweebwa nayo ejja kusobola okukuyamba okumanya lwonaddamu okugenda mu nsonga osobole okubera mwetegefu. Okuddamu ebibuuzo ku ndwadde z'omubukyala n'okuwayo sampo z'omusulo kijakukuyamba okufuna obujanjabi singa onaba osangidwamu nobubonero bwendwadde. Tojja kusasulwa olw'okuwayo swabu wabula okwetabaakwo mu kunoonyereza kuno kusobola okutuyamba, okuyamba amasomero, amalwaliro, n'abavunaanyizibwa ku byenjigiriza okuzuula amawulire (information) n'obuweereza (services) bye mwetaaga. Tusuubira nga kino kijja kuyamba be kikwatako okukola ku byetaago byamwe mu ngeri esinga okuba ennungi eyo jebujja. Ate era okwetabakwo mu kunoonyereza kuno kujja kukuyamba okulowooza ennyo ku bulamubwo n'ebiseera byo eby'omumaaso

Okusasulwa

Tojja kusasulwa olw'okwetaba mu kunoonyereza kuno, mpozzi ojja kuweebwayo ka peni n'akatabo akeddiba eggumu, n'akokunywa akagonvu olw'obudde bwo ne kaweefube gwonoba otaddemu.

Emmizi (Confidentiality)

Tewali gwe tujja kubuulirako nti weetabye mu kunoonyereza kuno. Tewali muntu yenna atakola mu kunoonyereza kuno gwe tujja kubuulirako ku bikwatako era tujja kuba tukozesa namba (study number) mu kifo ky'e linnyalyo. Wabula amawulire gotuwadde gayinza okulabibwako ba auditors.

UVRI-RESEARCH ETHICS COMMITTEE
VA[illegible]
★ 26 MAY 2023 ★
APPROVED
P. O. BOX 49, ENTEBBE (U)

Okutegeezebwa ebinaazuulibwa mu kunoonyereza
Okunoonyereza kuno nga kuwedde gwe ne bayizi banno muja kutegeezebwa ebinaaba bizuuliddwa era tujja kubitegeeza n'abazaddde, abakulira essomero lino ko aba Munisipaali n'egwanga lyonna okutwalira awamu. Tujja kubitegeeza n'abantu abalala omuli ba nasayansi, abakola ku by'obulamu, n'abantu abalala. Kino tujja kikola nga tuyita mu kuwandiika zi lipooti, n'okusisinkana bonna be kikwatako. Ebinaava mu kunoonyereza kuno era bya kuteekebwa mu butabo (journals) bwa sayansi obw'ensi yonna ko n'emikutu ja intaneti, abantu abalala basobole okutuyigirako. Ebivudde mukunonyereza kuno era biyinza okutekebwa ku mukutu gwa London School of Hygiene and Tropical medicine abantu abalala gyebayinza okubisanga. Kino kitegeza nti tuyinza okudamu okwekenenya ebinaba bivudde mukunonyereza naye nga tewali ngeri yonna mu kwogera ebinaava mu kunoonyereza kuno bye watubuulira ng'omuntu we bijja kulabikira.

Okwebuuza: Ani gw'oyinza okw'ogerako naye oba okubuuza ebikwata ku kunoonyereza kuno?

Oli waddembe okubuuza ekibuuzo kyonna kati oba je bujja ng'oyita ku simu oba ku e-mail oba okujja ku MRC/UVRI kwe nnyini n'otulaba mu buntu.

Osobola okutuukirira:

Dr. Catherine Kansiime
MENISCUS Trial Project Lead
Email: catherine.kansiime@mrcuganda.org
Essimu: +256 702438487

Bwoba olina ekibuuzo oba okwemulugunya ku ddembe lyo ku by'okwetabakwo mu kunoonyereza kuno tukirira akakiiko ka UVRI akalondoola n'okulabirira okunonyereza ku simu +256 0414 321962 oba oba +256 716 321962

UVRI-RESEARCH ETHICS COMMITTEE
VALID UNTIL
26 MAY 2023
APPROVED
P. O. BOX 49, ENTEBBE (U)

EKITUNDU 2: OKUKKIRIZA (VERSION 1.2,- AUGUST 2021)

Nga ntekako omukono wamanga, nzikiriza okwetaba mukunonyereza kuno omuli;

- Okwesomera n'okwanukula ebibuzo ebyemirundi ebbiri
- Okufuna ettu lye bikozesebwa munsonga
- Okwejako ebikeberebwa bya mirundi ebbiri nga ekimu ku byo kyakuterekebwa era kisobola n'okutwalibwa ebweru we gwanga.
- Okwetaba mu kukubaganya ebiroowozo okwawamu oba okwa sekinoomu n'okujuzamu dayale singa naba nondedwa okwetabamu
- Ebinava mukunonyereza kuno okukozesebwa n'okutegezako abanonyereza abalala naye nga ebikwatako tebija kumanyibwa.

Ebibuuzo byange ebikwata ku kunoonyereza kuno byanukuddwa: Erinnya_________________

Soma ebibuuzo bino wamanga ogolore ku Ye oba Nedda

Osomye/bakusomedde ebikwata ku pulojekiti eno?	Ye	Nedda
Waliwo omuntu akunyonnyodde pulojekiti eno?	Ye	Nedda
Otegedde pulojekiti eno kyeriko?	Ye	Nedda
Ebibuuzo byo byanukuddwa mu ngeri etegereekeka?	Ye	Nedda
Otegedde nti oli waddembe okubivaamu ekiseera kyonna?	Ye	Nedda
Okkiriza okwetaba mu kunoonyereza kuno?	Ye	Nedda

Enamba y'eyeetabye mu kunoonyereza (Student #) |__|__|__|__|__|__| School ID : |__|__|__|

(ERINNYA) ________________________________

Omukono (Signature) ________________________________

Ennaku z'omwezi: |__|__|/|__|__|/|__|__|__|__|
dd / mm / yyyy

Wano wakujjuzibwa akola ku kunoonyereza

To be completed by the researcher
I confirm that the individual has given assent freely.

Name of researcher: ______________________ Date:|__|__|/|__|__|/|__|__|__|__|
dd / mm / yyyy

Signature: ____________________

The Parent/Guardian has signed an informed consent (Yes=1, No=2) |__|
_____ (initialed by researcher/assistant)

UVRI-RESEARCH ETHICS COMMITTEE
VALID UNTIL
26 MAY 2023
APPROVED
P. O. BOX 49, ENTEBBE (U)

UVRI-RESEARCH ETHICS COMMITTEE
VA[illegible]
26 MAY 2023
APPROVED
P. O. BOX 49, ENTEBBE (U)
