# Supplementary material for: Menstrual health interventions, schooling, and mental health problems among Ugandan students (MENISCUS): study protocol for a school-based cluster-randomised trial
Source: Trials. 2022 Sep 7;23:759. doi: 10.1186/s13063-022-06672-4 (PMC9449307; doi:10.1186/s13063-022-06672-4)
Supplement: Supplementary file 2 — Additional file 2. [file 13063_2022_6672_MOESM2_ESM.zip › ANNEX3~2R1.PDF]

MRC/UVRI and LSHTM Uganda Research Unit

MRC Medical Research Council | Uganda Virus Research Institute | LONDON SCHOOL of HYGIENE &TROPICAL MEDICINE

Information and assent form for male students in the secondary schools invited to participate in the MENISCUS trial

**Project title:**	Menstrual health interventions, schooling and mental health symptoms among Ugandan students (MENISCUS): a school-based cluster-randomised trial
**Funder:**	UK Joint Global Health Trials (Medical Research Council-Department for International Development-Wellcome Trust) Grant # MR/V005634/1
**Research Site:**	Wakiso and Kalungu Districts<br>C/o MRC/UVRI and LSHTM Uganda Research Unit.<br>Plot 51-59, Nakiwogo Road<br>P O Box 49, Entebbe, Uganda<br>Tel: +256(0) 417 704000; (0)312 262910/1; (0)702 438487
**Principal Investigators:**<br><br><br><br><br>**Local Principal Investigator:**	**1. Prof Helen Weiss,**<br>Professor of Epidemiology and Director of the MRC Tropical Epidemiology Group, London School of Hygiene and Tropical Medicine (LSHTM), UK<br>*Email: helen.weiss@lshtm.ac.uk*<br>**2. Prof Janet Seeley**<br>Professor of Anthropology and Health, London School of Hygiene and Tropical Medicine (LSHTM), UK<br>and Head of Social Science Programme, MRC/UVRI and LSHTM Uganda Research Unit.<br>*Email: janet.seeley@lshtm.ac.uk*
**Trial Manager:**	Dr. Catherine Kansiime,<br>MRC/UVRI and LSHTM Uganda Research Unit<br>*Email:Catherine.Kansiime@mrcuganda.org*

Summary (What you should know about this study):

- The aim of the study is to assess whether a school-based intervention focused on improving management of menstrual periods improves education, health and well-being outcomes among girls in secondary school in Wakiso and Kalungu districts in Uganda.
- This document explains the purpose of this study and what you will be asked to do if you agree to participate.
- Your participation is completely voluntary. You have the right to take part in the study or to agree to take part now and change your mind later.
- Whatever you decide will not affect your regular healthcare and support.
- Please review this form carefully. Ask any questions before you make a decision.

You will be given a copy of this form to keep.

UVRI-RESEARCH ETHICS COMMITTEE
VALID UNTIL
26 MAY 2023
APPROVED
P. O. BOX 49, ENTEBBE (U)

Part I: Information about this study

Introduction

The MENISCUS trial is led by scientists at the London School of Hygiene & Tropical Medicine, MRC/UVRI and LSHTM Uganda Research Unit, with our partner WoMena Uganda.

We are carrying out research to guide our secondary schools to identify practical ways of helping girls to become and stay healthier and complete studying at school through improved management of menstrual periods. We have received permission to conduct this research from your school administration, the district, the Ministry of Education and Sports, and the Research Ethics Committees of the UVRI, LSHTM and Uganda National Council of Science and Technology (UNCST).

We invite you to be part of this research. It is optional for you to choose whether or not you want to participate in this research.

Please feel free to ask us questions now or later using our contact information which is indicated below. We will take time to explain to you.

Purpose

The purpose of the MENISCUS study is to see whether a health promotion intervention in secondary schools improves menstrual health (i.e. how girls manage their periods safely and confidently). We want to learn whether the package is likely to improve menstrual health knowledge and attitudes towards periods among boys, and education, health and well-being outcomes among girls. If the intervention is successful, it could be introduced in other schools in Uganda

Selection

We request you to participate in this research because we believe that you are a male secondary school student starting Form 2 in one of 60 schools chosen for this study. A few boys in each school have been selected at random to participate in the study.

Voluntary Participation

It is optional for you to participate in this research. You can choose to say no. That decision shall not affect any services that you and your family receive at the secondary school and/or health facilities. You can ask as many questions as you like and we shall be available to answer them. You don't have to decide today. You can think about it and tell us what you decide later.

Procedures

There are 60 schools in the trial. Of these, half (30) will be randomly selected to receive the MENISCUS intervention. In these 30 schools, pupils in Form 2 at the start of 2022 will receive education about puberty and menstruation and improvements to school toilet facilities. The intervention will be available throughout 2022. The schools which do not receive the MENISCUS intervention in 2022 will have the opportunity to receive it in 2023.

We request you to participate in two self-completed questionnaires (~15 minutes). Some boys will also be asked to participate in an individual interview with a researcher and/or a group discussion (60 minutes). If you are selected for an interview or discussion, we will ask your parent for their consent for you to participate in these.

1) <u>Questionnaires at the start and end of the study (about 15 minutes)</u>

You will be asked to complete a short questionnaire at the beginning of the study (~2022) and at the end of the study (~2023). The data are on your knowledge and attitudes towards menstruation. The

UVRI-RESEARCH ETHICS COMMITTEE
VALID UNTIL
26 MAY 2023
APPROVED
P. O. BOX 49, ENTEBBE (U)

data will be collected using tablet computers and our trained researchers will explain to you how to use these to complete the questionnaires. If you do not wish to answer some of the questions, you may skip them and move to the next question. In some cases, you may be asked to answer the questions using a paper questionnaire instead. These data will be entered by trained research assistants into a password-protected electronic database. The trained researchers and research assistants will maintain safe custody of paper forms with identification data, including contact details in lockable cabinets and/or cupboards. This will be done to preserve privacy and confidentiality of participants. Nobody will be able to identify you from the research reports, presentations and publications.

2) Group discussions and/or interviews among students (of about 60 minutes):
Some boys in the intervention schools will be asked to take part in a discussion with a small group of other boys and/or individual interviews, on school premises. They will be guided by our trained adolescent-friendly researchers who will be formally introduced to the school authorities and will wear personal identity cards. The researcher will ask about your perceptions of the intervention, including puberty education sessions, and attitudes of yourself and the community towards menstruating girls. The discussions will be tape-recorded and the tapes will be kept securely in lockable cabinets/cupboards at our research office. The information recorded is confidential, and no one else except the researchers or other ethical eligible person(s) such as the ethics review committees with regulated access to the tapes will be allowed to listen to the tapes. Nobody's name will be mentioned on the tapes and you will not be able to be identified.

Risks and discomfort: Is this bad or dangerous for you?
We are asking you to share with us some personal information (e.g. about sexual and reproductive health like male puberty, genital hygiene and attitudes towards menstruating girls). You may feel uncomfortable talking about some of the topics.

Benefits: Is there anything good that happens to you?
You may benefit from some of the intervention elements, such as improvements to school toilet/wash facilities and puberty training. Taking part in this research may also remind you to think deeply about your health and your future.

Reimbursements: Will you get anything for being in the research?
You will not be paid to take part in this research. However, you will be given a pen, a hardcover note book and a soft drink to compensate for your time and effort.

Confidentiality: Is anybody going to know about this?
We will not tell other people that you were involved in this research. We shall not share personal information that identifies you to anyone who does not work in this research. Any information about you will have a study number on it instead of your name. However, your data may be seen by auditors.

Sharing the Findings: Will you be told the study results?
When this research is completed, we shall inform you, your peers and parents/guardians about the results obtained from the trial. The results will never be reported in a way that allows anyone except members of the research team to know what you specifically told us or any of the individual results we obtained from you. We will also shall share the research results with authorities at the school, district and national levels, including what we have learnt.

Afterwards, we will be telling other people, scientists, health workers and others, what we found. We will do this by writing and sharing reports and by going to meetings with people who are interested in this work. The research findings will be published in international science journals and electronic websites so that other people may learn from us. Data may also be made available in the public domain

UVRI-RESEARCH ETHICS COMMITTEE
VALID UNTIL
26 MAY 2023
APPROVED
P. O. BOX 49, ENTEBBE (U)

via the London School of Hygiene and Tropical Medicine data repository. This means that it may be used for further analyses. The data will be anonymised i.e. it cannot be linked to you.

Who to Contact: Who can you talk to or ask questions about this study?
You can ask us questions now or later by telephone, e-mail, post or at the physical addresses indicated on the assent/consent form to be given to you. If you are nearby, you can come and see us.

You can contact any of the following about this research:

Dr. Catherine Kansiime, MENISCUS trial Project Lead.
Email: Catherine.Kansiime@mrcuganda.org; Phone number +256 702438487

If you have any questions, complaints or concerns about your rights as a person involved in this research, please contact: UVRI Research Ethics Committee: Phone number +256 0414 321962 or +256 716 321962

You may re-watch the video about the study at this link:
https://www.lshtm.ac.uk/research/centres-projects-groups/meniscus#resources

UVRI-RESEARCH ETHICS COMMITTEE
VALID UNTIL
26 MAY 2023
APPROVED
P. O. BOX 49, ENTEBBE (U)

PART 2: ASSENT (VERSION 1.3, MARCH 2022)

By signing below, I assent to participate in the study as described above, including:

- To self-complete the two study questionnaires
- To participate in a group discussion or interview if selected for these activities
- For all anonymised data collected to be used as part of the research and shared with other researchers

My questions concerning this study have been answered by ………………………………..

**Please read each question below**	**Please circle all you agree with:**	
Have you read (or had read to you) information about this project?	Yes	No
Has somebody else explained this project to you?	Yes	No
Do you understand what this project is about?	Yes	No
Have you had any questions answered in a way you understand?	Yes	No
Do you understand that it is ok to stop taking part at any time without giving a reason?	Yes	No
Are you happy to take part in the study? [ASSENT]	Yes	No

Student study number (IDNO): |__|__|__|__|__|__|__|__|

Name of student: ________________________________

Signature of student: ____________________

Date of interview (IDATE): |__|__|/|__|__|/|__|__|__|__|
dd / mm / yyyy

To be completed by the researcher
I confirm that the individual has given assent freely.

Name of researcher: ______________________ Date:|__|__|/|__|__|/|__|__|__|__|
dd / mm / yyyy

Signature: ___________________

UVRI-RESEARCH ETHICS COMMITTEE
VA[illegible] UNTIL
26 MAY 2023
A[illegible]ED
P. O. BOX 49, ENTEBBE (U)
