# Supplementary material for: Menstrual health interventions, schooling, and mental health problems among Ugandan students (MENISCUS): study protocol for a school-based cluster-randomised trial
Source: Trials. 2022 Sep 7;23:759. doi: 10.1186/s13063-022-06672-4 (PMC9449307; doi:10.1186/s13063-022-06672-4)
Supplement: Supplementary file 2 — Additional file 2. [file 13063_2022_6672_MOESM2_ESM.zip › ANNEX3~3R1.PDF]

Olupapula oluliko Amawulire agasaba Abaana abalenzi mu masomero ga siniya Okukkiriza Okwetaba mu kunoonyereza Kwa MENISCUS

**Project title:**	Menstrual health interventions, schooling and mental health symptoms among Ugandan students (MENISCUS): a school-based cluster-randomised trial
**Funder:**	UK Joint Global Health Trials (Medical Research Council-Department for International Development-Wellcome Trust) Grant # MR/V005634/1
**Research Site:**	Wakiso and Kalungu Districts<br>C/o MRC/UVRI and LSHTM Uganda Research Unit<br>Plot 51-59, Nakiwogo Road<br>P O Box 49, Entebbe, Uganda<br>Tel: +256(0) 417 704000;(0)312 262910/1; (0)702 438487
**Principal Investigators:**	**1. Prof Helen Weiss,**<br>Professor of Epidemiology and Director of the MRC Tropical Epidemiology Group, London School of Hygiene and Tropical Medicine (LSHTM), UK<br>*Email: helen.weiss@lshtm.ac.uk*
**Local Principal Investigators:**	**2. Prof Janet Seeley**<br>Professor of Anthropology and Health, London School of Hygiene and Tropical Medicine (LSHTM), UK<br>and Head of Social Science Programme, MRC/UVRI and LSHTM Uganda Research Unit<br>Plot 51-59, Nakiwogo Road<br>*Email: janet.seeley@lshtm.ac.uk*
**Trial Manager:**	Dr. Catherine Kansiime,<br>MRC/UVRI and LSHTM Uganda Research Unit<br>*Email:Catherine.Kansiime@mrcuganda.org*

Mu bufunze (By'olina okumanya ku kunoonyereza kuno):

- Ekigendererwa ky'okunoonyereza kwa MENISCUS kwe kumanya oba nga kinayambako mu kulongosa ebyekusoma, obubonero obulabirwako eby'obulamu ebikwata kubwongo, okutumbula engeri abaana abawala jebasobola okubeera obulungi nga bali mu nsonga z'ekikyala awamu n'embeera y'obulamu bwabwe mu mamasomero ga siniya mu wakiso ne kalungu mu Uganda
- Ekiwandiiko kino kinnyonnyola ekigendererwa ky'okunoonyereza kuno ne ky'onasabibwaokukola singa onooba okkirizza omwanawo okukwetabamu.
- Okw'etaba kw'omwana mu kunoonyereza kuno kwa kyeyagalire. Dembe lye okukwetabamu, oba okukwetabamu oluvannyuma n'akuvaamu.
- Kyonna ky'anaaba asazeewo tekijja kukosa ngeri jafunamu bujjanjabi wadde obuyambi.
- Soma ekiwandiiko kino n'obwegendereza era obuuze ekibuuzo kyonna ky'oyagala nga tonasalawo.

Ojja kuweebwa kopi ku kiwaandiiko kino

UVRI-RESEARCH ETHICS COMMITTEE
VALID UNTIL
28 MAY 2023
APPROVED
P. O. BOX 49, ENTEBBE (U)

Ekitundu ekisooka: Ebikwata ku kunoonyereza kuno.

Enyanjula (Introduction):

Okunoonyereza kwa MENISCUS ku kulembeddwamu ekitongole kya MRC/UVRI ne Tendekero lya London School of Hygiene and Tropical Medicine (LSHTM) nga bakolerera wamu n'ekitongole kya WOMENA Uganda.

Tukola Okunoonyereza kuno okulungamya amasomero ga Siniya okuzuula engeri ezisoboka ez'okuyamabamu abaana abawala okubeera abalamu n'okubeera ku somero obulunji nga bali mu nsonga z'ekikyala. Twafunye olukusa okukola okunonyereza kuno okuva kubakulu b'essomero, ekitongole ky'ebyenjigiriza n'emizannyo n'obukiiko obulondoola okunonyereza obwa UVRI, LSHTM ne UNCST.

Tukusaba okkirize okwetaba mu kunoonyereza kuno. Ddembe lyo okukkiriza oba obutakkiriza. Tujakusaba nemuzadde wo olukusa olukukiriza oketabamu. Tuyina okufuna olukusa okuva eri muzaddewo nawe.

Oli waddembe okutubuuza ekibuuzo kyonna ky'oyagala kati oba oluvannyuma ng'oyita ku email ne namba z'esimu eziragiddwa wa manga era tujja kutwala obuvunaanyizibwa tukunnyonnyole otegeere.

Ekigendererwa (Purpose):

Ekigendererwa ky'okunoonyereza kwa MENISCUS kwe kulaba oba nga enkola yokutumbula eby'obulamu mu mumasomero ga siniya enayambako mu kulongosa ensonga z'ekikyala (engeri abaana abawala jebasobola okubeera obulungi nga bali mu nsonga z'ekikyala) n'okumanya oba nga kinaayambako mu kulongosa eby'okusoma, eby'obulamu mubaana abawala awamu n'okumanya kwa baana abalenzi kubikwata kusonga za bakyala. Okunonyereza kunno bwekunaba kuvudemu ebirungi, kujja kutongozebwa mumasomera amalala mu Uganda.

Okulonda (Selection):
Tukusaba okwetaba mukunonyereza kuno kubanga oli omu kubaana abalenzi abali mu siniya ey'okubiri mu masomero enkaaga (60) agalondedwa okwetaba mukunonyereza kuno. Buli somero twalonzemu abalenzi abanetaba mukunonyereza kuno.

Okwetabamu kwa ky'eyagalile:
Okwetaba mu kunoonyereza kuno kwa kyeyagalire. Ggwe oba muzaddewo muli baddembe okugaana. Okusalawo obuteegatta mu kunoonyereza kuno tekijja kukosa gwe ne famileyo bye mulina kufuna ku somero wadde ewajjanjabirwa wonna. Oli wa ddembe okutubuuza ebibuuzo byonna era tuli beetegefu okubyanukula. Osobola obutasalawo kati, oli waddembe okusooka okukirowoozaako n'otubuulira oluvannyuma ky'onooba osazeewo.

Emitendera (Procedure)
Okunonyereza kuno kwetabidwamu amasomero nkaaga (60) nga amakumi assatu (30) kugo aganaba galondedwa bajja kufuna ettu lya MENISCUS. Mu masomero gano amakumi assatu(30), abayizi mu siniya 2 kutandikwa y'omwaka 2022 bajakusomsebwa ku nkyukakyuka ezibawo nga omwana avubuka, ensonga z'ekikyala n'okuterezamu ku kabuyonjo z'essomero. Ettu lino lijakugabibwa mu masomero mumwaka gwa 2022 gwonna. Ate ago amasomero aganaba tegafunye ettu lino, bajja kuba n'omukisa okufuna ettu lyelimu mu 2023.

UVRI-RESEARCH ETHICS COMMITTEE
VALID UNTIL
26 MAY 2023
APPROVED
P. O. BOX 49, ENTEBBE (U)

Tukusaba wetabe mu kwesomera n'okuddamu ebibuuzo (edakika 15). Abalenzi abamu mujakusabibwa okwanukula ebibuuzo oba okukubaganya ebirowoozo okwawamu (60 minutes) . Bwonaba olondedwa okwetaba mukukubaganya ebirowoozo oba okwanukula ebibuzo, tugya kusooka kufuna olukusa okuva eri muzadde wo.

1) **Okwanukula ebibuuzo by'okumpampula (eddakiika 15).**
Ojakusabibwa okujjuza empapula z'ebibuuzo ku ntandikwa (~2022) ne ku nkomerero y'okunoonyereza kuno (~2023) nga zijja ku muweebwa nga biteredwa ku bukompyuta obutono era abakola ku kunoonyereza kuno bajakumuyambako okumunyonyola engeri yokujuzamu ebibuzo. Ekibuuzo kyonna ky'anaawulira nga tayagala ku kiddamu ajja kuba wa ddembe okukireka n'agenda ku kilala. Abakola ku kunoonyereza kuno baakukuuma ebiwandiiko byonna ebikwata ku beetabye mu kunoonyereza kuno nga bya kyama era bya kusibirwa mu kabada ko n'okuyingizibwa mu Kompyuta esibibwe n'ekigambo eky'ekyama (Password).

Abakola ku kunonyereza kuno bakukuuma ebiwandiiko byonna ebikwatta kubetabye mukunonyereza kuno nga byakyama era byakusibibwa mu kabadda . Naye nga tewali ngeri yonna mukwogera ebinava mukunonyereza kuno byewatubulira nga omuntu webigya kulabikira.

2) **Okukubaganya Ebirowoozo okwawamu / Okwanukukula ebibuuzo (Edakiika 60):**
Abalenzi abamu okuva mumasomero amakumi assatu (30) aganaba gafunye ettu lya MENISCUS mujakusabibwa okwetaba mukukubaganya ebirowoozo n'abalenzi banabwe abalala oba okuddamu ebibuuzo byasekinoomu nga muli kussomero. Mujakuyambibwako abavubuka abakola kukunonyereza kuno abanabera bayanyuddwa ku masomero gamwe era bajakutambulanga nebibakwattako (Identity cards). Ojakusabibwa okuwa endowooza ye ku ttu lya MENISCUS. Okukubaganya ebirowoozo kwa kubeera ku Somero era kujja kwatibwa ku butambi era obutambi obwo bwa kusibirwa mu kabada ku UVRI. Ebinaakwatibwa ku butambi bya kukumibwa nga bya kyama era tewali ajja ku biwulirako okujjako abakola ku kunoonyereza n'abalala abakkirizibwa mu mateeka agafuga okunoonyereza nga abajja okubissa mu buwandiike (Transicribers), abatadde ensimbi mu mulimu guno ko n'obukiiko obulondoola n'okulabirira okunonyereza. Era tewali linnya lya muntu yenna liggya kw'ogerwako mu butambi

Obutyabaga n'okuteteganyizibwa:
Tujja kukubuuza ebikukwatako ng'omuntu n'ebikwata ku bulamubwo obw'ekyama okugeza , okuvubuka , obuyonjo mu bintundu by'ekyama n'endowoza ku abaana abawala abagenda munsonga zabwe ezekikyala ekiyinza okukuleetera obutawulira bulungi nga oby'ogerako.

Okuganyurwa (benefits):
Ojakuganyurwa mukufuna ettu omui okuterezamu ku kabuyonjo ze ssomero n'okusomesebwa ku nkyukanyuka ezenjawulo ezibawo nga akula (puberty education). Ate era okwetabakwo mu kunoonyereza kuno kujja kuyamba okulowooza enyo ku bulamubwo n'ebiseera byo eby'omumaaso.

Okusasulwa:
Tojja kusasulwa olw'okwetaba mu kunoonyereza kuno, mpozzi ojja kuweebwayo ka peni n'akatabo akeddiba eggumu ak'enjawulo,n'akokunywa akagonvu olw'obudde bwo ne kaweefube gw'onoba otaddemu.

Emmizi (Confidentiality):
Tewali gwe tujja kubuulirako nti weetabyemu kunoonyereza kuno. Tewali muntu yenna atakola mu kunoonyereza kuno gwe tujja kubuulirako ku bikwatako era tujja kuba tukozesa namba (study number) mu kifo ky'e linnyalyo. Wabula ebimukwatako biyinza okulabibwako ba Auditor.

UVRI-RESEARCH ETHICS COMMITTEE
VALID UNTIL
2 8 MAY 2023
APPROVED
P. O. BOX 49, ENTEBBE (U)

Okutegeezebwa ebinaazuulibwa mu kunoonyereza:
Okunoonyereza kuno nga kuwedde gwe ne bayizi banno muja kutegeezebwa ebinaaba bizuuliddwa era tujja kubitegeeza n'abazaddde, abakulira essomero lino ko aba Munisipaali n'egwanga lyonna okutwalira awamu. Tujja kubitegeeza n'abantu abalala omuli ba nasayansi, abakola ku by'obulamu, n'abantu abalala. Kino tujja kikola nga tuyita mu kuwandiika zi lipooti, n'okusisinkana bonna be kikwatako.

Ebinaava mu kunoonyereza kuno era bya kuteekebwa mu butabo (journals) bwa sayansi obw'ensi yonna ko n'emikutu ja yintaneti abantu abalala basobole okutuyigirako.. Ebivudde mukunonyereza kuno era biyinza okutekebwa ku mukutu gwa London School of Hygiene and Tropical medicine abantu abalala gyebayinza okubisanga. Kino kitegeza nti tuyinza okudamu okwekenenya ebinaba bivudde mukunonyereza naye nga tewali ngeri yonna mukwogera ebinava mukunonyereza kuno bye watubulira nga omuntu webijja kulabikira.

Okwebuuza:Ani gw'oyinza okw'ogerako naye oba okubuuza ebikwata ku kunoonyereza kuno?

Oli waddembe okubuuza ekibuuzo kyonna kati oba je bujja ng'oyita ku simu oba ku e-mail oba okujja ku MRC/UVRI kwe nnyini n'otulaba mu buntu.

Osobola okutuukirira:

Dr. Catherine Kansiime
MENISCUS trial Project Lead
Email: catherine.kansiime@mrcuganda.org
Essimu: +256 702438487

Bwoba olina ekibuuzo oba okwemulugunya ku ddembe ly'omwana wo ku by'okwetabakwo mu kunoonyereza kuno tukirira akakiiko ka UVRI akalondoola n'okulabirira okunonyereza ku simu +256 0414 321962 oba +256 716321962.

UVRI-RESEARCH ETHICS COMMITTEE
VALID UNTIL
26 MAY 2023
APPROVED
P. O. BOX 49, ENTEBBE (U)

EKITUNDU 2: OKUKKIRIZA (VERSION 1.2, AUGUST 2021)

Nga ntekako omukono wamanga, nzikiriza okwetaba mukunonyereza kuno omuli;

- Okwesomera n'okwanukula ebibuzo ebyemirundi ebbiri
- Okwetaba mu kukubaganya ebiroowozo okwawamu oba okwa sekinoomu singa naba nondedwa okwetabamu
- Ebinava mukunonyereza kuno okukozesebwa n'okutegezako abanonyereza abalala naye nga ebikwata ko tebija kumanyibwa.

Ebibuuzo byange ebikwata ku kunoonyereza kuno byanukuddwaErinnya____

**Soma era Oddemu ebibuzo bino**	**Saza kwebyo byonaba osazewo**	
Osomye oba osomedwa ebikwata ku kunonyereza kuno?	Yee	Nedda
Waliwo omuntu omulala yenna akunyonyode ku kunonyereza kuno?	Yee	Nedda
Otegedde bulungi okunonyereza kuno kyekukwattako?	Yee	Nedda
Ebibuuzo byo ku kunonyereza kuno bididwamu bulungi?	Yee	Nedda
Otegedde bulungi nti oli wadembe okuva mukunonyereza kuno ?	Yee	Nedda
Oli musanyufu okukiriza okwetaba mukunonyereza kuno? [ASSENT]	Yee	Nedda

Student study number (IDNO): |__|__|__|__|__|__|__| School ID: |__|__|__|

Wandiika (Print) Amannya ______________________________

Omukono gwo'omuyizi (signature)_________________________

Date of consent (IDATE): |__|__|/|__|__|/|__|__|__|__|
dd / mm / yyyy

Wano wakujjuzibwa akola ku kunoonyereza

To be completed by the researcher
I confirm that the individual has given assent freely.

Name of researcher: ________________________ Date:|__|__|/|__|__|/|__|__|__|__|

dd / mm / yyyy

Signature: ______________________

The Parent/Guardian has signed an informed consent (Yes=1, No=2) |__|
_____ (initialled by researcher/assistant)

UVRI-RESEARCH ETHICS COMMITTEE
VALID UNTIL
26 MAY 2023
APPROVED
P. O. BOX 49, ENTEBBE (U)

UVRI-RESEARCH ETHICS COMMITTEE
VALID UNTIL
★ 26 MAY 2023 ★
APPROVED
P. O. BOX 49, ENTEBBE (U)
