# Supplementary material for: Menstrual health interventions, schooling, and mental health problems among Ugandan students (MENISCUS): study protocol for a school-based cluster-randomised trial
Source: Trials. 2022 Sep 7;23:759. doi: 10.1186/s13063-022-06672-4 (PMC9449307; doi:10.1186/s13063-022-06672-4)
Supplement: Supplementary file 2 — Additional file 2. [file 13063_2022_6672_MOESM2_ESM.zip › ANNEX3~4R1.PDF]

Olupapula oluliko Amawulire agasaba Abazadde oba Abavunanyizibwa ku baana Abawala, Abakulu b'amasomero n'abalala Abaakwasibwa obuvunanyizibwa bw'okulabirira abaana nga bazadde baabwe tebaliiwo okwetaba mu kunoonyereza kwa MENISCUS.

**Project title:**	Menstrual health interventions, schooling and mental health symptoms among Ugandan students (MENISCUS): a school-based cluster-randomised trial
**Funder:**	UK Joint Global Health Trials (Medical Research Council-Department for International Development-Wellcome Trust) Grant # MR/V005634/1
**Research Site:**	Wakiso and Kalungu Districts<br>C/o MRC/UVRI Uganda Research Unit on AIDS<br>Plot 51-59, Nakiwogo Road<br>P O Box 49, Entebbe, Uganda<br>Tel: +256(0) 417 704000; (0)312 262910/1; (0)752 731733
**Principal Investigators:**<br><br>**Local Principal Investigator:**	**1. Prof Helen Weiss,**<br>Professor of Epidemiology and Director of the MRC Tropical Epidemiology Group, London School of Hygiene and Tropical Medicine (LSHTM), UK<br>*Email: helen.weiss@lshtm.ac.uk*<br>**2. Prof Janet Seeley**<br>Professor of Anthropology and Health, London School of Hygiene and Tropical Medicine (LSHTM), UK<br>and Head of Social Science Programme, MRC/UVRI<br>*Email: janet.seeley@lshtm.ac.uk*
**Trial Manager:**	Dr. Catherine Kansiime,<br>MRC/UVRI and LSHTM Uganda Research Unit<br>*Email:Catherine.Kansiime@mrcuganda.org*

Mu bufunze (By'olina okumanya ku kunoonyereza kuno):

- Ekigendererwa ky'okunoonyereza kwa MENISCUS kwe kumanya oba nga kinayambako mu kulongosa ebyekusoma, obubonero obulabirwako eby'obulamu ebikwata ku by'obwongo, okutumbula engeri abaana abawala jebasobola okubeera obulungi nga bali mu nsonga z'ekikyala awamu n'embeela y'obulamu bwabwe mu masomero ga siniya mu wakiso ne kalungu mu Uganda
- Ekiwandiiko kino kinnyonnyola ekigendererwa ky'okunoonyereza kuno ne ky'onasabibwaokukola singa onooba okkirizza omwanawo okukwetabamu.
- Okw'etaba kw'omwana mu kunoonyereza kuno kwa kyeyagalire. Dembe lye okukwetabamu, oba okukwetabamu oluvannyuma n'akuvaamu.
- Kyonna ky'anaaba asazeewo tekijja kukosa ngeri jafunamu bujjanjabi wadde obuyambi.
- Soma ekiwandiiko kino n'obwegendereza era obuuze ekibuuzo kyonna ky'oyagala nga tonasalawo.

Ojja kuweebwa kopi ku kiwaandiiko kino

Ekitundu ekisooka: Ebikwata ku kunoonyereza kuno.

UVRI-RESEARCH ETHICS COMMITTEE
VALID UNTIL
2 6 MAY 2023
APPROVED
P. O. BOX 49, ENTEBBE (U)

Enyanjula (Introduction):
Okunoonyereza kwa **MENISCUS** ku kulembeddwamu ekitongole kya **MRC/UVRI** ne Tendekero lya **London School of Hygiene and Tropical Medicine (LSHTM)** nga bakolerera wamu n'ekitongole kya WoMena Uganda.

Tukola Okunoonyereza kuno okulungamya amasomero ga Siniya okuzuula engeri ezisoboka ez'okuyambamu abaana abawala okubeera abalamu n'okubeera ku somero obulungi nga bali mu nsonga z'ekikyala. Twafunye olukusa okukola Okunoonyereza kuno okuva eri abatwala e Somero lino, okuva ku disitirikiti, okuva mu ministry y'ebyenjigiliza n'emizanyo n'okuva mu bukiiko obulondoola okunoonyereza obwa MRC/UVRI ne LSHTM wamu ne National Council of Science and Technology.

Tukusaba okkirize omwanawo okwetaba mu kunoonyereza kuno. Ddembe lyo okumukkiriza oba obutamukkiriza. Bw'onamukkiriza okwetaba mu kunoonyereza kuno, n'omwana tujja kumusaba akkirize. Tulina okufuna olukusa okuva eri omuzadde n'omwana okusobola okwetabamu.

Oli waddembe okutubuuza ekibuuzo kyonna ky'oyagala kati oba oluvannyuma ng'oyita ku email ne namba z'esimu eziragiddwa wa manga era tujja kutwala obuvunaanyizibwa tukunnyonnyole otegeere.

Ekigendererwa:
Ekigendererwa ky'okunoonyereza kwa MENISCUS kwe kulaba oba nga enkola yokutumbula eby'obulamu mu mumasomero ga siniya enayambako mu kulongosa ensonga z'ekikyala (engeri abaana abawala jebasobola okubeera obulungi nga bali mu nsonga z'ekikyala) n'okumanya oba nga kinaayambako mu kulongosa eby'okusoma, eby'obulamu mubaana abawala awamu n'okumanya kwa baana abalenzi kubikwata kusonga za bakyala. Okunonyereza kunno bwekunaba kuvudemu ebirungi, kujja kutongozebwa mumasomera amalala mu Uganda.

Okulonda (Selection):
Tusaba omwanawo okwetaba mu kunoonyereza kuno kubanga muyizi muwala (Female student) owa siniya 2 mu limu ku masomero enkaaga agaalondeddwa okukoleramu okunonyereza kuno. Tukusaba kuba ggwe muzadde/alina obuvunaanyizibwa (Guardian) okkirize omwanawo.

Okwetabamu kwa kyeyagalile:
Okwetaba mu kunoonyereza kuno kwa kyeyagalire. Ggwe oba muwalawo muli baddembe okugaana. Okusalawo obuteegatta mu kunoonyereza kuno tekijja kukosa gwe ne famileyo bye mulina kufuna ku somero wadde ewajjanjabirwa wonna. Oli wa ddembe okutubuuza ebibuuzo byonna era tuli beetegefu okubyanukula. Osobola obutasalawo kati, oli waddembe okusooka okukirowoozaako n'otubuulira oluvannyuma ky'onooba osazeewo. Oli waddembe okukkiriza omukulu w'essomero oba omuntu yenna gw'oyagala okuteeka omukono ku biwaandiiko ebikkiriza omwanawo okwetaba mu kunoonyereza kuno ku lulwo singa onooba toosobole kubaawo.

Emitendera
Okunonyereza kuno kwetabidwamu amasomero nkaaga (60) nga amakumi assatu (30) kugo aganaba galondedwa bajja kufuna ettu lya MENISCUS. Mu masomero gano amakumi assatu(30), abayizi mu siniya 2 kuntandikwa y'omwaka 2022 bajakusomesebwa ku nkyukakyuka ezibawo nga omwana avubuka, ensonga z'ekikyala n'okuterezamu ku kabuyonjo by'essomero era bajakuba n'omukisa okwetaba mu katemba oba emizanyo nga bigwatagana n'ensonga z'ekikyala. Muwalawo ajatula ebibuuzo byamirundi ebbiri nga bikwatagana kwebyo byanaba ayigirizidwa mu misomo gyaffe. Ettu lijakugabibwa mu masomero mumwaka gwa 2022 gwonna. Ate ago amasomero aganaba tegafunye ettu lino, bajja kuba n'omukisa okufuna ettu lyelimu mu 2023.

UVRI-RESEARCH ETHICS COMMITTEE
VALID UNTIL
26 MAY 2023
APPROVED
P.O. BOX 49, ENTEBBE (U)

Tukusaba okkirize muwalawo okwetaba mu bino omuli ebigenda okukolebwa mu kunoonyereza kuno omuli ebibuzo byamilundi ebbiri byeyedilamu (eddakiika 40-60), okwejako ebikeberebwa (vaginal swab) n'okufuna ettu ly'ebikozesebwa munsonga zekikyala. Abawala abamu bajakusabibwa okwanukula ebibuuzo n'omunonyereza (Eddakiika 60) , okukubaganya ebirowoozo okwawamu (1-2 hours) oba okujjuza Dayale (Personal diary) ejja okumuweebwa asobole okulamba ennaku zabeera ku ssomero n'enaku zaabeera mu nsonga z'ekikyala, na buli ddi wafuna obuluminga ali munsonga.

1) **Okwanukula ebibuuzo by'okumpampula (eddakiika 40-60) n'okukozesa ebinaba bivudde mu bigezo.**

Muwalawo ajja kusabibwa okujjuza empapula z'ebibuuzo ku ntandikwa (quarter 4, 2021) ne ku nkomerero y'okunoonyereza kuno (quarter 1 2023) nga zijja ku muweebwa nga biteredwa ku bukompyuta obutono era abakola ku kunoonyereza kuno bajakumuyambako okumunyonyola engeri yokujuzamu ebibuzo. Ekibuuzo kyonna ky'anaawulira nga tayagala ku kiddamu ajja kuba wa ddembe okukireka n'agenda ku kilala. Abakola ku kunoonyereza kuno baakukuuma ebiwandiiko byonna ebikwata ku beetabye mu kunoonyereza kuno nga bya kyama era bya kusibirwa mu kabada ko n'okuyingizibwa mu Kompyuta esibibwe n'ekigambo eky'ekyama (Password).

Mu kunonyereza kwaffe, tujakukozesa obubonero bwanaba afunye mu bigezo byaffe. . Ebinava mu bigezo bino tebija kukosa byakusomakwe ku somero. Ebinaba bivudde mu bigezo bijakukozesebwa mukunonyereza kwokka era teri muyizi yenna , musomesa oba muntu kubimanyako.

Abakola ku kunonyereza kuno bakukuuma ebiwandiiko byonna ebikwatta kubetabye mukunonyereza kuno nga byakyama era byakusibibwa mu kabadda . Naye nga tewali ngeri yonna mukwogera ebinava mukunonyereza kuno byewatubulira nga omuntu webigya kulabikira.

2) **Okugaba ettu ely'ebikozesebwa mu nsonga z'ekikyala.**

Abawala bonna mu masomero amakuumi assattu (30) ku ago enkaaga (60) aganaba galondedwa bajja kuweebwa ettu ly'ebikozesebwa mu nsonga z'ekikyala nga mulimu obuwale obwomunda, akacuupa akaterekebwamu amazzi (ekendeza obulumi), ssabuni ne ka tawelo. Muwalawo ajakusabibwa okwetaba mumusomo ogunakulemberwa omusomesa oba omuyizi anaba atendekedwa abakugu mu nsonga z'ekikyala ku ngeri y'okukozesamu paadi ezoozebwa ne ziddamu ne zikozesebwa . Ajja kusabibwa okwetaba mu musomo ogunaakubirizibwa omusomesa oba omu ku bayizi banne omuwala anaaba atendekeddwa bakafulu mu nsonga z'ekikyala, ne paadi ezoozebwa ne ziddamu ne zikozesebwa. Bajja ku mulaga engeri y'okukozesaamu paadi ezo era bajja ku munnyonnyola buli kyanaaba ayagala okumanya ku nsonga eno. Ajja kusabibwa okukozesa paadi ezoozebwa ne ziddamu ne zikozesebwa okumala emyezi 12 ejinaddako bwanaaba takirinaako buzibu.. Bwanaba alina obuzibu bwafunye mu kukozesa paadi ezo, ajjakusabibwa okubitegeeza abakugu abakola ku kunoonyereza , omusomesa, mwana munne, omuzadde, omukuza, omusawo wesomero oba dokita akwasaganya ensonga mukunonyereza kuno.

Ettu era lijakuberamu bu voucher obunakozesebwa okufuna empeke ezikendeza ku bulumi nga omuwala ali munsonga eza Panadol oba Ibuprofen mukaaga(6) buli mwezi okuva ew'omusawo w'essomero oba omusomesa anaba alondedwa. Muwalawo wakkiriza okwetaba mukunonyereza kuno, sikyatekwa okukozesa paadi ezo.

Abawala mumasomero amalala amakumi assatu bajjakuwebwa ettu lino nga okunonyereza kuno kuwedde.

3) **Okujuzamu Dayale okulaba enaku muwalawo zanabera ku ssomero ne zanabera nga mu nsonga z'ekikyala awamu n'obulumi bw'ayinza okufuna nga ali mu nsonga z'ekikyala**

Abawala abamu abanaba bakiriza okwetaba mukunonyereza kuno ajakusabibwa okujuza akatabo ka dayale kubikwatagana buli ddi lwabera ku somero, ddi lwabeera munsonga zekikyala na buli ddi

UVRI-RESEARCH ETHICS COMMITTEE VALID UNTIL 26 MAY 2023 APPROVED P. O. BOX 49, ENTEBBE (U)

wafuna obulumi nga ali munsonga. Dayale eno ejakujuzibwa okumala sabiiti 12 okuva kunkomerero y'omwaka 2022. . Abakola kukunonyereza kwa MENISCUS bajakuyambako muwalawo nga bamusomesa engeri gy'ayinza okujuzamu akatabo ako. Ebyo byonna byanaba ajuza mukatabo kano, bijakuyingizibwa mu byuma kalimagezi ku buli nkomerero ya tamu nga obutabo bukunganyizidwa

4) **Okukubaganya Ebirowoozo okwawamu / Okwanukukula ebibuuzo (1-2 hours)**:
Abawala abamu okuva mumasomero amakumi assatu (30) aganaba gafunye ettu lya MENISCUS bajakusabibwa okwetaba mukukubaganya ebirowoozo n'ebawala banabwe abalala oba okuddamu ebibuuzo byasekinoomu nga bali kussomero. Bajakuyambibwako abavubuka abakola kukunonyereza kuno abanabera bayanyuddwa ku masomero era bajakutambulanga nebibakwattako (Identity cards). Muwalawo ajakusabibwa okuwa endowooza ye ku ttu lya MENISCUS na ngeri ki jebimuyambyemu nga ali munsonga z'ekikyala era bino bijakubera ku ssomero. Okukubaganya ebirowoozo kwa kubeera ku Somero era kujja kwatibwa ku butambi era obutambi obwo bwa kusibirwa mu kabada ku UVRI. Ebinaakwatibwa ku butambi bya kuumibwa nga bya kyama era tewali ajja ku biwulirako okujjako abakola ku kunoonyereza n'abalala abakkirizibwa mu mateeka agafuga okunoonyereza nga abajja okubissa mu buwandiike (Transicribers), abatadde ensimbi mu mulimu guno ko n'obukiiko obulondoola n'okulabirira okunonyereza. Era tewali linnya lya muntu yenna liggya kw'ogerwako mu butambi.

5) **Obubonero bw'obukyaffu mu bukyala n'okwegyako ebikeberebwa (vaginal swabs)**
Mukuddamu ebibuzo kunkomerero y'okunonyereza, muwalawo ajakubuzibwa ku ngeri gy'ayonjamu ebitundu bye eby'ekyaama n'okumanya oba ayinamu obubonero bwonna nga okusiyibwa n'okulumiziba nga afuuka. Bwanaba atubulidde nti ayinamu kububonero buno, ajakusindikibwa ku dwaliro afune obujanjabi. Muwalawo wanatubulira nti afuna okusiyibwa n'okulumizibwa nga afuuka, tujamusaba atuwe omusulo gwe gukeberebwemu obulwadde bwa UTI.

Ajakusabibwa okutuwa swabu 2 kunkomerero yokunonyereza ezinakeberebwamu obuwuuka obukyaffu obubeera mubukyala (Bacterial Vaginosis) ne Candida. Obulwadde buno tebusasanyizibwa lwa kwegatta na basajja era omuntu okuba nabwo tekitegeza nti yegatta nabasajja. Muwalawo ajakutendekebwa ku ngeri gyalina okukozesa ebikebera endwadde eno. Swabu zijakusindikibwa mu laboratory yaffe era emu kuzo ejakukeberebwamu obuwuka obukyaffu obusangibwa mu bukyala ate endala ejakutelekebwa ekeberebwe mu biseera eby'omumaaso. Kiyinza okutwetagisa okusindika sampo ey'okubiri ebweru wegwanga okwongera okwekenenya obuwuka obwenjawulo obusangibwa mu bukyala. Tetuja kuwa muwalawo ebinava mukukebera swabu naye ajakufuna obujanjabi okuva kudwaliro bwanaba ayinamu obubonero bwonna obulaga nti alina obulwadde.

Lwaki sampo emu ejakusindikibwa ebweru we gwanga?
Sampo ey'okubiri ejakwetagibwa okwongera okwekenenya kitusobozese okumanya obuwuka obwenjawulo obusangibwa mu bukyala. Kino kyetagisa ebyuuma ebyamanyi byetutalina wano mu Uganda. Sampo zino tezijakuwandikibwako manya gamuntu wabula tujakukozesa enamba enekusiifu.

Obutyabaga n'okuteteganyizibwa: Kino kibi oba kya bulabe eri muwalawo?
Tujja kubuuza muwalawo ebimukwatako ng'omuntu n'ebikwata ku bulamubwe obw'ekyama omuli n'okumanya engeri gyeyeyisamu nga ali munsonga ze ez'ekikyala. Ayinza obutawulira bulungi nga aby'ogerako. Ajakusomesebwa oba okuyambibwako abakugu abayina obumanyirivu mu kuyigiriza abaana abawala okukozesa paadi ezoozebwa ne ziddamu nezikozesebwa. Era ajakusomesebwa kungeri y'okwoza n'okwanika paadi zino, engeri y'okumanya obubonero obulaga obukyaffu nani gwe bayinza okutukirira. Bwanaba tagobeledde ebimusomesedwa waliwo akatyabaga kokufuna obulwadde oba okusiyibwa. Wabula wewaberawo akatyabaga konna akaamanyi mukukozesa paadi ezozebwa muwalawo ajakubera waddembe okutukirila omusawo w'esomero bwaba nga omusawo w'esomero

UVRI-RESEARCH ETHICS COMMITTEE
VALID UNTIL
26 MAY 2023
APPROVED
P. O. BOX 49, ENTEBBE (U)

tasobola kumuyamba munsonga eyo ajakusindikibwa eri omusawo waffe .Muwalawo asabibwa okwogera amangu eri omusawo singa aba afunye obuzibu bwonna mu kukozesa paadi zino.

Muwalawo ayinza obutawulira bulungi nga ayanukula ebimu ku bibuzo ebikwatagana n'engeri gye yeyonjamu oba okusabibwa okwegyako ebikeberebwa. Okusobola okuziyiza kino, ajakuddamu ebibuzo ebinababiteredwa ku ka computer nga abyesomera era nabyediramu era tujakutekako ne katooni oba ebifananyi ebinamusobozesa okumanya ekikula kye, n'engeri gyayinza okufunamu sampo yebikeberebwa. Okwejako sampo tekiyina buzibu bwonna kyekuleta eri muwalawo era ne swabu ezikozesebwa ntono nyo mu sayizi, tezisobola kuleta buzibu bwonna mubukyala bw'omwana.

Muwalawo ajakuwebwa omukisa okubuuza byonna n'okwogera kwebyo ebyekuusa ku kikula ky'abakyala n'okugenda munsonga (menstruation). Amawulire ganno gajamuyamba okukola okusalawo okutuufu kungeri gyeyeyisamu nga ali munsonga. Ajakuwebwa paadi ezozeebwa ne ziddamu nezikozesebwa okumuyambako nga ali munsonga z'ekikyala.

Okuganyurwa (benefits): Waliwo engeri muwalawo gyanaganyurwamu?
Asobola okuganyurwa mukufuna ettu erinaba ligabidwa (menstrual kit) engeri enamusobozesa okubera obulungi nga ali munsonga z'ekikyala. Okufuna empeke ezitta obulumi obuleetebwa ensonga z'ekikyala era ne dayale egenda okumuweebwa nayo ejja kusobola okumuyamba okumanya lwanaddamu okugenda mu nsonga asobole okukyetegekera. Okuddamu ebibuuzo ku ndwadde z'omubukyala n'okuwayo sampo z'omusulo kijakumuyamba okufuna obujanjabi singa anaba asangidwamu obubonero bwendwadde. Tajja kusasulwa olw'okuwayo ebikeberebwa (swab) wabula okwetabaakwe mu kunoonyereza kuno kusobola okutuyamba, okuyamba amasomero, amalwaliro, n'abavunaanyizibwa ku byenjigiriza okuzuula amawulire (information) n'obuweereza (services) bye mwetaaga. Tusuubira nga kino kijja kuyamba be kikwatako okukola ku byetaago byabwe mu ngeri esinga okuba ennunji eyo jebujja.
Ate era okwetabakwe mu kunoonyereza kuno kujja ku muyamba okulowooza ennyo ku bulamubwe n'ebiseera bye eby'omumaaso.

Okusasulwa: Muwalawo anaasasulwa olw'okwetaba mu kunoonyereza kuno?
Ojakuwebwayo omutwalo gumu (10,000/=) olw'obudde bwo. Muwalawo tajja kusasulwa olw'okwetaba mu kunoonyereza kuno, mpozzi ajja kuweebwayo ka peni n'akatabo akeddiba eggumu ko n'akokunywa akagonvu olw'obudde bwe ne kaweefube gw'anaaba ataddemu.

Emmizi (Confidentiality): Ebintu bino binaamanyibwako abantu abalala?
Tewali gwe tujja kubuulirako nti muwalawo yeetabye mu kunoonyereza kuno. Tewali muntu yenna atakola mu kunoonyereza kuno gwe tujja kubuulirako ku bimukwatako era tujja kuba tukozesa namba (study number) mu kifo ky'e linnyalye. Wabula ebimukwatako biyinza okulabibwako ba Auditor.

Okutegeezebwa ebinaazuulibwa mu kunoonyereza: Onootegeezebwa ebinaazuulibwa mu kunoonyereza kuno?
Okunoonyereza kuno nga kuwedde tujakutegeza muwalawo, bayizi banne namwe abazadde ebinaaba bizuuliddwa era teri wekija kulabikira oba kulaga nti gwe watuwa amawulire ago okugyako abo abakola ku kunonyereza kuno okumanya endowooza ze zeyaatuwa..
Oluvanyuma , tujja kubitegeeza n'abantu abalala omuli ba nasayansi, abakola ku by'obulamu, n'abantu abalala nga tubategeza nebyo byetunaba tuyize nga tukola okunonyereza. Kino tujja kikola nga tuyita mu kuwandiika zi lipooti, n'okusisinkana bonna be kikwatako.
Ebinaava mu kunoonyereza kuno era bya kuteekebwa mu butabo (journals) bwa sayansi obw'ensi yonna ko n'emikutu ja intaneti abantu abalala basobole okutuyigirako. Ebivudde mukunonyereza kuno era biyinza okutekebwa ku mukutu gwa London School of Hygiene and Tropical medicine abantu abalala gyebayinza okubisanga. Kino kitegeza nti tuyinza okudamu okwekenenya ebinaba bivudde

UVRI-RESEARCH ETHICS COMMITTEE
VALID UNTIL
2 8 MAY 2023
APPROVED
P. O. BOX 49, ENTEBBE (U)

mukunonyereza naye nga tewali ngeri yonna mu kwogera ebinaava mu kunoonyereza kuno muwalawo byeyatubulira ng'omuntu we bijja kulabikira.

Okwebuuza: Ani gw'oyinza okw'ogerako naye oba okubuuza ebikwata ku kunoonyereza kuno?
Oli waddembe okubuuza ekibuuzo kyonna kati oba je bujja ng'oyita ku simu oba ku e-mail oba okujja ku MRC/UVRI kwe nnyini n'otulaba mu buntu.

Osobola okutuukirira:

Dr. Catherine Kansiime
MENISCUS trial Project Lead
Email: catherine.kansiime@mrcuganda.org
Essimu: +256 702438487

Bwoba olina ekibuuzo oba okwemulugunya ku ddembe ly'omwana wo ku by'okwetabakwo mu kunoonyereza kuno tukirira akakiiko ka UVRI akalondoola n'okulabirira okunonyereza ku simu +256 0414 321962 oba +256 716 321962

UVRI-RESEARCH ETHICS COMMITTEE
VALID UNTIL
2 8 MAY 2023
APPROVED
P. O. BOX 49, ENTEBBE (U)

EKITUNDU 2: OKUKKIRIZA (VERSION1.3 November 2021)

Nga ntekako omukono wamanga, nzikiriza muwala wange okwetaba mukunonyereza kuno omuli;

- Okwesomera n'okwanukula ebibuzo ebyemirundi ebbiri
- Okufuna ettu lye bikozesebwa munsonga
- Okwejako ebikeberebwa bya mirundi ebbiri nga ekimu ku byo kyakuterekebwa era kisobola n'okutwalibwa ebweru we gwanga.
- Okwetaba mu kukubaganya ebiroowozo okwawamu oba okwa sekinoomu n'okujuzamu dayale singa anaba alondedwa okwetabamu
- Ebinava mukunonyereza kuno okukozesebwa n'okutegezako abanonyereza abalala naye nga ebikwata ku muwala wange tebija kumanyibwa.

Ebibuuzo byange ebikwata ku kunoonyereza kuno byanukuddwa Erinnya__________________

Soma era Oddemu ebibuzo bino	Saza kwebyo byonaba osazewo	
Osomye oba osomedwa ebikwata ku kunonyereza kuno?	Yee	Nedda
Waliwo omuntu omulala yenna akunyonyode ku kunonyereza kuno?	Yee	Nedda
Otegedde bulungi okunonyereza kuno kyekukwattako?	Yee	Nedda
Ebibuuzo byo ku kunonyereza kuno bididwamu bulungi?	Yee	Nedda
Otegedde bulungi nti oli wadembe okuva mukunonyereza kuno ?	Yee	Nedda
Oli musanyufu okukiriza muwalawo okwetaba mukunonyereza kuno? [CONSENT]	Yee	Nedda

Student name: ________________________ School ID number |__|__|__|

Wandiika (Print) Amannya g'omuzadde/avunaanyizibwa ku mwana/Eyaweebwa

UVRI RESEARCH ETHICS COMMITTEE
VALID UNTIL
★ 26 MAY 2023 ★
APPROVED
P. O. BOX 49, ENTEBBE (U)

Obuvunaanyizibwa ___________________________________

Omukono gwo'omuzadde(signature)_______________________

Date of consent (IDATE): |__|__|/|__|__|/|__|__|__|__|
dd / mm / yyyy

Omwanawo naye ajja kuyisibwa mu kiwandiiko ky'okukkiriza bwanaaba naye akkirizza.

Omuzadde atasobola kusoma na kuwandiika: Omuzade nga tasobola kusoma na kuwandiika omujulizi yateekako omukono, omujulizi eno bwe kiba kisobose omuzadde ya mwerondera era tasaanye kuba nga alina akakwate konna n'abakola ku kunoonyereza kuno.

Wandiika (Print) Erinnya ly'omujulizi ________________________

Ekyenkumu ky'omuzadde

Omukono (Signature) g'omujulizi________________________

Enakku z'omwezi ________________ Day/month/year

To be completed by the researcher
I confirm that the individual has given consent freely.
Name of researcher: ______________________ Date:|__|__|/|__|__|/|__|__|__|__|
dd / mm / yyyy

Signature: ___________________
