# Supplementary material for: Menstrual health interventions, schooling, and mental health problems among Ugandan students (MENISCUS): study protocol for a school-based cluster-randomised trial
Source: Trials. 2022 Sep 7;23:759. doi: 10.1186/s13063-022-06672-4 (PMC9449307; doi:10.1186/s13063-022-06672-4)
Supplement: Supplementary file 2 — Additional file 2. [file 13063_2022_6672_MOESM2_ESM.zip › ANNEX4~2R1.PDF]

Olupapula ololiko amawulire eri abakozi, oba abalabilira omwana ku ssomero ewagenda okukolebwa okunonyereza kwa MENISCUS okufuna ettu elilimu ebikozesebawa mu nsonga zabakyala.

**Project title:**	Menstrual health interventions, schooling and mental health symptoms among Ugandan students (MENISCUS): a school-based cluster-randomised trial
**Funder:**	UK Joint Global Health Trials (Medical Research Council-Department for International Development-Wellcome Trust) Grant # MR/V005634/1
**Research Site:**	Wakiso and Kalungu Districts<br>C/o MRC/UVRI and LSHTM Uganda Research Unit<br>Plot 51-59, Nakiwogo Road<br>P O Box 49, Entebbe, Uganda<br>Tel: +256(0) 417 704000; (0)312 262910/1; (0)702 438487
**Principal Investigators:**<br><br>**Local Principal Investigator:**	**1. Prof Helen Weiss,**<br>Professor of Epidemiology and Director of the MRC Tropical Epidemiology Group, London School of Hygiene and Tropical Medicine (LSHTM), UK<br>*Email: helen.weiss@lshtm.ac.uk*<br>**2. Prof Janet Seeley**<br>Professor of Anthropology and Health, London School of Hygiene and Tropical Medicine (LSHTM), UK<br>and Head of Social Science Programme, MRC/UVRI and LSHTM Uganda Research Unit<br>*Email: janet.seeley@lshtm.ac.uk*
**Trial Manager:**	Dr. Catherine Kansiime,<br>MRC/UVRI and LSHTM Uganda Research Unit<br>*Email:Catherine.Kansiime@mrcuganda.org*

Mu bufunze (By'olina okumanya ku kunoonyereza kuno):

- Ekigendererwa ky'okunoonyereza kwa MENISCUS kwe kumanya oba nga kinayambako mu kulongoosa eby'okusoma, obubonero obulabirwako eby'obulamu ebikwata ku by'obwongo, okutumbula engeri abaana abawala jebasobola okubeera obulungi nga bali mu nsonga z'ekikyala awamu n'embeela y'obulamu bwabwe mu masomero ga siniya mu wakiso ne kalungu mu Uganda.

- Ekiwandiiko kino kinnyonnyola ekigendererwa ky'okunoonyereza kuno na ki ky'onasabibwa okukola singa onooba okkirizza okukwetabamu.

- Okwetaba kwo mu kunoonyereza kuno kwa kyeyagalire. Dembe lyo okukwetabamu, oba okukwetabamu oluvannyuma n'okuvaamu.

- Kyonna ky'onaaba osazeewo tekijja kukosa ngeri gy'ofunamu bujjanjabi wadde obuyambi.

- Soma ekiwandiiko kino n'obwegendereza era obuuze ekibuuzo kyonna ky'oyagala nga tonasalawo.

Ojja kuweebwa kopi y'ekiwaandiiko kino.

UVRI RESEARCH ETHICS COMMITTEE
VALID UNTIL
26 MAY 2023
APPROVED
P. O. BOX 49, ENTEBBE (U)

Ekitundu ekisooka: Ebikwata ku kunoonyereza kuno.
Enyanjula:

Okunoonyereza kwa **MENISCUS** kukulembeddwamu ekitongole kya **MRC/UVRI** ne Tendekero lya **London School of Hygiene and Tropical Medicine (LSHTM) okuva mu Bungereza** nga bakolera wamu n'ekitongole kya WoMena Uganda.

Tukola okunoonyereza kuno okulungamya amasomero ga siniya okuzuula engeri ezisoboka ez'okuyambamu abaana abawala okubeera abalamu n'okubeera ku somero obulungi nga bali mu nsonga z'ekikyala. Twafunye olukusa okukola okunoonyereza kuno okuva eri abatwala e Somero lino, okuva ku disitirikiti, okuva mu minisitule y'ebyenjigiriza n'emizanyo n'okuva mu bukiiko obulondoola okunoonyereza obwa MRC/UVRI ne LSHTM, wamu ne National Council of Science and Technology.

Tukuyita okwetaba mu kunonyereza kuno. Oli waddembe okusalawo okwetaba oba obutetaba mukunonyereza kuno. Bambi wulira eddembe okutubuuza ebibuuzo kati oba oluvanyuma nga okozesa endagiliro y'okutufuna elambikidwa wamanga. Tujja funa obudde okukunnyonyola.

Ekigendererwa:

Ekigendererwa ky'okunoonyereza kwa MENISCUS kwe kulaba oba nga enkola y'okutumbula eby'obulamu mu mumasomero ga siniya enayambako mu kulongoosa ensonga z'ekikyala (engeri abaana abawala jebasobola okubeera obulungi nga bali mu nsonga z'ekikyala) n'okumanya oba nga kinaayambako mu kulongoosa eby'okusoma, eby'obulamu mubaana abawala awamu n'okumanya kwa baana abalenzi kubikwata kunsonga za bakyala. Okunonyereza kuno bwekunaaba kuvuddemu ebirungi, kujja kutongozebwa mumasomera amalala mu Uganda. Muzimu kunkola z'okunonyereza kuno, abawala bonna mu S2 bajja kuweebwa ettu elirimu ebikozesebwa munsonga zekikyala.

Okulonda.

Tusaba abamu ku bakozi be ssomero n'abazadde b'abayizi mu massomero ga siniya okwetaba mu kunonyereza kwa MENISCUS era bo benyini bafune ettu ly'ebikozesebwa munsonga zekikyala. Olondendwa kubanga i) omwana wo / gw'olabilira muyizi mugamu ku masomero 30 omuli enkola ya MENISCUS, ii) Oli mukozi mulimu ku masomero gano, oba iii) Gwe omu kubenyigira mu Menstrual Health Action Group eyessomero.

Okukwetabamu kwa kyeyagalire.

Oli waddembe okwesalirawo okwetaba mukunonyereza kuno. Osobola okusalawo obutetaba mu kunoonyereza kuno era okusalawo kwo tekujja kukosa mpereeza gwe ne famile yo ze mulina okufuna ku somero wadde ewajjanjabirwa wonna. Oli wa ddembe okutubuuza ebibuuzo byonna byoyagala era tujja kubyanukula. Osobola obutasalawo kati, oli waddembe okusooka okukirowoozaako n'otubuulira oluvannyuma ky'onooba osazeewo. Osobola okusalawo okulekerawo okwetaba mukunonyereza kuno essawa yonna.

Emitendera.

Okunoonyereza kuno kugenda mumaaso n'okukolebwa okuva mu 2021 ne 2023 mu masomero ga siniya 60 agaalondebwa mu disitulikiti ye Wakiso ne Kalungu. Mumasomero 60, amasomero 30 gajja kulondebwa okufuna enkola ya MENISCUS omuli okusomesebwa ku nkyukakyuka z'omubiri nga omwana avubuka ne nsonga zekikyala, okwongera omutindo ku kabuyonjo z'essomero, omukisa okwetaba mu katemba akwatagana nensonga zekikyala wamu nokugaba ettu elirimu ebikozesebawa munsonga zekikyala nga muno muja kubaamu kyeyagalire owokufuna akakopo akeyambisibwa okulembeka omusaayi mukiseera kye nsonga zekikyala, nenkola ezenjawulo okukakanya obulumi.

1) Okugaba ettu ly'ebikozesebwa mu nsonga zekikyala

UVRI-RESEARCH ETHICS COMMITTEE
VALID UNTIL
2 6 MAY 2023
APPROVED
P. O. BOX 49, ENTEBBE (U)

Oja kuweebwa ettu elirimu pads ezisobola okuddamu nezikozesebwa (AFRIpads) ezinagabibwa nga ziri munsawo wamu n'obuwale obwomunda, eccupa y'amazzi, sabbuuni, tawulo ne konteyina eyekuuma. Oja kwetaba mu musomo ogunakulembelwa omutendesi kunkozesa ya pads ezisobola okuddamu nezikozesebwa. Bajja kulaga engeri pads zino gyezikozesebwamu era tujja kubaganya ebirowoozo kunsonga yonna gyonaba olina kumulamwa ogwo. Abakyala abetaba mukunonyereza kuno baja kusabibwa okugezaako okozesa ebikozesebwa munsonga zekikyala okumala omwaka webanaba nga tebakisanzemu buzibu. Ssinga ofuna obuzibu bwona okukozesa ebikozesebawa ebiri mu ttu, ojja kusobola okukyogerako nakulira akabinja kabakozi mu kunonyereza, omutendesi omukugu oba omusawo wa pulojekiti.

2) Okutendekebwa okusomesa abalala kunsonga zekikyala

Bwonooba olondedwa, oyinza okusabibwa okwetaba mukutendekebwa okw'okusomesa abalala kubikwatagana n'ensonga zabakyala era nga mwemuli nenkozesa yettu ly'ebikozesebwa munsonga zabakyala. Okutendekebwa kuno kujja kukolebwa abatendesi abakugu okuva mu WoMena Uganda era nga bajja kusobola okwanukula ebibuuzo oba ensonga yonna gyonaba olina nga tonaba kusomesa balala.

3) Okubuuzibwa ebibuuzo (~60 dakiika)

Oyinza okusabibwa okwogerako ssekinomu n'omunonyereza omutendeke nga akubuuza ebibuuzo kubikwatagana nebyoyiisemu nga wenyigira mu Action Group, okutendeka abalala kunsonga zabakyala, ne'kunkozesa y'ebikozesebwa munsonga zabakyala. Okwogerako n'omunonyereza kujja kubawo mukiffo ekikkanyizidwako oba mu ssomero mwenyini. Okwogera kuno kwonna kuyinza okukwatibwa ku butambi. Obutambi bujja kuumibwa bulungi mukabada ensibire ddala gulugulu ku MRC/UVRI ne LSHTM Uganda Research Unit. Obubaka obunaba bukwatidwa kubutambi bwa kyaama era tewali muntu yenna okujako abanonyereza oba abantu abalala abakilizidwa mumateeka nga abo abekenenya obubaka mubutambi (transcribers), omuvujilizi w'okunonyereza oba akakiiko akavunanizibwa okukwasa empisa mu kunonyereza akalina obuyinza obwekigero okufuna obutambi, bebajja okukilizibwa okuwuliriza obubaka obuli ku butambi buno.

Obuzibu n'obutawulira mirembe: Okunonyereza kubi oba kwabulabe gyooli?

Tuyinza okusaba okugabanako naffe ebimu kubikwatako nga omuntu okugeza, nga by'oyiseemu nga wenyigira mu Menstrual Health Action Group ku ssomero mwokola oba ely'omwana wo, n'endowooza zo kuby'ensonga z'abakyala. Oyinza obutawulira mirembe kwogera kunsonga ezo ezimu. Oja kusomesebwa kungeri yokwoza n'okwanika pads ezisobola okuddamu nezikozesebwa, okumanya obubonero singa oba ofunye obulwadde, negwoyinza okutukilira. Ssinga ebiragiro bino tebigobelerwa, waliwo obulabe obw'okufuna endwadde oba okuyisibwa obubi nga okozesa pads zino. Ssinga wabelawo obuzibu nga okozesa pads ezisobola okudamu nezikozesebwa, osobola okutukilira omusawo we ssomero okufuna obuyambi oba okuyambako okufuna woyinza okusanga obuyambi. Tulina omusawo aja okutukilirwa ssinga obuzibu obufunidwa. Okubilizibwa okuloopa mangu ddala obuzibu bwonna bwofuna nga okozesa ettu ly'ebikozesebwa mu nsonga zekikyala.

Okuganyurwa: waliwo ekintu ekirungi kyonna ekinabawo gyoli olwokwetaba mu kunonyereza?

Okwetabako mu kunonyereza kwandituyamba, amasomero, amalwaliro, n'abobuyinza mu by'enjigiriza n'ebyobulamu okwongera okumanya ku bulamu bwo n'empeereza ze wetaaga. Tulina esuubi nti kino kijja kuyamba abantu bonna okufuna ebyetaago ebyo mungeri esiinga gy'ebujja maaso eyo.

Okusasulibwa olw'ebiseera: Onafuna ekintu kyonna olwokwetaba mu kunonyereza?

UVRI RESEARCH ETHICS COMMITTEE
VALID UNTIL
26 MAY 2023
APPROVED
P. O. BOX 49, ENTEBBE (U)

Oja kuweebwa shillings 10,000 okuliyilira obudde bwo mukunonyereza n'okwetaba mu kuwa obubaka obukwatagana n'okunonyereza. Era oja kufuna okutendekebwa mu nsonga z'ekikyala, eby'okunywa ebiweweeza mu kutendekebwa n'okuddizibwa ssente z'entambula singa ekutendekebwa kunategekebwa wabweru w'essomero.

Emmizi (Confidentiality): Ebintu bino binaamanyibwako abantu abalala?

Tewali gwe tujja kubuulirako nti weetabye mu kunoonyereza kuno. Tewali muntu yenna atakola mu kunoonyereza kuno gwe tujja kubuulirako ku bikwatako. Byonna ebikwatako bijja kubako ennamba eyekyaama (study number) mu kifo ky'elinnya lyo. Wabula ebikwatako biyinza okulabibwako abekeneenya okunonyereza (Auditor).

Okutegeezebwa ebinaazuulibwa mu kunoonyereza: Onootegeezebwa ebinaazuulibwa mu kunoonyereza kuno?

Okunonyereza kuno nga kuwedde, tujja kubulira ebizuliddwa. Tujja kugabana ebizuliddwa ne byetuyize na bazadde / abavunanizibwa ku baana, abobuyinza mu ssomero, e'bibuga ne kumutendera gwe gwanga okutwaliza awamu.

Oluvanyuma, tujja kubulira abantu abalala, banasayansi, abasawo, nabalala byetunaba tuzudde. Kino tujja kikola nga tuwandiika no kugabana z'alipoota, n'okuba n'enkiiko nabantu abalina ekigendelerwa mu kunonyereza kuno. Ebizuliddwa mukunonyereza kuno era bija kubibwa mu butabo bwasayansi obwensi yonna ne kumikutu egy'emitimbagano abantu abalala basobole okutuyigirako. Nga ojeeko abanonyereza, ebizuliddwa tebijja kugabanibwa mungeri eraga biki byenyini byewatugamba oba byetwakufunako mukunonyereza nga ssekinomu. Obubaka bw'okunonyereza kuno buyinza okutekebwa kumukutu ogukunganya obubaka obwenjawulo obuva mukunonyereza ogugatta abantu bonna ogwa London School of Hygiene and Tropical Medicine. Kino kitegeeza nti obubaka buno buyinza okukozesebwa mumaaso eyo okwekenenya ensonga endala. Obubaka bwonna buja kuba bwakyaama i.e. teli aja kumanya nti bukwata kugwe.

Okwebuuza: Ani gw'oyinza okw'ogerako naye oba okubuuza ebikwata ku kunoonyereza kuno?

Osobola okutubuuza ekibuuzo kyonna kati oba gyebuja nga oyita ku ssimu, e-mail, nga owereza ebbaluwa oba nga otutukilira kundagiliro elambikidwa kukiwandiiko ekigenda okuweebwa. Woba oli kumpi, osobola okututukilira.

Osobola okutukilira bano wamanga kubikwatagana n'okunonyereza kuno.

a) Dr.Catherine Kansiime, MENISCUS trial Project Lead
   Email: catherine.kansiime@mrcuganda.org, nnamba yessimu: +256 702438487

Woba olina ebibuuzo byonna, okwemulugunya oba ensonga yonna kubikwatagana ne ddembe lyo nga omuntu eyetabye mukunonyereza kuno, bambi tukilira UVRI Research Ethics Committee ku nnamba yessimu: +256 0414 321962 oba +256 716 321962

UVRI RESEARCH ETHICS COMMITTEE
VALID UNTIL
26 MAY 2023
APPROVED
P. O. BOX 49, ENTEBBE (U)

EKITUNDU II: Okukiriza (VERSION 1.0 JANUARY 2022)

Nga ntekakao omukono wamanga, nzikiriza okwetaba mukunonyereza kuno nga wekunyonyoledwa waggulu, omuli:

- Okufuna ettu ly'ebikozesebwa munsonga zabakyala nokufuna okutendekebwa kunkozesa yalyo.
- Okwetaba mukwanukula ebibuuzo ebya ssekinomu no munonyereza bwenaba nondedwa
- Okukozesa obubaka obwekyaama obukunganyizidwa mu kunonyereza era n'okubugabana n'abanonyereza abalala.

Ebibuuzo byange ebikwatagana n'okunonyereza kuno byanukudwa......................................

**Bambi soma buli kibuuzo wamanga**	**Saaza kwebyo byokiriziganya nabyo**	
Osomye oba osomedwa ebikwata ku kunonyereza kuno?	yee	Nedda
Waliwo omuntu omulala yenna akunyonyodde ku kunonyereza kuno?	Yee	Nedda
Otegedde bulungi okunonyereza kuno kyekukwattako?	Yee	Nedda
Ebibuuzo byo ku kunonyereza kuno bididwamu bulungi?	Yee	Nedda
Otegedde bulungi nti oli wadembe okuva mukunonyereza kuno ?	Yee	Nedda
Oli musanyufu okukiriza okwetaba mukunonyereza kuno?	Yee	Nedda

Erinya ly'eyetaba mukunonyereza: ________________________________ School ID: |__|__|__|

Omukono gw'eyetaba mukunonyereza : ____________________

Date of consent (dd/mm/yyyy): |__|__|/|__|__|/|__|__|__|__|

Atasobola kusoma nakuwandiika: omujulizi nga asobola okusoma n'okuwandiika wakusako omukono (wekiba kisoboka, omuntu ono alina okulondebwa oyo eyetaba mukunonyereza era talina kuba nakakwate n'abanonyereza). Abazadde oba abavunanizibwa kubaana abatasobola kusoma na kuwandiika balina okusako ekyenkumu.

Wandiika (Print) erinya lyo mujulizi ______________________ **AND**

Ekyenkumu kyoyo eyetaba mukunonyereza

Omukono gwo mujulizi _______________________

Ennaku z'omwezi _____________________ Day/month/year

Omunonyereza wakujjuzamu bino wammanga: Nkakasa nti eyetabye mukunonyereza abade waddembe okutuwa olukusa.

Erinya ly'omunonyereza______________________Ennaku z'omwezi |__|__|/|__|__|/|__|__|__|__|

Omukono: ______________________________ dd / mm / yyyy

UVRI-RESEARCH ETHICS COMMITTEE VALID UNTIL 26 MAY 2023 APPROVED P. O. BOX 49, ENTEBBE (U)

UVRI-RESEARCH ETHICS COMMITTEE
VALID UNTIL
26 MAY 2023
APPROVED
P. O. BOX 49, ENTEBBE (U)
