# Supplementary material for: Menstrual health interventions, schooling, and mental health problems among Ugandan students (MENISCUS): study protocol for a school-based cluster-randomised trial
Source: Trials. 2022 Sep 7;23:759. doi: 10.1186/s13063-022-06672-4 (PMC9449307; doi:10.1186/s13063-022-06672-4)
Supplement: Supplementary file 2 — Additional file 2. [file 13063_2022_6672_MOESM2_ESM.zip › ANNEX4~3R1.PDF]

MRC/UVRI and LSHTM Uganda Research Unit

MRC Medical Research Council — Uganda Virus Research Institute — London School of Hygiene & Tropical Medicine

Information and consent form for parents/guardians of students at a MENISCUS trial, for student to receive a menstrual health kit

**Project title:**	Menstrual health interventions, schooling and mental health symptoms among Ugandan students (MENISCUS): a school-based cluster-randomised trial
**Funder:**	UK Joint Global Health Trials (Medical Research Council-Department for International Development-Wellcome Trust) Grant # MR/V005634/1
**Research Site:**	Wakiso and Kalungu Districts<br>C/o MRC/UVRI and LSHTM Uganda Research Unit<br>Plot 51-59, Nakiwogo Road<br>P O Box 49, Entebbe, Uganda<br>Tel: +256(0) 417 704000; (0)312 262910/1; (0)702 438487
**Principal Investigators:**<br><br><br><br>**Local Principal Investigator:**	**1. Prof Helen Weiss,**<br>Professor of Epidemiology and Director of the MRC Tropical Epidemiology Group, London School of Hygiene and Tropical Medicine (LSHTM), UK<br>*Email: helen.weiss@lshtm.ac.uk*<br>**2. Prof Janet Seeley**<br>Professor of Anthropology and Health, London School of Hygiene and Tropical Medicine (LSHTM), UK<br>and Head of Social Science Programme, MRC/UVRI and LSHTM Uganda Research Uni<br>*Email: janet.seeley@lshtm.ac.uk*
**Trial Manager:**	Dr. Catherine Kansiime,<br>MRC/UVRI and LSHTM Uganda Research Unit<br>*Email:Catherine.Kansiime@mrcuganda.org*

Summary (What you should know about this study):

- The aim of the trial is to assess whether the intervention ("MENISCUS") improves educational attainment, mental health symptoms, menstrual management and quality of life outcomes among girls in secondary school in Wakiso and Kalungu districts in Uganda.
- This document explains the purpose of this study and what your child will be asked to do if you agree for them to participate.
- Your child's participation is completely voluntary. You or your child have the right to decide to not take part in the study or to agree to take part now and change your mind later.
- Whatever you decide will not affect your or your child's regular healthcare and support.
- Please review this form carefully. Ask any questions before you make a decision.

You will be given a copy of this form to keep.

UVRI-RESEARCH ETHICS COMMITTEE
26 MAY 2023
APPROVED
P. O. BOX 49, ENTEBBE (U)

Part I: Information about this study
Introduction
The MENISCUS trial is led by the MRC/UVRI and LSHTM Uganda Research Unit in Entebbe and the London School of Hygiene and Tropical Medicine in United Kingdom, with our partner WoMena Uganda.

We are carrying out research to guide our secondary schools to identify practical ways of helping girls to become and stay healthier and complete studying at school through improved management of menstrual periods. We have received permission to conduct this research from school administration, the district, the Ministry of Education and Sports, and the Research Ethics Committees of the UVRI, LSHTM and Uganda National Council of Science and Technology (UNCST).

We invite your child to be part of this research. It is optional for you to choose whether or not you want your child to participate in this research. Please feel free to ask us questions now or later using our contact information which is indicated below. We will take time to explain to you.

Purpose
The purpose of the MENISCUS trial is to see whether a health promotion intervention in secondary schools improves menstrual health (i.e. how girls manage their periods safely and confidently). We want to learn whether the package is likely to improve educational, health, and well-being outcomes among girls, and menstrual health knowledge and attitudes towards periods among boys. If the intervention is successful, it could be introduced in other schools in Uganda.

Selection
Your child has been selected to participate in this research because they are a student in one of the 30 MENISCUS intervention schools and are either i) participating in the school's Menstrual Health Action Group or ii) a senior student selected to be trained to offer peer-support around menstrual health.

Voluntary Participation
It is optional for your child to participate in this research. You or they can choose to say no. That decision will not affect any services that you and your family receive at the secondary school and/or health facilities. You can ask as many questions as you like and we shall be available to answer them. You don't have to decide today. You can think about it and tell us what you decide later. You or your child can also choose to stop participating at any time.

Procedures
This research is being conducted between 2021 and 2023 primarily in 60 identified secondary schools in Wakiso and Kalungu Districts. Of these, 30 schools will be randomly selected to receive the MENISCUS intervention which includes education about puberty and menstruation, improvements to school toilet facilities, the opportunity to participate in a drama skit related to menstrual health, and provision of a menstrual health kit and pain management strategies.

1) <u>Provision of Menstrual Management kit</u>
Your child will be offered a kit which contains reusable sanitary pads (AFRIpads) provided in a bag with knickers, a water bottle, soap, a towel and a metal container.

They will participate in a session led by a trainer on how to use re-usable pads. The trainer will show your child how the re-usable pads are used and will discuss any concerns they might have about this. Female participants will be asked to try using the menstrual products for the next year, if they are comfortable doing so. If they are experiencing any problems using any of the products in the menstrual health kit, they will be able to discuss it with the team leader, the expert trainer or the clinical officer on the project.

UVRI-RESEARCH ETHICS COMMITTEE
VALID UNTIL
26 MAY 2023
APPROVED
P. O. BOX 49, ENTEBBE (U)

2) <u>Training to teach others about menstrual health</u>
If selected, your child may also be asked to participate in a training on how to teach others about menstrual health, including on use of the menstrual health kit, and how to provide peer support around menstrual health management. This training will be led by experienced WoMena Uganda trainers who will be able to answer any questions or concerns they may have before they teach others.

3) <u>Individual interviews (~60 minutes)</u>:
Your child may be requested to have an individual interview with a trained researcher, to ask about their experience of participating in the Action Group, menstrual health training, and/or using the menstrual products. The discussion will take place at an agreed venue or within the school premises. The entire discussion may be tape-recorded. The tapes will be kept securely in lockable cabinets/cupboards at MRC/UVRI and LSHTM Uganda Research Unit. The information recorded is confidential, and no one else except the researchers or other ethical eligible person(s) such as the transcribers, the sponsor or ethics review committees with regulated access to the tapes will be allowed to listen to the tapes.

Risks and discomfort: Is the study bad or dangerous for your child?
We may ask your child to share with us some personal information, such as their experience as part of the Menstrual Health Action Group at their school, and/or their attitudes towards menstruation. They may feel uncomfortable talking about some of these topics.

Your child will be taught how to wash and dry the re-usable sanitary pads, identify symptoms of infection and whom to report these to. If these instructions are not followed, there is a risk of infection or irritation when using the pads. However, in case of any adverse events or problems with the reusable pads, you or your child can contact the school nurse for assistance or for referral. We have a clinical officer to whom adverse events will be referred. Your child is encouraged to report early any challenges they experience with using the menstrual health kit.

Benefits: Is there anything good that happens to your child from participating?
Your child's participation is likely to help us, the schools, health facilities and the education and health authorities to find out more about your health and service needs. We hope that these will help all the relevant people to meet those needs better in the future.

Reimbursements: Will you receive anything for your child being in the study?
Your child will not be paid to take part in this research. However, if selected for an individual interview, they will be given a pen, a hardcover note book and a soft drink to compensate for their time and effort. You will be given 10,000 shillings to compensate for your time and effort.

Confidentiality: Is anybody going to know about this?
We will not tell other people that your child was involved in this research. We shall not share personal information that identifies them to anyone who does not work in this research. Any information about your child will have a study number on it instead of their name. However, their data may be seen by auditors.

Sharing the findings: Will you be told the study results?
When this research is completed, we shall inform your child about the results obtained. Then we shall share the research results with parents/guardians, authorities at the school, municipal and national levels, including what we have learnt.

Afterwards, we will be telling other people, scientists, health workers and others, what we found. We will do this by writing and sharing reports and by going to meetings with people who are interested in

UVRI-RESEARCH ETHICS COMMITTEE
VALID UNTIL
28 MAY 2023
APPROVED
P. O. BOX 49, ENTEBBE (U)

this work. The research findings will also be published in international science journals and electronic websites so that other people may learn from us. However, the results will never be reported in a way that allows anyone except members of the research team to know what your child specifically told us or any of the individual results we obtained from them. Data may be made available in the public domain via the London School of Hygiene and Tropical Medicine data repository. This means that it may be used for further analyses. All data will be anonymised i.e. it cannot be linked to your child.

Who to contact: Who can you talk to or ask questions about this study?
You can ask us questions now or later by telephone, e-mail, post or at the physical addresses indicated on the assent/consent form to be given to you. If you are nearby, you can come and see us.

You can contact the following about this research:

a) Dr.Catherine Kansiime, MENISCUS trial Project Lead
   Email: catherine.kansiime@mrcuganda.org Phone number +256 702438487

If you have any questions, complaints or concerns about your rights as a person involved in this research, please contact: UVRI Research Ethics Committee: Phone number +256 0414 321962 or +256 716 321962

UVRI-RESEARCH ETHICS COMMITTEE
VALID UNTIL
26 MAY 2023
APPROVED
P. O. BOX 49, ENTEBBE (U)

PART II: Consent form (VERSION 1.0 JANUARY 2022)

By signing below I consent for my child to participate in the study as described above, including:

- To receive a menstrual management kit and training on how to use it
- To participate in an individual interview if selected for this activity
- For all anonymised data collected to be used as part of the research and shared with other researchers

My questions concerning this study have been answered by ……………………………….

Please read each question below	Please circle all you agree with:	
Have you read (or had read to you) information about this project?	Yes	No
Has somebody else explained this project to you?	Yes	No
Do you understand what this project is about?	Yes	No
Have you had any questions answered in a way you understand?	Yes	No
Do you understand that it is ok to stop taking part at any time?	Yes	No
Are you happy to take part in this study? [CONSENT]	Yes	No

Name of participant: ______________________________ School ID: |__|__|__|

Name of parent/guardian: ______________________________

Signature of parent/guardian: ____________________

Date of consent (dd/mm/yyyy): |__|__|/|__|__|/|__|__|__|__|

If literacy challenged: A literate witness must sign (if possible, this person should be selected by the participant and should have no connection to the research team). Literacy challenged parents/guardians should include their thumb print as well.

Print name of witness____________________ **AND** **Thumb print of participant**

Signature of witness ____________________

Date ____________________ **Day/month/year**

To be completed by the researcher: I confirm that the individual has given consent freely.

Name of researcher: ____________________ Date:|__|__|/|__|__|/|__|__|__|__|

dd / mm / yyyy

Signature: ____________________

UVRI-RESEARCH ETHICS COMMITTEE
VALID UNTIL
★ 26 MAY 2023 ★
APPROVED
P.O. BOX 49, ENTEBBE (U)
